# Ancient Lowland Maya neighborhoods: Average Nearest Neighbor analysis and kernel density models, environments, and urban scale

**DOI:** 10.1371/journal.pone.0275916

**Published:** 2022-11-02

**Authors:** Amy E. Thompson, John P. Walden, Adrian S. Z. Chase, Scott R. Hutson, Damien B. Marken, Bernadette Cap, Eric C. Fries, M. Rodrigo Guzman Piedrasanta, Timothy S. Hare, Sherman W. Horn, George J. Micheletti, Shane M. Montgomery, Jessica Munson, Heather Richards-Rissetto, Kyle Shaw-Müller, Traci Ardren, Jaime J. Awe, M. Kathryn Brown, Michael Callaghan, Claire E. Ebert, Anabel Ford, Rafael A. Guerra, Julie A. Hoggarth, Brigitte Kovacevich, John M. Morris, Holley Moyes, Terry G. Powis, Jason Yaeger, Brett A. Houk, Keith M. Prufer, Arlen F. Chase, Diane Z. Chase

**Affiliations:** 1 Department of Geography and the Environment, The University of Texas at Austin, Austin, TX, United States of America; 2 Department of Anthropology, Harvard University, Cambridge, MA, United States of America; 3 Mansueto Institute Postdoctoral Fellow and Department of Anthropology Postdoctoral Scholar, The University of Chicago, Chicago, IL, United States of America; 4 Department of Anthropology, University of Kentucky, Lexington, KY, United States of America; 5 Department of Anthropology, Bloomsburg University of Pennsylvania, Bloomsburg, PA, United States of America; 6 Department of Anthropology, University of Texas at San Antonio, San Antonio, TX, United States of America; 7 Department of Anthropology, University of Nevada, Las Vegas, Las Vegas, NV, United States of America; 8 Department of Anthropology, University of Central Florida, Orlando, FL, United States of America; 9 Department of Sociology, Social Work, and Criminology, Morehead State University, Morehead, KY, United States of America; 10 MesoAmerican Research Center, University of California, Santa Barbara, Santa Barbara, CA, United States of America; 11 Department of Anthropology and Archaeology, University of Calgary, Calgary, AB, Canada; 12 Department of Anthropology-Sociology, Lycoming College, Williamsport, PA, United States of America; 13 School of Global Integrative Studies, University of Nebraska, Lincoln, Lincoln, NE, United States of America; 14 Department of Anthropology, University of Toronto, Toronto, ON, Canada; 15 Department of Anthropology, University of Miami, Coral Gables, FL, United States of America; 16 Department of Anthropology, Northern Arizona University, Flagstaff, AZ, United States of America; 17 Department of Anthropology, University of Pittsburgh, Pittsburgh, PA, United States of America; 18 Department of Anthropology, Galen University, Cayo, Belize, C.A; 19 Department of Anthropology, University of New Mexico, Albuquerque, NM, United States of America; 20 Department of Anthropology, Baylor University, Waco, TX, United States of America; 21 Institute of Archaeology, National Institute of Culture and History, Belmopan, Belize, C.A; 22 Department of Anthropology and Heritage Studies, University of California, Merced, Merced, CA, United States of America; 23 Department of Anthropology, Kennesaw State University, Kennesaw, GA, United States of America; 24 Department of Sociology, Anthropology, and Social Work, Texas Tech University, Lubbock, TX, United States of America; 25 Department of Anthropology, Pomona College, Claremont, CA, United States of America; 26 Vice President for Academic Innovation, Student Success, and Strategic Initiatives, Claremont Graduate University, Clermont, CA, United States of America; New York State Museum, UNITED STATES

## Abstract

Many humans live in large, complex political centers, composed of multi-scalar communities including neighborhoods and districts. Both today and in the past, neighborhoods form a fundamental part of cities and are defined by their spatial, architectural, and material elements. Neighborhoods existed in ancient centers of various scales, and multiple methods have been employed to identify ancient neighborhoods in archaeological contexts. However, the use of different methods for neighborhood identification within the same spatiotemporal setting results in challenges for comparisons within and between ancient societies. Here, we focus on using a single method—combining Average Nearest Neighbor (ANN) and Kernel Density (KD) analyses of household groups—to identify potential neighborhoods based on clusters of households at 23 ancient centers across the Maya Lowlands. While a one-size-fits all model does not work for neighborhood identification everywhere, the ANN/KD method provides quantifiable data on the clustering of ancient households, which can be linked to environmental zones and urban scale. We found that centers in river valleys exhibited greater household clustering compared to centers in upland and escarpment environments. Settlement patterns on flat plains were more dispersed, with little discrete spatial clustering of households. Furthermore, we categorized the ancient Maya centers into discrete urban scales, finding that larger centers had greater variation in household spacing compared to medium-sized and smaller centers. Many larger political centers possess heterogeneity in household clustering between their civic-ceremonial cores, immediate hinterlands, and far peripheries. Smaller centers exhibit greater household clustering compared to larger ones. This paper quantitatively assesses household clustering among nearly two dozen centers across the Maya Lowlands, linking environment and urban scale to settlement patterns. The findings are applicable to ancient societies and modern cities alike; understanding how humans form multi-scalar social groupings, such as neighborhoods, is fundamental to human experience and social organization.

## Introduction

Large human settlements often include subdivisions of smaller communities based on shared interests, identities, or living spaces. Within modern cities, smaller sub-communities include wards (e.g., Chicago), boroughs (e.g., New York City (NYC), London), districts, and neighborhoods (e.g., SoHo, Upper East Side, and Greenwich Village in the Manhattan borough of NYC). Modern cities are diverse in their structures and layouts, being influenced by geographic features, cultural shifts, socioeconomic inequities, racial/ethnic disparities, and historical contingencies dating back to the earliest foundations of the cities. However, neighborhoods today form diverse social sub-communities within cities, often resulting in a sense of social solidarity, collective identity, or camaraderie among occupants [1]. Neighborhoods, or groups of co-located residences with frequent, repeated face-to-face social interactions and shared identities [see 2–4], exist in present and past societies alike. Subdivisions within cities vary in size and often have multiple, overlapping functions that are frequently fluid, change over time, or differ on a person-by-person basis. Nonetheless, these smaller socio-spatial units compose an integral part of our cities today and did in the past as well.

Yet, identifying neighborhoods, or smaller socio-spatial units, archaeologically remains a challenge and neighborhoods are one of the least investigated aspects of Maya studies [5, 6]. Scholars have relied on a variety of qualitative and quantitative methods to delineate neighborhoods of the past [see overviews in 7, 8]. Even within a single region, the Maya Lowlands, neighborhoods have been identified using spatial analyses [9–12], artifact assemblages [13–16], and architectural remains [17–19]. Additionally, those approaches exhibit diversity in the neighborhoods they identify. With many ways to model past neighborhoods, it often becomes unclear whether we are comparing apples to oranges. This may be an especially problematic situation given that ancient Maya settlement clustering occurred on at least three hierarchically nested levels–neighborhoods, districts, and cities following ME Smith [3], or clusters, minor centers, and major centers following Bullard [20]. Moreover, we lack a holistic understanding of how a single approach or method works across the diverse Lowland Maya settlement landscape composed of political centers of varying size and density [see 21]. However, the application of a procrustean, “one-size-fits-all” approach can mask variability within ancient cities and neighborhood composition. Subsequently, any approach needs to be sufficiently standardized to reliably chart regional variability in a comparative fashion, while also being sensitive enough so as not to mask place-specific and local-level variability. Another criterion for comparative analysis is that the method should be easily replicable by multiple researchers with their respective settlement datasets.

This article collaboratively assesses ancient Lowland Maya neighborhoods using a single method of identification across 15 research projects. First, we use geospatial analysis to identify whether Maya centers are composed of clusters of households; and, if so, the degree to which they are clustered. We work under the premise that clusters of households represent a type of neighborhood, although recognize that the totality of diversity in neighborhood composition is beyond the scope of this method. Quantitative and qualitative approaches are then used to assess the extent to which the clusters form potential neighborhoods in different contexts. The presence of spatially delineated neighborhoods is based on the distance interaction principle, the notion that people in closer proximity interact more frequently [22, 23, see also 24], with “social glues” such as economic cooperation, kin-classifications, and collective events reaffirming these relationships. Additional structural factors that influence variability in spatial clustering, neighborhood size, and community formation include local/regional physiography, environmental zone, size of settlement area, settlement density, population, local customs, and the political power of elites.

**Fig 1:**
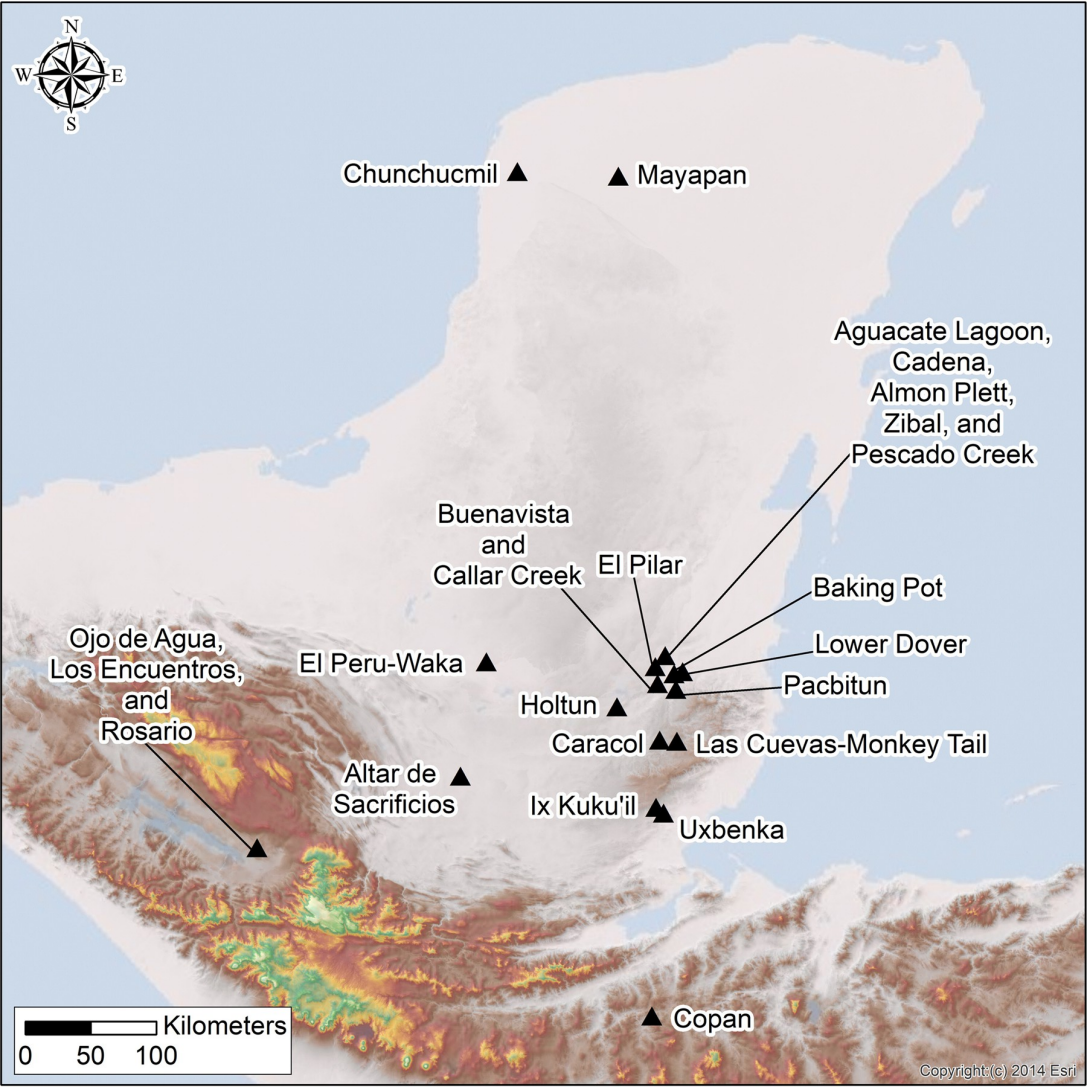
Map of Maya area, showing locations of centers used in this study. Basemaps include a 30 m SRTM DEM freely available for download from USGS Earth Explorer website and a hillshade image that is the intellectual property of Esri and is used herein under license. Copyright 2014 Esri and its licensors. All rights reserved. (Map by AET).

We evaluate the clustering of households to identify potential neighborhood boundaries at 23 centers composed of monumental civic ceremonial architecture and surrounding households that vary in size in the Maya Lowlands (Fig 1). All centers were analyzed using the same unit of analysis: residential groups. Classic Maya residential groups typically comprise one or more domestic structures situated around a central space, sometimes further delineated by walls (*albarradas*), topography such as small knolls, or discrete hilltops. Centers vary in size, from small centers with modest ceremonial facilities to expansive cities (larger centers); taken together, we analyze a dataset of more than 24,500 residential groups in this study. We assessed the clustering of residential groups using the Average Nearest Neighbor (ANN) Analysis and a Kernel Density (KD) model in ArcGIS [see 11]. When possible, potential neighborhoods were designated using the KD output derived from the ANN results and qualitative classifications such as topography and hydrology and anthropogenic features like road systems or wells.

The significance of this research is threefold. First, we provide quantitative analyses of a large dataset of Lowland Maya centers using ANN and KD [25, 26]. These methods are reproducible at any center with comparable data, allowing others to use our results for future research. Second, through our analyses, we find that nearly all Maya centers were clustered to some degree, confirming a phenomenon qualitatively noted for nearly a century [27] yet never quantitatively assessed using the same method, here the ANN analysis. Furthermore, all Maya centers had at least some multi-scalar social units including the political center, districts, neighborhoods, and face blocks, which we define below. The intermediate unit of the neighborhood bonds households together; usually such neighborhoods were comprised of people of differing kin groups who likely interacted daily and relied on each other for a variety of tasks. These individuals lived in households, long considered the primary context of social reproduction in hierarchical societies, which, at least for Maya commoners, represents a “long-term durable container through which generations of kin cycled” based on mortuary patterns and chronological sequences [28]. Households are the foundational units of neighborhoods, and their kin and corporate structures are fundamental to daily neighborhood interactions.

Finally, we note that while Maya centers contained multi-scalar units, diversity exists in the layout, density, and spatial extent of these units, due in part to physiographic and environmental conditions, agricultural practices, settlement scale, the relative political power of local elites and higher-level suzerains, culturally constructed beliefs about landscape and habitation, sociality and kinship, cooperation and collective action, and other variations in human behaviors. While the methods used here are but one subset of many types of spatial analysis used to identify clustering in settlement patterns and delineate possible neighborhoods, the approach we use is relatively simple and permits multiple scholars with differing degrees of experience with spatial analysis and different settlement datasets to contribute to a broader study. Our research lays the foundation for future collaborative work in studying past human behaviors across the Maya region by using geospatial analyses of settlement patterns to understand the 99% of the population who lived modest lives in humble houses [*sensu* 29].

### Identification and definitions of neighborhoods in archaeological contexts

Settlements and centers vary in their size, scale, density, layouts, and distributions of households. Within modern cities, we designate neighborhoods based on spatial boundaries such as roads, rivers, or topography or on shared characteristics such as specific types of architecture, shops and restaurants, religious or ethnic identity, social memory, and socioeconomic status [30, 31]. Neighborhoods can be “politically based” or historically-based delineations (see for example, [32]), which may appear arbitrary to later inhabitants unaware of the historical narratives. Some modern neighborhoods show little change over time, whereas others can undergo rapid transitions through processes like gentrification and degradation [e.g., 33]. We can use studies of modern neighborhoods as simple analogies to help guide our identification of ancient neighborhoods. Identifying neighborhoods through potential markers of shared identity is ideal [*sensu* 34, see also 35, 36], but this approach requires extensive excavation data that may not be readily available. Therefore, many archaeologists use a combination of spatial methods with architectural and artifactual data to identify small social units within ancient cities [2, 3].

Neighborhoods are spatially discrete areas formed primarily through frequent face-to-face interaction [3, 5, 8, 9, 37, 38]. Neighborhoods might be evident through specific shared physical or social characteristics that are archaeologically visible. The clusters in which we are specifically interested are generally smaller than districts, which contain administrative or civic ceremonial functions in addition to residences [3, 39, 40]. Neighborhood level units may arise from a range of social behaviors, including kinship, religion, administrative needs, economic cooperation, and defense, among other reasons [1, 41]. Households clustering into social units such as neighborhoods is a seemingly ubiquitous feature of ancient complex societies. Examples exist in the ancient Near East [42, 43], the Andes [44–46], North America [47], and the Indus Valley [48].

Here, we define different aspects of settlements that can be used for future comparative analyses. Building on previous work, we provide definitions of the units mentioned in this paper, beginning broadly with centers and narrowing the spatial scale to households.

- **Centers**–a blanket term to refer to settlements (or sites, polities, or political centers) of various sizes that include both the civic-ceremonial architecture and surrounding residences. They vary in size along a continuum from smaller to larger centers but importantly contain some monumental architecture and surrounding households and maintain some degree of political autonomy. Some centers may incorporate others as they grow over time (e.g., Caracol; [49]).
- **Communities**–groups of people with shared (social) attributes at any spatial scale. This super-set includes neighborhoods, districts, cities, but also includes non-spatially co-located groups with shared practices, traits, or beliefs [50, 51]. Communities can be larger or smaller than a neighborhood [2].
- **Cities–**large centers characterized by monumental architecture usually located in a central area and containing heterogeneous populations with specialization and status differences, large populations, and high density [52–54].
- **Social District**–a social unit smaller than the center or city but larger than a neighborhood [3, 4]. Social districts encompass multiple neighborhoods wherein residents share "something in common." i.e., physical resources, similar identities, affective ties, patterns of interaction, or material styles [2, 3].
- **Administrative District / Ward**–a top-down administrative unit within a center or city that may have identifiably unique civic architecture within it [2, 3, 55]. Usually composed of multiple neighborhoods.
- **Household Cluster**–a group of household compounds with "spatial integrity" in the built environment. Household clusters may be good candidates for inclusion within neighborhoods [2].
- **Neighborhood–**a group of co-located residents with frequent, repeated face-to-face social interaction (i.e., bottom-up) [2–4, 9, see also 56 for modern comparisons and contexts]. Consists of ~3–25 households (or under 500 people following Smith and colleagues [1] and Bodley [57]).
- **Face-block**–a small neighborhood based on community layout where households facing each other across a street form a social unit, especially as they see a lot of each other [2, 58]. These social groups facilitate the neighborhood block parties that many of us experienced in the late 20th century, where residences on the same street or block would host outdoor gatherings, creating greater bonds and social cohesion within a subgroup of a larger neighborhood community. Face-block residents may also live within a 5-minute walk from each other. Larger neighborhoods may be further divided into face-blocks.
- **Household**—a group of people living in the same residential space [59] and sharing in some (but not necessarily all) of the following activities: production, consumption, social reproduction, and physical reproduction; the basic or fundamental unit of society [60, 61]. Archaeologically, Maya households are represented by one or more houses, sometimes with auxiliary structures such as kitchens or shrines, that represent a kin-focused group [62]. They are also, and importantly, corporate groups [63]. In the Maya region, domestic and auxiliary structures may be arranged seemingly haphazardly (an “informal group”; [64]), around a small plaza or patio (a *plazuela* group [20, 65] or patio group [64]), on discrete landscape features (e.g., hilltop), or within walled lots (plots of land, or house lots, delineated by walls (*albarradas*); [2]).

Among the nested scales of social organization from households to cities, power dynamics are constantly negotiated. Such dynamics exist along a continuum from collective action or cooperation to power differentials such as patron-client relationships [66–69]. Among the modern Maya, forms of collective action are highlighted through the practice of *usk’inak’in* (“a day for a day” in Mopan Maya). *Usk’ina’kin* is a reciprocal labor practice among family and neighbors to construct houses, plant and harvest the *milpa*, and for childcare [70, 71]. However, even within the neighborhood scale, power differentials have been documented in the archaeological record. For example, at Copan, Tikal, Aguateca, Lower Dover, Uxbenká, and Ix Kuku’il, Classic Maya neighborhoods were composed of houses of varying sizes, often with a larger household (the neighborhood seat or high-status commoner [hsc] household) surrounded by smaller households [40 Table 1, 72–76, see also 77]. We interpret neighborhood seats as local centers of power, acting as patrons to their surrounding community and mediators to higher-level authorities such as district seats and above [78]. This is visible in the settlement patterns at these centers with most neighborhoods containing a visibly larger household. However, this trend is not ubiquitous across the Maya Lowlands. For example, at Caracol, the presence of neighborhood heads or a single larger household in each neighborhood has not been observed despite decades of mapping and archaeological research at the center [79]. Nonetheless, we argue that even at the neighborhood scale, different dynamics are constantly at play with collective action and forms of power and authority, such as patron-client relationships, intertwined with one another. These processes elucidate how neighborhoods formed and presumably caused the settlement patterns visible today.

### Archaeological contexts: The ancient Maya

The Maya Lowlands encompass diverse geographic regions in the neotropical forests and savannas of Mexico and Central America. Ecologically, the Lowlands include mountainous regions, rolling foothills, fertile river valleys, patchy grasslands, steep escarpments, plains, wetlands, and coastal environments. Foragers moved into the region by 12,500 BCE, adapting to the diverse ecosystems, before eventually cultivating *Zea mays* (maize) as a staple crop and building small agricultural villages [80, 81]. The earliest archaeological evidence for permanent masonry structures in the Maya Lowlands dates to approximately 1200 BCE [82–84]. By the Middle Preclassic, the Maya constructed large temples and, during the Late Preclassic, developed an incipient writing system. Dynastic divine rulership was established as early as 100 CE in some centers, where networked lords were supported by growing populations to finance their power and authority [85, 86]. Populations and centers continued to grow until 800 CE, when political disintegration swept across the Lowlands over the next 200 years [87–89]. Many of the Classic Maya centers were largely abandoned by 1000 CE. Postclassic (1000–1519 CE) Maya centers are characterized by more collective forms of governance when compared to their Classic predecessors [see 90], but exhibit large populations with evidence for social differentiation nonetheless. These centers waxed and waned throughout the Postclassic, some persisting well beyond the arrival of the Spanish in the 16th century with the final independent polity of Nojpetén (Tayasal) falling to the Spanish in 1697 CE [91].

### Lowland Maya settlement patterns

The earliest scholars of Maya settlements recognized spatial patterns in the layout and distribution of households. The initiation of settlement archaeology in the 1950s and 1960s by Willey [92, 93] drastically changed our understanding of Maya settlement patterns, shifting perceptions from JES Thompson’s [65] “vacant ceremonial centers” to large centers with clustered residential structures [64]. Our understanding of Classic Maya urban/rural settlement patterns has undergone a revolution with the advent of light detection and ranging (lidar) technology in the tropics [94–96]. While traditional approaches built the foundation for future research, often seeking to characterize “the Maya city” and landscape based on survey transects through dense tropical vegetation [27, 97–99] and, more recently, on quantitative geographic modeling [100]. The application of lidar in recent years has paved the way for a more holistic understanding of Maya settlement patterns [101]. Perhaps the most important revelation born of this research is that we should not be seeking to understand “the Maya city” but instead documenting variability along the continuum between smaller and larger Maya settlements and spatial organization of households across the landscape.

Traditional approaches tend to conceptualize Maya settlements as dispersed, low-density urbanism, like many other tropical cities [see 102–106]. Yet there are exceptions to this pattern, such as Chunchucmil and Mayapan in the Northern Lowlands [see also 39, 107, 108]. Some of the largest and most populous Maya centers exhibit lower population densities than some of their smaller counterparts. Based on settlement data, Chase and Chase [109] identified two different density trajectories that they viewed as being correlated with agricultural practices.

This variability seems born of multiple factors, including the local environmental context, social organization of corporate groups, and the degree to which local populations practiced primarily infield or outfield agriculture [110, see also 111, 112]. The inclusion of infield agricultural land and silviculture in large Maya centers has led to varied descriptive labels, including garden cities [112, 113], green cities [114], forest gardens [115, 116], and agro-urban landscapes [117]. Some have suggested that the extent of elite political power and general urban developmental processes affected variability in household clustering [38, 118–121]. While scholars have long seen clustering in the dispersed settlement patterns of the Maya region, an important question remains: just how clustered were Maya centers, and what types of social units do those clusters represent? Standardized quantitative metrics of clustering, such as average nearest neighbor analysis and kernel density analysis, prove useful for quantifying and teasing out nuance in the scale of household clustering at different Maya centers.

There is a long history of scholarship on urban form [see for example, 122–124], and Mesoamerican scholars also have a long history of engaging the topic of urbanism [125–129]. Recently, Hutson [2], building on work by earlier researchers and by ML Smith [54] that acknowledges the need for flexibility in defining “city,” proposed that cities (or what we call larger centers here) should minimally exhibit three of the following four characteristics: large size or population, high density of households, social differentiation, and specialized functions such as markets or government [see also 52]. This definition covers a broad range of Classic Maya centers. Some of the centers discussed in this study are large urban centers (e.g., Altar de Sacrificios, Caracol, Chunchucmil, Copán, El Perú-Waka’, El Pilar, Mayapan) with temples, palaces, causeways, carved stelae, ballcourts, markets, and networks of districts and neighborhoods. Many others are smaller in both extent and population but share most of the characteristics of their larger counterparts (Baking Pot, Buenavista del Cayo, Holtun, Ix Kuku’il, Las Cuevas/Monkey Tail, Los Encuentros, Lower Dover, Ojo de Agua, Pacbitun, Rosario, Uxbenká). Others are even smaller and may lack a number of characteristics that define larger Maya centers, or exhibit them at smaller scales (Aguacate Lagoon, Almon Plett, Cadena, Pescado Creek, Zibal and Kichpan Uitz). Just like modern cities, variations exist among these ancient Maya centers.

### Social groupings of the Maya

While the scale of Classic Maya settlement is now known to be far more extensive than some earlier scholars thought, with current estimates of 3–10 million people living in the Central Maya Lowlands alone (including the Guatemalan Department of Petén, the interior of Belize, and parts of the Mexican states of Campeche and Quintana Roo) during the Late Classic [see 99, 130] the socio-spatial units apparent within the settlement patterns conform fairly well with earlier models. At the smallest scale are isolated house mounds and household groups. The latter, as noted above, often take the form of a patio group [20, 64, 131]. These residential units form the basal unit of Classic Maya settlement and comprise anywhere from one to dozens of structures [62] but more often one to six structures situated around an internal patio space [61]. Patio groups are considered to have housed extended families, or, alternatively, a single nuclear family using multiple structures [132, see also 133].

The next unit up the scale is a cluster of households. As Willey [134: 255] described, “Patio-groups are… often found in clusters of from five to twelve. In such clusters, one patio-group, usually in a central location in the cluster, is larger than the others and has one mound or building that is more imposing than any of the others in that patio-group or the cluster”. Smaller households center around a single high-status household in some instances [72, 75, 76, 78], but not in others [79]. Following ME Smith [37], we interpret these entities as probable neighborhoods and see them as comparable in scale and extent to the units referred to as clusters by Ashmore [64] and Bullard [20]. Like Willey, several scholars have suggested that Classic Maya neighborhoods are composed of five to twenty households clustered together [6, 13, 17, 92, 135–137].

These ancient neighborhoods share qualities with some modern Maya multi-household corporate groups documented in ethnographic literature [72, 138–141]. Among modern Chorti Maya of western Honduras, a cluster of houses forms a *sian otot* (“many houses”), which represents a neighborhood [72]. The nested social units of the Classic Maya are described as *kúche’el* (neighborhoods and districts), *batabil* (subordinate city), and *kúuchkabal* (dominant polity) [105]. Moving forward in time from the Classic, ethnohistoric accounts describe the nested hierarchies in Maya cities, composed of smaller social communities or neighborhoods (*cah*), medium-sized *batabil*, and city-level *noh kah* in Postclassic Yucatan and northern Belize [142–144]. Similarly, in the highlands of Guatemala, among the Postclassic Quiché Maya neighborhoods were called *chinamit* or *chinamit-molab* [5, 145]. Ethnohistoric literature discusses scaled settlement patterns among the Pokom Maya, also in the highlands of Guatemala, composed of larger, medium, and small centers: *tenamit* (“town”), *kokamak* (“small population” or a hamlet), and *pajuyes* (“in the mountains” or small, scattered farms). The Pokom used *e quiz a vach tenamit* (a neighborhood within a town) and *molam* to refer to social units akin to neighborhoods [146]. Postclassic Yucatan Maya used *china* and *cuchteel* to describe smaller social units within larger political centers [10, 147, 148].

Beyond the Maya region, other Mesoamerican communities recognize nested scales of spatio-social units. Among 15^th^ century Aztec, the Nahua words *chinamitl*, *calpulli* (both small and large), and *tlaxilacalli* represent intermediate corporate groups or groups of houses (i.e., neighborhoods, districts, subordinate cities) that are a part of larger *altepetl*, (i.e., dominant city or town) [5, 77, 105, 145, 149]. Late Postclassic Tlaxcallans distinguished scaled social units as well, with larger cities composed of districts (*teccalli*) made up of neighborhoods (*tlaca*) [143]. Furthermore, at Teotihuacan, distinct ethnic neighborhoods were identified based on differences in architectural styles, isotopic analyses of buried individuals, and material culture, which revealed local (re)production of imported styles from Oaxaca and the Maya region in their respective *barrios*/neighborhoods [150, 151]. These modern, ethnohistoric, and archaeological examples of nested spatio-social units provide indigenous perspectives on how we can interpret spatial clusters of the past.

Within medium and large size centers, neighborhoods sometimes cluster into larger socio-spatial entities [152–154], which we refer to as districts. Districts may be comparable to what Bullard [20] termed a “zone” around minor ceremonial centers. Elsewhere, the terms district [3] and ward [2] have been applied to such units. Bullard [20] noted that zones were frequently focused around minor centers, which housed district seats of local administration [see 40: Table 1]. Frequently, the division between these two concepts rests upon whether such entities are “top-down” administrative entities or “bottom-up” social groupings, or social districts, although social districts and wards/administrative districts may coincide [2–4]. Settlement clustering can only tangentially be employed to assess whether units were governed in a “top-down” or “bottom-up” fashion [9, 12, 18]. Clustering may not always be easily discerned by clear spatial boundaries or unoccupied space; thus, the need for the analyses described here.

## Materials and methods

Each archaeological project collected data using pedestrian surveys, often supplemented with remotely sensed survey data. We used ESRI ArcGIS to conduct all spatial analyses. For consistency in analyses and results, we did not use other software programs (e.g., QGIS, R, GRASS).

All necessary permits were obtained for the described study, which complied with all relevant regulations. All research was conducted under permits authorized by governing agencies in Belize (Belizean Institute of Archaeology and the National Institute of Culture and History), Guatemala (Guatemalan Instituto de Antropología e Historia), Honduras (Instituto Hondureño de Antropología e Historia), and Mexico (Instituto Nacional de Antropología e Historia). Many of the archaeological projects involved in this study work with indigenous communities, engaging in community-based research [e.g., 155]. Other projects occur in national parks or reserves and work with local agencies to conduct their research [156]. Consistent with professional ethical obligations, research was presented to the public and academic communities alike and published copies of annual reports were submitted to governing institutions and communities where we conduct our research.

### Archaeological contexts and settlement data

To evaluate sociopolitical boundaries of neighborhoods, we used an ANN Analysis [11, 157] to analyze more than 24,500 households from 23 centers across the Maya Lowlands (Table 1). Data were collected by 15 archaeological projects in modern day Mexico, Guatemala, Belize, and Honduras (Fig 1). Information on each archaeological project and data is included in the supplemental text (S1 Text in S1 File). The number of households per center in these samples varies from 16 (Cadena) to 5,852 (Caracol). Because some project areas, such as the Belize River Valley, contain multiple centers, we evaluated each center individually rather than each project area. In some contexts, we ground-truthed the entirety of the center (e.g., El Perú-Waka’, Los Encuentros, Lower Dover). In other contexts, we only used validated (i.e., ground-truthed) households in the analysis (e.g., El Pilar, Pescado Creek), even if the surrounding households, and hence center, likely extended beyond the current survey boundaries (e.g., El Pilar; n = 8; Table 1). Lastly, in many contexts, we used a combination of ground-truthed and non-ground truthed data (n = 16; e.g., Baking Pot, Pacbitun). In many of these areas, scholars used lidar-derived relief visualization models including DEMs, hillshade, slope [158, 159], red relief image maps [96, 160], topographic position index [161, 162], skyview factor [163], bonemapping [164, 165], and simple local relief models to analyze and supplement areas that had undergone pedestrian survey.

**Table 1:** Descriptive data of 23 Maya centers in this study.

Center	Sub-region	Environ.	Identified residential groups	Residential group type	Study Area (km^2^)	Survey Method	Chronology	Occupational Longevity (years)	Time period used as the basis for analysis	Relative Size of Center
Aguacate Lagoon	Western Belize	Escarpments and *bajos*	37	*Plazuela*	15	PR	250–1000 CE	750	LC	Smaller
Almon Plett	Western Belize	Uplands	69	*Plazuela*	20	PR	300 BCE–500 CE	800	EC	Smaller
Altar de Sacrificios	Usumacinta/ Lower Pasion	River Valley	212	House mound groups	36	PR	950 BCE–1000 CE	1950	LC	Larger
Baking Pot	Western Belize	River Valley	1040	*Plazuela*	50	PR	900 BCE- 900 CE	1800	LC	Medium
Buenavista del Cayo	Western Belize	River Valley	292	Isolated mound and mound groups	11	PR	250–800 CE	550	LC	Medium
Cadena	Western Belize	Escarpments and *bajos*	16	*Plazuela*	11	PR	250–900 CE	650	LC	Smaller
Caracol	Vaca Plateau	Uplands	5852	*Plazuela*	200	PR	600 BCE–900 CE	1500	LC	Larger
Chunchucmil	Northern Lowlands	Plains	1410	*Plazuela*	9.3	P	400–630 CE	230	EC	Larger
Copán	SE Periphery	River Valley	884	*Plazuela*	25	P	426–820 CE	394	LC	Larger
El Perú-Waka’	Central Peten	Escarpments and *bajos*	421	Settlement Groups	29.9	PR	300 BCE–1000 CE	1300	LC	Larger
El Pilar	Western Belize	Uplands	556	Primary Residential Units	14	P	600–800 CE	200	LC	Larger
Holtun	Central Peten	Uplands	93	Patio group	7	P	1000 BCE–900 CE	1900	LC	Medium
Ix Kuku’il	Southern Belize	Uplands	215	*Plazuela*	23	PR	400–1000 CE	600	LC	Medium
Las Cuevas-Monkey Tail	Vaca Plateau	Uplands	1953	*Plazuela*	95.3	PR	700–900 CE	200	LC	Medium
Los Encuentros	Chiapas	River Valley	561	*Plazuela*	24.7	P	650–1000 CE	350	LC	Medium
Lower Dover	Western Belize	River Valley	412	*Plazuela*	15	P	500 BCE–900 CE	1400	LC	Medium
Mayapan	Northern Lowlands	Plains	4297	Households	20.6	PR	1150–1450 CE	300	PC	Larger
Ojo de Agua	Chiapas	River Valley	2004	*Plazuela*	52.2	P	500 BCE–1000 CE	1500	LC	Medium
Pacbitun	Western Belize	Uplands	1321	*Plazuela*	104	PR	300 BCE–1000 CE	1300	LC	Medium
Pescado Creek	Western Belize	Uplands	82	*Plazuela*	20	PR	250–900 CE	650	LC	Medium
Rosario	Chiapas	River Valley	2276	*Plazuela*	70.1	P	650–1000 CE	350	LC	Medium
Uxbenká	Southern Belize	Uplands	568	*Plazuela*	75	PR	200–900 CE	700	LC	Medium
Zibal and Kichpan Uitz	Western Belize	Escarpments and *bajos*	143	*Plazuela*	30	PR	600–900 CE	300	LC	Smaller

Survey method: P = pedestrian survey; R = remotely sensed survey. Time period for Analyses: EC = Early Classic; LC = Late Classic; PC = Postclassic. See S1 Fig in S1 File.

Lowland Maya residential units were documented through pedestrian survey and remote sensing. Pedestrian survey techniques vary from handheld GPS units guided by lidar data and complemented with pace-and-compass mapping to total station theodolite mapping with sub-centimeter accuracy. Pedestrian survey generally provides more accurate and detailed data than remotely sensed survey data [166] but requires both time and money, as surveyors traverse swaths of neotropical forest, identifying architectural remains along the way [167, 168]. Remotely sensed survey data has long been possible with satellite imagery [169, 170] but has gained traction with the advent of lidar for archaeological research [94]. Compared to pedestrian surveys, remotely sensed surveys are rapid, covering a larger area in a shorter amount of time, but require substantial post-survey analysis and, ideally, pedestrian verification. However, the humblest of households often remain undetectable in heavily vegetated regions [158, 168, 171]. To overcome these challenges, many archaeological projects, including those in this study (see Table 1), use multi-method approaches. Our multi-method approaches use remotely sensed imagery to guide systematic pedestrian surveys and ground-truthing, as well as to make estimates of missed structures to aid in both standardized and accurate mapping and societal reconstructions.

Our dataset contains case studies from diverse geographical locations, of variable size and density of settlements, and with localized occupational histories. Understanding patterned variability in this dataset thus provides a holistic evaluation of ancient Maya settlement patterns. Within our dataset, centers are located across a variety of environments including upland regions (e.g., Ix Kuku’il), alluvial valleys (e.g., Copán), the edges of escarpments (e.g., El Perú-Waka’), and flat shrublands (e.g., Chunchucmil). Centers ranged from densely occupied cities with nearly 900 structures/km^2^, as in El Perú-Waka’s central core [172: Table 5.2, 173], to low-density landscapes with 15 structures/km^2^, as at Ix Kuku’il [78: Table 3]. Notably, across the highest and lowest density settlements and those in-between, the average number of structures per household / residential group is between 3 and 4 (see S1 Text in S1 File).

**Fig 2:**
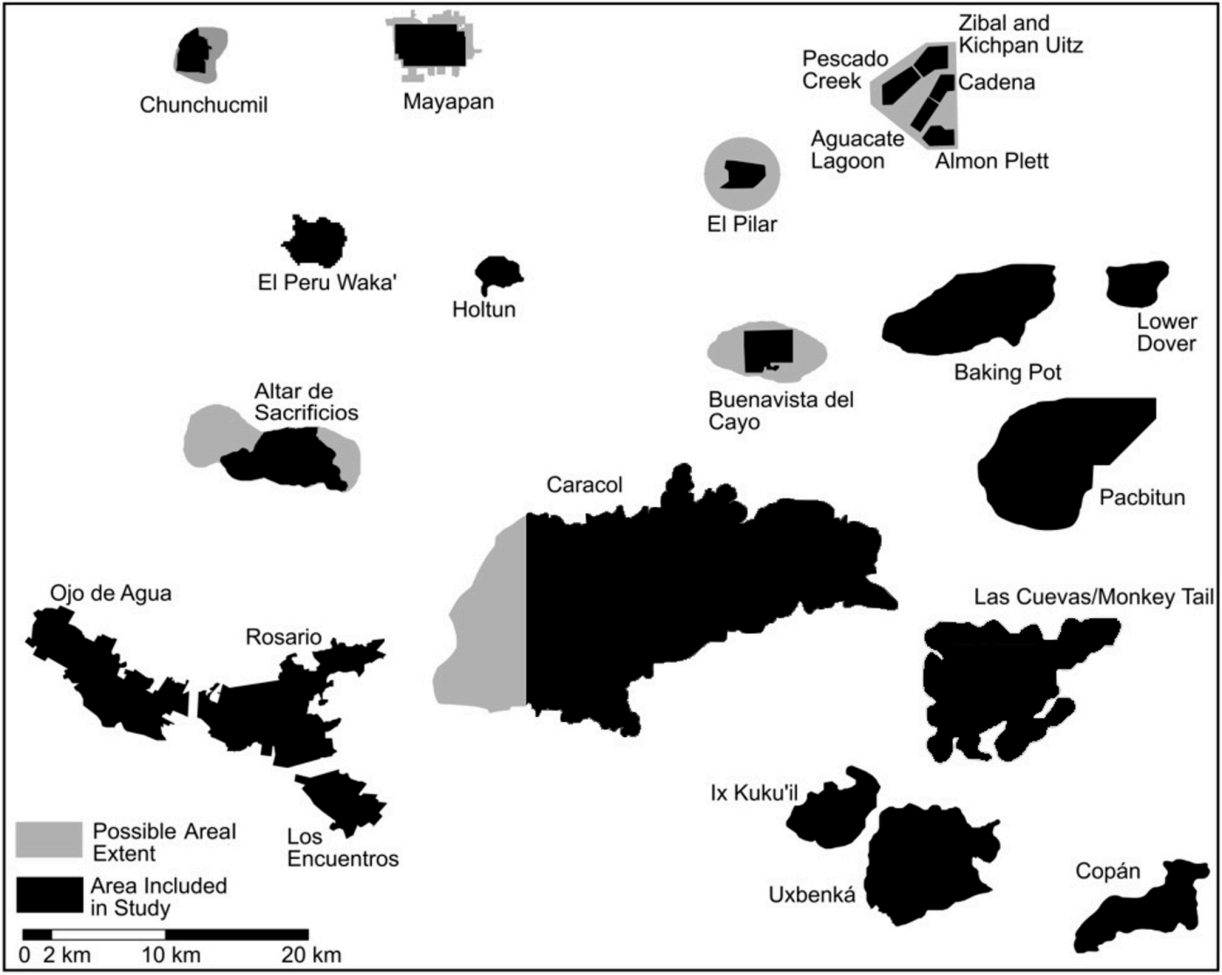
Scaled map of the different centers included in this study. Black areas are the extent that was used in the study and gray areas represent the extent of settlement not included in this study, at times because of modern borders. (Image by JPW).

In total, we use settlement data from 23 Maya centers. All centers had some degree of pedestrian survey, and 13 are supplemented with previously obtained lidar data [96, 158, 162, 168, 174–176] (see S1 File). Centers range in size (Fig 2) from geographically small but densely occupied centers, such as Chunchucmil, with an estimated 2,500 residential groups within 15 km^2^ (of which 1410 households in a 9.3 km^2^ were used in this study), through small, rural centers such as Pescado Creek, which contains 82 residential groups across 20 km^2^, to the largest center, Caracol, which exhibits an expansive settlement covering at least 200 km^2^ and presumably containing some 9,000 households [9]. The diversity in our dataset highlights the utility of this method and our findings, regardless of the size, scale, and use of survey method (pedestrian only vs. combination pedestrian and remotely sensed).

**Fig 3:**
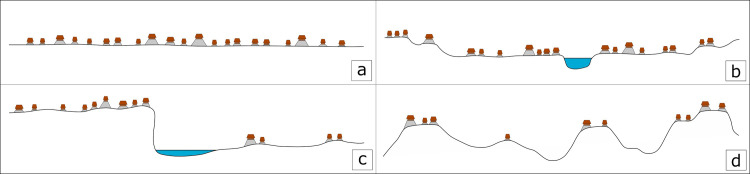
Schematic profiles of different environmental settings. Schematic profiles show how environment may impact the distribution of ancient Maya households. The four broad environmental settings used in this paper include flat plains (a), river valleys (b), escarpments and *bajos* (c), and uplands (d). (Image by AET).

The centers included in this study are located in a variety of environmental settings that can be distilled to four basic categories: flat plains, river valleys, escarpments and *bajos*, and uplands (Fig 3). Uplands include montane regions and their foothills as well as rolling hills where household settlement is often directly associated with major rivers. River valleys are characterized by large rivers with alluvial floodplains often surrounded by uplands along the edges of the valley. Escarpments and *bajos* are a series of upland ridges with steep sides dropping down to swampy *bajos* that may be seasonally inundated with water. Finally, the plains in the Yucatan Peninsula of the northern Lowlands are characterized by less topographic variation, shallow soils above limestone bedrock, and a drier climate. In addition to facilitating the identification of potential neighborhoods, the large dataset provides insights into settlement trends among the environmental zones of the neotropical forests of the Maya Lowlands.

Most of the Maya centers we discuss date in their final mapped forms to the Classic Period (250–900 CE) and often toward the end of that period, although some emerged earlier in the Middle Preclassic Period (800–400 BCE, e.g., Pacbitun [177]). Palimpsests of occupation occurred as these ancient centers waxed and waned through time, but within each dataset the settlement patterns commonly reflect a snapshot of the final peak period of occupation. With the exception of Almon Plett, Chunchucmil (Early Classic), and Mayapan (Postclassic), the settlement system of the other 20 centers reflects the Late Classic between 550–900 CE (Table 1), a period during which regional settlement densities reached their apogee.

### Average Nearest Neighbor tool

To assess the degree to which households are clustered, we ran an ANN analysis for each of the 23 centers. The ANN tool in ArcGIS calculates the Observed Mean Distance (OMD), which is the average distance from each point–in this case the center point of a residential group–to its closest neighboring point. The ANN tool also calculates the Expected Mean Distance (EMD) for an identical number of points scattered randomly across the same space. EMD is the average distance from each of these random points to its nearest neighbor. OMD divided by the EMD comprises the ANN Ratio. If the ratio is 1, the distribution of residential groups is random. An ANN ratio of less than 1 (e.g., when OMD is less than EMD) suggests clustering because points are nearer to each other than they would be if randomly scattered. An ANN ratio greater than 1 suggests dispersion, as points are farther away from each other than they would be if randomly scattered. For each ANN ratio, ArcGIS calculates the probability (converted from a z-score) that the observed scatter of points could have been sampled from a completely random universe of points. The lower the probability, the more likely that a clustered pattern (ANN ratio < 1) is indeed clustered and the more likely that a dispersed pattern (ANN ratio > 1) is indeed dispersed.

The probability value can often be more illuminating than how far the ANN ratio deviates from 1 since even small deviations away from 1 can be significant (e.g., low p-value) when sample sizes are large. In sum, a statistically significant degree of clustering suggests that residential compounds can be aggregated spatially into discrete groups. KD analysis helps pinpoint such spatial clusters and, if they contain between five and twenty households [92], they can be interpreted as a type of neighborhood. A statistically significant degree of dispersion suggests that most residences were evenly spread across the terrain. Household clusters can exist even in a dispersed pattern, and these clusters may be considered neighborhoods, but it may also be the case that neighborhoods exist without clustering (i.e., that neighborhoods are not as spatially salient–an issue in modern cities, see [56]–or that a different type of social organization prevailed [178]). ANN ratios and their associated p-values allow for comparison between centers, essentially leveling the playing field regarding the patterns of distribution regardless of size of center or number of inputs. The ANN statistic of “clustered/dispersed” should not be conflated with the notion of a “household cluster” (see above) or a “cluster of households” as the ANN statistic represents the distribution of all households across the landscape.

However, the ANN tool functions as a snapshot of time or a single phase in the occupational sequences of these ancient communities. By nature, the tool is not diachronic and requires that all inputs are contemporaneous. For long-lived centers with well-dated settlement contexts, multiple ANN analyses could be conducted to assess how settlement patterns change over time (for an example of how others have assessed population based on household occupation over time, see [179]). For this study, we use the ANN as a snapshot of a single time period, specifically when the center reached its apogee, typically during the Late/Terminal Classic (see above and Table 1). For example, at Uxbenká and Ix Kuku’il, more than 30% of the documented residential groups were dated using multi-proxy approaches (ceramic typologies and radiocarbon dating, see [40]). Of the dated residential groups, more than 95% were occupied during the Late Classic [180]. For centers with palimpsests of occupation or residences known to be abandoned and reoccupied, only residential groups occupied during the same periods should be analyzed.

In our analysis, each center was analyzed individually in ESRI ArcMap (not ArcPro, which has modified inputs for the ANN tool). We use the center points of household groups as the input feature class, run the analysis using Euclidean distance (as opposed to Manhattan distance), and check the box to generate a report of the finding. All analysts then recorded the OMD, Nearest Neighbor Ratio, z-score, p-value, and if the results were clustered or dispersed.

### Effects of survey boundaries

Boundary effects (defined as limits to archaeological data recording and/or processing) are important when considering the inputs for the ANN analysis. The inputs must represent a meaningful socio-spatial unit and should be an adequate sample of the overall population for statistical measures. As with any archaeological study, households may have been missed during pedestrian survey due to time constraints [168], dense foliage, or invisible platforms / “vacant terrain” where low-lying house foundations may be obscured by natural soil processes [181, 182]. Similarly, low-lying households not situated on raised platforms may be missed in lidar surveys [158, 171]. Many of us overcame these issues by combining pedestrian survey data with remotely identified households using satellite imagery or lidar data. We note that similar trends are present among the centers that use pedestrian survey data and those supplemented with remotely sensed survey data. In some cases, excavation data revealed settlement not visible in either data class.

However, three of us (A.S.Z. Chase, Hutson, and Thompson) ran the analyses on our data at multiple scales, arriving at similar results. For example, Hutson ran the ANN on the 9.3 km^2^ dataset of Chunchucmil and an extended area of Chunchucmil covering 15 km^2^ with patchy survey (which is why it was not included in the final analysis) and found similar patterns in the ANN ratio for both. A.S.Z. Chase ran the ANN analysis on 5,852 *plazuela* groups in a 200 km^2^ area of Caracol (in modern Belize) and 7,709 *plazuela* groups from part of greater Caracol, which extends to 300 km^2^, and found similar trends of clustered settlement patterns, OMD (99.5 m vs. 95.5 m), and ANN ratios (0.84 vs 0.77). Thompson ran the ANN on two datasets of Uxbenká, which used pedestrian and lidar-based survey data: a smaller area with 308 *plazuela* groups and a larger area with 568 *plazuela* groups. The ANN results were nearly identical with ANN ratios of 0.79 and 0.80, respectively. Previously, using only pedestrian survey data of 105 households from Uxbenká, the ANN ratio was 0.83 [11]. These three examples highlight that, assuming the survey area is large, the sample adequately covers the area, and large patches of land did not go unsurveyed, the effect of the survey boundary, while important, has little effect on the ANN outcome. This holds true when using pedestrian data or a combination of pedestrian and remotely surveyed data, smaller or larger samples sizes, and environmental setting. While many of these centers likely have additional settlement extending beyond the boundaries used in this analysis that may not be included in this sample due to modern agricultural practices, patchy pedestrian survey, dense foliage, etcetera (see S1 Table in S1 File), we believe that all the centers except Cadena have sufficient survey coverage to characterize settlement clustering within their particular context.

### Kernel density tool and neighborhood identification

In addition to the ANN tool, we used the KD tool to visually identify the clustering of households. KD predicts the density of input points–here, residential groups–by calculating the density of input features, in this case households, around each output cell using the smoothing parameter and can be used to calculate the density of housing [26]. KD is better suited for population-based density analyses, compared to simple point density analyses which assumes the weight of the point occurs in a single location. Rather, the kernel density function spreads the values over an area using a Gaussian distribution and as such it "provides a more realistic model of the population distribution" ([183]: pg 344; see also [184]). Weighted features can be added, but we did not weight residential groups by size, number of structures, or population estimates because some contributors have those data for all residential groups, while others do not. Not including weighted features standardized our inter-case study samples as much as possible given the parameters. Likewise, in ArcGIS Pro, barriers can be added, such as rivers, roads, or topography which may delineate households in real life. These avenues could be investigated in future analyses.

We analyze each center individually using the KD analysis. The center points of household groups that were used in the ANN analyses were also used in the KD analysis. We standardize the input parameters, ensuring that additional variables are not affecting the KD outputs (Population Field: None; Output cells: 1m; Search Radius: Observed Mean Distance from the ANN; Area: Sq_km; Output Value: Densities; Method: Planar). The resulting KD raster is then reclassified into 32 classes using natural breaks (jenks). The raster was adjusted from the default color ramp to the “temperature” color ramp (white to dark red) and reduced to 50% transparency while making the lowest reclassified class transparent so the terrain was visible as a layer beneath the KD results. The standardized KD rasters using the quantitative inputs, such as the OMD from the ANN, plus qualitative assessments of the landscape were used to manually digitize potential neighborhoods.

Others have used the KD for neighborhood identification in other archaeological contexts. In the Maya region, KD was previously used at Baking Pot [185], El Pilar [157], Ceibal [186], Mayapan [10], and in southern Belize [11]. In many ways, the approach is more beneficial for examining patterns in an individual context in a more perceptive manner rather than comparing different contexts due to variation in the KD; one cannot standardize or normalize the KD outputs among the 23 datasets given the underlying algorithm. We overcome this issue by reporting the highest KD value for each center and comparing the ANN ratios and qualitative outputs of the KD tool.

Each project identified potential neighborhoods from their study region based on the ANN/KD results and *a priori* information such as the presence of ancient paths, roads (*sacbeob*), topography, and streams/rivers. High density KD values encompassing clusters of households for each center (which vary, see above) usually included between 3 to 25 households, following trends that were noted by Mayanists decades ago [20, 64], although such patterning was not ubiquitous. In our identification of neighborhoods, we stipulated that not every household had to be included in a neighborhood. Analysts digitized neighborhood boundaries based on their observations of the KD patterns, following the densest areas on the raster and delineating neighborhoods in places where the KD output bottlenecked or did not touch, topography and natural features, and anthropogenic features such as roads, reservoirs, or other services [see 187] which may act as natural boundaries between past neighborhoods (e.g., [2] in the Maya region; [45] in the Andes), just as in neighborhoods today (but see also [56]).

## Results

### Average Nearest Neighbor results and neighborhood identification

The ANN analysis provides three key components: the OMD between households; the nearest neighbor (NN) ratio or the OMD divided by the EMD, which indicates a clustered, random, or dispersed pattern; and a p-value, which indicates the degree to which a clustered or dispersed pattern is statistically significant. Across the 23 centers in our sample, 21 exhibit a clustered pattern according to the logic of the ANN (Table 2); that is, the households are clustered into small social units within the larger dataset. The two exceptions are Chunchucmil and Mayapan, two densely occupied centers. While Mayapan is technically clustered according to the ANN output, its NN ratio of 0.99, high p-value, and the KD output makes it appear dispersed, and neighborhoods were difficult to distinguish using visual spatial clustering alone. (Previous work by Hare [10] used a k-means cluster analysis to identify neighborhoods). Another outlier, Cadena, is a small center with only 16 households. Many of these centers are clustered in a statistically significant fashion based on the p-value. According to individual analysts, the standardized ANN/KD method identified potential neighborhoods at a majority of the centers analyzed (Aguacate Lagoon, Almon Plett, Altar de Sacrificios, Baking Pot, Buenavista del Cayo, Cadena, El Perú-Waka’, Ix Kuku’il, Los Encuentros, Lower Dover, Ojo de Agua, Pescado Creek, Rosario, Uxbenká, Zibal and Kichpan Uitz); it was found to be only moderately useful at two centers (Copán, Las Cuevas-Monkey Tail), and was not found to be as useful for some of the larger centers (Caracol, Chunchucmil, El Pilar, and Mayapan).

**Table 2:** ANN / KD results.

Center	Settlement Density of study region (groups/km^2^)	Observed Mean Distance (m)	Nearest Neighbor Ratio	z-score	p-value	Clustered or Dispersed	KD value	# of residential groups per neighborhood
Aguacate Lagoon	2.5	216.35	0.84	-1.85	0.064	Clustered	23.6	5–15
Almon Plett	3.5	153.09	0.68	-5.06	<0.001	Clustered	167.7	5–15
Altar de Sacrificios	5.9	106.76	0.44	-15.03	<0.001	Clustered	359.7	5–15
Baking Pot	20.8	68.70	0.63	-23.07	<0.001	Clustered	661.0	5–15
Buenavista del Cayo	26.5	113.00	0.79	-7.65	<0.001	Clustered	333.0	5–15
*Cadena*	*1*.*5*	*175*.*33*	*1*.*21*	*1*.*50*	*0*.*133*	*Random*	*29*.*6*	*5–15*
Caracol	29.3	95.50	0.77	-33.12	<0.001	Clustered	374.8	5–25*
Chunchucmil	151.6	50.40	1.09	6.16	<0.001	Dispersed	956.0	20–40
Copán	35.4	61.59	0.57	-24.81	<0.001	Clustered	1119.6	5–15
El Perú-Waka’	14.1	124.00	0.93	-2.72	0.007	Clustered	193.7	5–15
El Pilar	39.7	78.30	0.94	-2.69	0.007	Clustered	627.3	5–15
Holtun	13.3	98.90	0.79	-3.95	<0.001	Clustered	337.6	5–15
Ix Kuku’il	9.3	163.00	0.88	-3.28	0.001	Clustered	115.5	5–15
Las Cuevas-Monkey Tail	20.5	99.90	0.82	-15.37	<0.001	Clustered	380.1	5–15
Los Encuentros	22.7	37.11	0.32	-30.71	<0.001	Clustered	2472.7	15–30
Lower Dover	27.5	63.40	0.66	-13.25	<0.001	Clustered	647.0	5–15
Mayapan	208.6	34.00	0.99	-1.73	0.084	Clustered	4423.4	20–40
Ojo de Agua	38.4	31.90	0.36	-55.08	<0.001	Clustered	3895.3	15–30
Pacbitun	12.7	113.40	0.68	-21.77	0.001	Clustered	1033.2	5–15
Pescado Creek	4.1	158.84	0.83	-3.00	0.003	Clustered	100.7	5–15
Rosario	32.5	38.39	0.36	-58.89	<0.001	Clustered	3211.2	15–30
Uxbenká	7.6	154.00	0.80	-9.11	<0.001	Clustered	193.4	5–15
Zibal and Kichpan Uitz	4.8	133.48	0.77	-3.91	<0.001	Clustered	250.8	5–15

Average Nearest Neighbor tool, Kernel Density, and neighborhood identification results. The ANN analysis of Cadena (italicized) had a high p-value and, therefore, was not used in any further analyses. *please see [9: 306–310].

The ANN/KD method for neighborhood identification based on the clustering of households seems more useful with lower densities and more dispersed settlement patterns, where social organization involved rural farmsteads (e.g., Alto Magdalena, [179, 188]) not nucleated and gridded settlements (e.g., Indus Valley, [189]). Other locations where this method may prove useful is the Early Dynastic period (2500–2334 BCE) of Mesopotamia where Truex [190] noted clusters of houses, West Africa including Kirikongo and Jenne-Jeno (250 BCE– 1400 CE), where spatially discrete clusters mounds were documented [191], or ancient Andean communities, where potentially clustered compounds were reported dating to the Initial Period to Early Horizon (1700–150 BCE) at Caylán [192].

OMDs vary from approximately 32 m to 216 m, with a mean and median of 101 m and 99 m, respectively. Notably, the highest OMD value (Aguacate Lagoon) is greater than two standard deviations above the mean. This is likely due to the geographic region (escarpments and *bajos*) but also possibly due to gaps in the survey data (which, unlike survey boundaries, could impact these results), due in part to modern agricultural practices in the region. The remaining 22 centers fall within two standard deviations of the mean. Likewise, Cadena, which has the smallest sample size in our study with 16 households has a high p-value (Table 2) and, therefore, was not included in further analyses. Cadenda’s dispersed pattern with distantly spaced households (OMD: 175 m) may represent rural farmsteads like those documented by Peterson and Drennan [179] at Alto Magdalena (Columbia) and is akin to the Pokom Maya concept of *pajuyes* (“in the mountains”; [146]) for distantly spaced houses in rural communities.

Potential neighborhoods were identified by the visual clustering of households from the KD map derived from the OMD of the ANN test. Each contributor manually digitized or automated the digitization (using a raster reclassification of the KD results to auto-generate shapefiles) of their own neighborhoods based on the high-density areas on the KD map, incorporating their knowledge of the local landscape for variables that may encourage or deter interactions between households. Such variables may include rivers and steep valleys, ancient roads, or other architectural features such as walls (*albarradas)*. The neighborhood delineations offered by contributors combine both quantitative assessments (ANN and KD) with more qualitative observations of the landscape, resulting in a flexible model of neighborhood designation while using the same method. This method can be applied to case studies around the world to identify social units of varying scales.

More than half of the data analysts (9/15) identified potential neighborhoods, to some degree, through these geospatial methods. We found that if the ANN ratio was < 0.80, neighborhoods of spatially clustered houses were more distinguishable. When the ANN ratio ranged from 0.81–0.90, household clusters were more difficult to discern, but were often distinguishable with the inclusion of *a priori* information such as landscape features, shared household characteristics, etc. Neighborhoods were more difficult to identify based on household clusters when the ANN ratio was > 0.90. Specifically, at Chunchucmil and Mayapan in the northern Lowlands ANNs were almost 1 (Mayapan) or higher (Chunchucmil) and neighborhoods were not discernible using this method. Previously, neighborhoods at Chunchucmil were identified based on shared artifact characteristics, movement, and focal nodes that would have encouraged social interaction [2, 10], while a different spatial analysis, a k-means cluster analysis and nearest neighbor hierarchical clustering, were used to identify neighborhoods at Mayapan (see [10]). The ANN reflects the distribution of households, and in our case studies the threshold for using the ANN for neighborhood identification lies between 0.81 and 0.90, but also depends on local variables.

### Differential densities

Ancient and modern communities alike vary in settlement density, from rural farmlands to densely packed cities. The density of communities varies based on the area and number of households and is quantified in our results of the OMD, ANN ratio, and KD value. In our sample, we found variations in the OMD between household centroids and ANN ratios. The OMD is the average spacing between any household and its closest neighbor. While not directly correlated with settlement density, it provides insight into the distribution of households across these varied landscapes and provides a basis for future research on settlement density and population, which is beyond the scope of this paper.

The KD value (Table 1) reflects the highest density at each center. The KD values range from 23.6 (Aguacate Lagoon) to over 4,000 (Mayapan), highlighting the variation in Maya centers. Due to the heterogeneous nature of Classic Maya centers, we did not standardize the density maps because local differences in density would have been obscured by applying the highest density to each center. Rather, each analyst used the highest KD values for their dataset, permitting them to see how household clustering occurs in their study area. Generally, there is an inverse relationship between the OMD and the KD value–centers with low OMD have higher densities; the KD value stored in the raster output file changes drastically based on search distance, with smaller distances providing higher maximum values [26]. Similarly, centers in upland and escarpment environments have lower KD values than centers in river valleys, suggesting that higher density centers form in areas with more room to expand and infill a single area.

**Fig 4:**
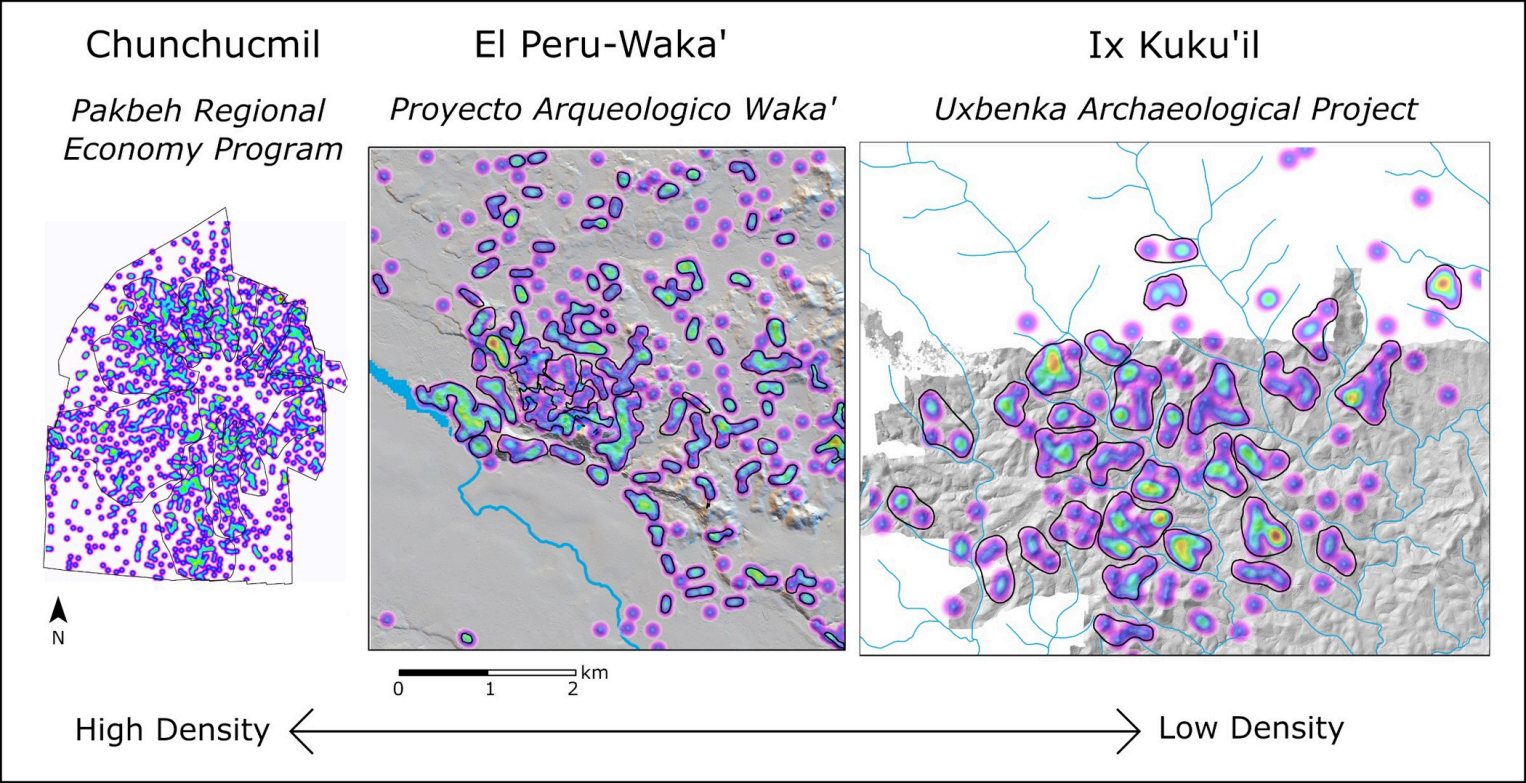
Variations in settlement density at Chunchucmil, El Peru-Waka’, and Ix Kuku’il. All maps are at the same scale, but KD values are based on local household distributions (see Table 2). (Maps by SRH (Chunchucmil), DBM (El Peru-Waka’) and AET [Ix Kuku’il]).

Finally, we note that while some centers exhibit dispersed settlement, such as Ix Kuku’il, others are densely occupied (Chunchucmil), and others still have variation in density across the landscape, such as El Peru-Waka’ (Fig 4). El Perú-Waka’ has a large swath of densely spaced residential settlement surrounding the monumental civic-ceremonial core, but density drops off as one moves out of the core into the near periphery and drops off even more in the far periphery; patches of more densely occupied areas are present in the near periphery, highlighting the heterogeneous composition of El Perú-Waka’ [172]. Other centers with variation in settlement density across the landscape include Caracol, Pacbitun, and Copán. It was easier to distinguish household clusters, or neighborhoods, in areas of lower density such as in El Perú-Waka’s far periphery than in areas of high density near the core. In other cases, the dispersal of households away from the monumental architecture may be due to infield agriculture [110, 193] that could have been co-managed/maintained by neighborhood residents and/or by intermediate elites. Centers with extremely high densities, like the Rosario Valley centers and Mayapan, may have relied heavily on outfield agricultural practices.

## Discussion

The identification of potential neighborhoods through the clustering of households builds towards deeper, more holistic understandings of past social organization, human-environment dynamics, and settlement patterns among the ancient Maya. The smaller social units in the vast communities in which we live today create a sense of social solidarity and connect us to others both geographically and socially. In these residential clusters, the Classic Maya may have similarly developed a deeper sense of community based on shared experiences [51], but this requires further investigations of artifact classes, architectural patterns and spaces for aggregation, burial and caching practices, and communities of practice within neighborhoods of a single center [e.g., 9, 12, 15, 19, 186, 194, 195]. Neighborhoods provide access to corporate and kin-held property, labor, resources, and identities, all of which underpin everyday life. Among modern Maya communities, smaller social units engage in shared activities that include childcare, building houses, and farming [70, 196]. Here we discuss how our results of household clustering and neighborhood identification through smaller social units relate to the heterogeneous nature of Maya centers.

### Environmental considerations

This study highlights the variability in terrain of neotropical forests in Central America. From the rivers cutting through mountainous regions in Honduras, to the uplands of the Maya Mountains and foothills in Belize, to the broken and undulating terrain of the Petén karst plateau, and the flat plains with low forest of the northern Yucatan Peninsula, the landscape is anything but homogenous [see also 197]. Like their forested environments, Maya centers vary in their compositional diversity as highlighted in our discussions of settlement patterns and neighborhood groupings.

The microenvironments within the Maya Lowlands affect not only the location of households across a single landscape [198] but also the average distance between residences and the clustering of households. We binned our datasets into four major environmental categories: flat plains, river valleys, uplands, and escarpments and *bajos*. In the two plains centers, Chunchucmil and Mayapan, households are more evenly dispersed but have less distance between them due to higher settlement density (Table 3). The ANN/KD method for neighborhood identification did not work well at either of the Maya centers in the plains in our sample. According to the logic of the ANN analysis, these two centers with the most nucleated settlement pattern are characterized as dispersed (Table 2 shows Mayapan as clustered because the analysis includes settlement beyond its walls; Mayapan’s settlement is dispersed within its walls). At Chunchucmil there is some degree of sprawl, which we define as settlement with peri-urban density of between 60 and 150 structures per km^2^ [96]. Hutson and colleagues [199] identified fingers of peri-urban settlement extending to the east and southwest of the center but found rural settlement densities to be lower (<60 structures per km^2^) to the west, north, and southeast of the site. At Mayapan, the clustering is at the level of the political center within the city wall rather than the neighborhoods, as is visible on the settlement map (S17 Fig in S1 File). While small neighborhoods could, at times, be teased out, there was often overlap, and the presence of overtly large clusters made it difficult to discern neighborhood groupings. Rather, the contributors working at both of these settlements relied on previous archaeological evidence for neighborhood identification [see 2, 10, 35].

**Table 3:** Descriptive statistics for environmental variability, geographic region, and urban scale.

	ANN Ratio	ANN Ratio Min	ANN Ratio Max	Average OMD (m)	Average OMD (m) Min	Average OMD (m) Max	Sample Size
Environment							
Plains	1.04	0.99	1.09	42.2	34	50.4	2
River Valley	0.51	0.32	0.79	65.11	31.9	113	8
Uplands	0.8	0.68	0.94	123.88	78.3	163	9
Escarpment	0.85	0.77	0.93	157.94	124	216.35	3
Geographic Region							
Northern Lowlands	1.04	0.99	1.09	42.2	34	50.4	2
Chiapas	0.35	0.32	0.36	35.8	31.9	38.39	3
Lower Pasión	0.44	-	-	106.76	-	-	1
Central Petén	0.86	0.79	0.93	111.5	98.9	124	2
Western Belize	0.76	0.63	0.94	122.1	63.4	216.35	9
Vaca Plateau	0.79	0.77	0.82	97.7	95.5	99.9	2
Southern Belize	0.84	0.8	0.88	158.5	154	163	2
Southeastern Periphery	0.57	-	-	61.59	-	-	1
Urban Scale							
Large Center	0.82	0.44	1.09	78.65	34	124	7
Medium Center	0.66	0.32	0.88	95.04	31.9	163	12
Small Center	0.76	0.68	0.84	167.64	133.48	216.35	3

Observed Mean Distance abbreviated to OMD and Average Nearest Neighbor abbreviated to ANN.

**Fig 5:**
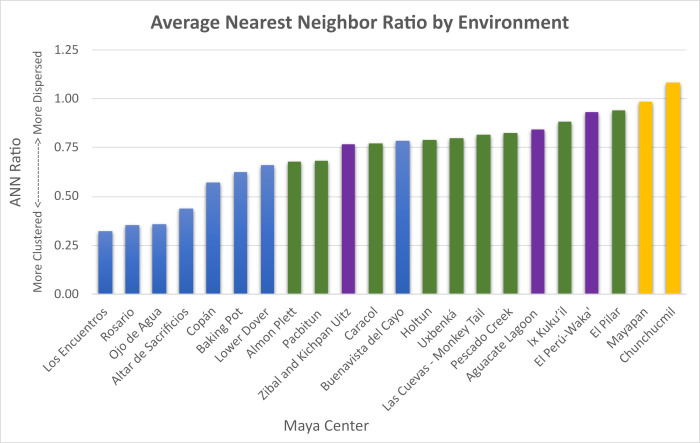
Average Nearest Neighbor (ANN) ratio by environmental setting of Maya centers. Plains = gold; River Valley = blue; Uplands = green; Escarpment = purple. (Image by AET).

**Fig 6:**
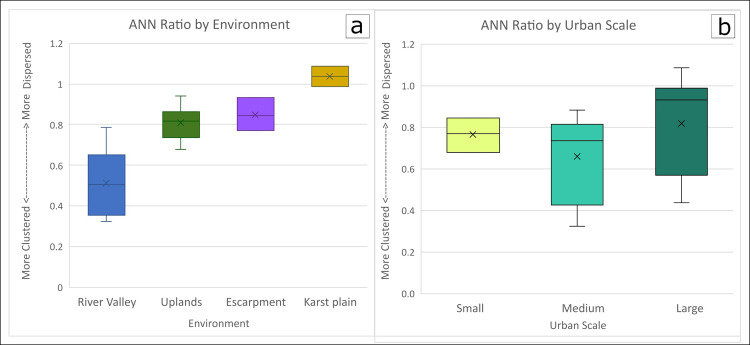
Box and whisker plots. Plots show the Average Nearest Neighbor (ANN) ratio by environmental setting (a) and urban scale (b) of Maya centers. (Image by AET).

River valleys generally saw greater degrees of household clustering and smaller distances between household groups than the upland and escarpment areas. Household spacing is usually less than 100 m with an average of 65 m. The average distance is similar to that noted between houses in the Valley of Oaxaca (Mexico) and the Western Liao Valley (China), which ranged from 50 to 70 m [179]. River valley centers tend to be clustered with ANN values ranging from 0.32 to 0.79 with an average of 0.51. Most contributors were able to use the ANN/KD to identify potential neighborhoods. The three centers with the lowest OMD–Los Encuentros, Rosario, and Ojo de Agua–cluster together with low OMDs (approximately 35 m) and ANN ratios (0.34) but high KD values compared to the other centers in our study (Table 2). These centers are located in Chiapas and exhibit a different settlement pattern from most parts of the Maya lowlands. This pattern sees more households clustered towards the center and fewer households dispersed across the landscape, resulting in distinct groupings of houses. This variability may be due to the fact that the region was far from the heartland of the Maya Lowlands and, while the centers were Maya, the associated hinterlands were likely inhabited by Mixe-Zoquean speakers [200]. Overall, most centers in river valleys exhibit greater trends toward clustering based on lower ANN ratios (Figs 5 and 6). In denser centers, like Copán, neighborhood identification with this method remained a challenge; however, in other centers, like Altar de Sacrificios, neighborhoods can be more easily posited based on geospatial analyses alone.

Upland regions are characterized by discrete hilltops, usually resulting in greater spacing between residential units compared to the plains and river valley settlement systems (Table 2). Nonetheless, many of the upland centers exhibit clustered settlement patterns. The average distance between upland households is double that of river valley centers and more than three times that of centers located in the plains. Within upland centers, households often cluster into discrete spatial groupings, although at centers with higher ANN ratios and less discretely clustered households, neighborhoods were more difficult to discern than at upland centers with lower ANN ratios. This is likely partially due to topography with extended kin groups residing on long ridges. However, at Uxbenká it was noted that several hilltops lacked archaeological remains, resulting in a buffer zone of no settlement, suggesting that settlement decision-making and neighborhood formation was an active part of settlement selection [40].

Escarpment regions exhibit the greatest distance between residential units, with an average of 158 m and ANN ratios ranging from 0.77 to 0.93 (Table 3). Generally, centers on escarpments were clearly clustered and their neighborhoods were discernible. At El Perú-Waka’, the variation in household density made it easier to identify neighborhoods in the hinterlands and more difficult to tease apart neighborhoods near the city center, where neighborhoods were also slightly larger. The El Perú-Waka’ case study illustrates the disparities in household density within single political units, highlighting different forms of neighborhoods that may have existed in the past.

Some of the patterning in residential clustering no doubt speaks to the degree to which a population was reliant on infield versus outfield agricultural practices [110, 112, 201, 202]. This patterning varied between and within centers, and to a large extent the clustering and distances between residences was dependent on this infield versus outfield distinction, leading to two distinct trajectories of settlement size and resulting population densities [109]. On one end of the spectrum, some large nucleated centers like Mayapan were likely entirely reliant on imported food or outfield agriculture. Likewise, the residents of Chunchucmil were likely highly reliant on imported food and outfield agriculture, but they did use house gardens [203]. On the other end of the spectrum lies Caracol, which, despite its size, had space between households for infield cultivation and would have been agriculturally self-sufficient [204]. However, the urban system and landscape observed at Late Classic Caracol bears the effects of path dependence and early commitment to infield agriculture through terracing [205]. While clear patterns emerge among centers, variability also exists within individual settlements; residences situated in the cores and fringes of larger centers were reliant on infield and outfield cultivation to varying degrees (see Fig 4). The intertwined dynamics between residential clustering, environmental zone, and agricultural practices are important topics for future investigation.

### Geographic variability

Human-environment dynamics were fundamental to the formation of ancient communities and our findings allude to how both environment and geographic regional differences articulate with trends in settlement density, social organization, and the formation of neighborhoods. The centers used in our study are dispersed across eight geographic regions: the Northern Lowlands (n = 2), Chiapas (n = 3), the Lower Pasion (n = 1), the Central Petén (n = 2), Western Belize (n = 9), the Vaca Plateau (n = 2), Southern Belize (n = 2), and the Southeastern Periphery (n = 1) (Table 3). Most of our geographic regions have small sample sizes ranging from one to three centers, many of which are in the same environmental category (uplands, plains, river valley). Nonetheless, these data provide insights into geographic variations in settlement patterns that can be further explored in the future.

As noted above, the two centers in the northern Yucatan exhibit similar settlement patterns of closely spaced houses with low OMDs and high ANN ratios. Visually examining the settlement maps of Mayapan (S17 Fig in S1 File) and Chunchucmil (S8 Fig in S1 File), reveals that areas of higher density are visible, but discrete clusters prove more difficult to discern. Likewise, the three centers from Chiapas have distinct settlement patterns. Here, the patterns show distinct clusters of households with large spaces between them (S15, S18, and S20 Figs in S1 File). Caracol and Las Cuevas/Monkey Tail are on the Vaca Plateau and have similar ANN ratios and OMDs; both are in upland environments. However, just south of the Vaca Plateau on the other side of the Maya Mountain is Southern Belize, where the ANN ratios and OMDs of Uxbenká and Ix Kuku’il are similar to each other, but greater than those observed in the Vaca Plateau even though all four centers in these two geographic regions are in upland environments (Table 3), suggesting that simple upland-lowland distinctions fail to predict settlement types. The landscape of western Belize is varied, with river valleys, uplands, and escarpments, resulting in a range of ANN ratios and OMDs. The variability in the terrain no doubt impacts the results of the ANN analysis, where ANN ratios range from 0.63 to 0.94 with OMDs between households averaging 122 m but ranging from 63 m at Lower Dover to more than 200 m at Aguacate Lagoon.

### Settlement scale and center size

Maya centers vary in their size (i.e., spatial extent of their populations) and density (i.e., clustering of households). While these exist on a continuum, for ease of comparison, we grouped the centers in our case study into three broad categories–small, medium, and large centers–based on the size of the civic-ceremonial architecture in the core, extent of settlement/population, and the political power of their ruling elites based on hieroglyphic inscriptions and the presence and number of architectural features associated with power and authority, such as E-groups and ballcourts. The differences in settlement scale in the archaeological sample presented here parallel the Pokom Maya concepts of *tenamit* (“town”), *kokamak* (“small population” or a hamlet), and *pajuyes* (“in the mountains” or small, scattered farms) [146], which provides insights into Maya concepts of social spaces. Within the Classic Maya centers presented here, nested social units are present as *kúche’el* or *cah* (neighborhoods and districts), which likely represented varying forms of cooperation from collective action to coercive cooperation [40].

**Fig 7:**
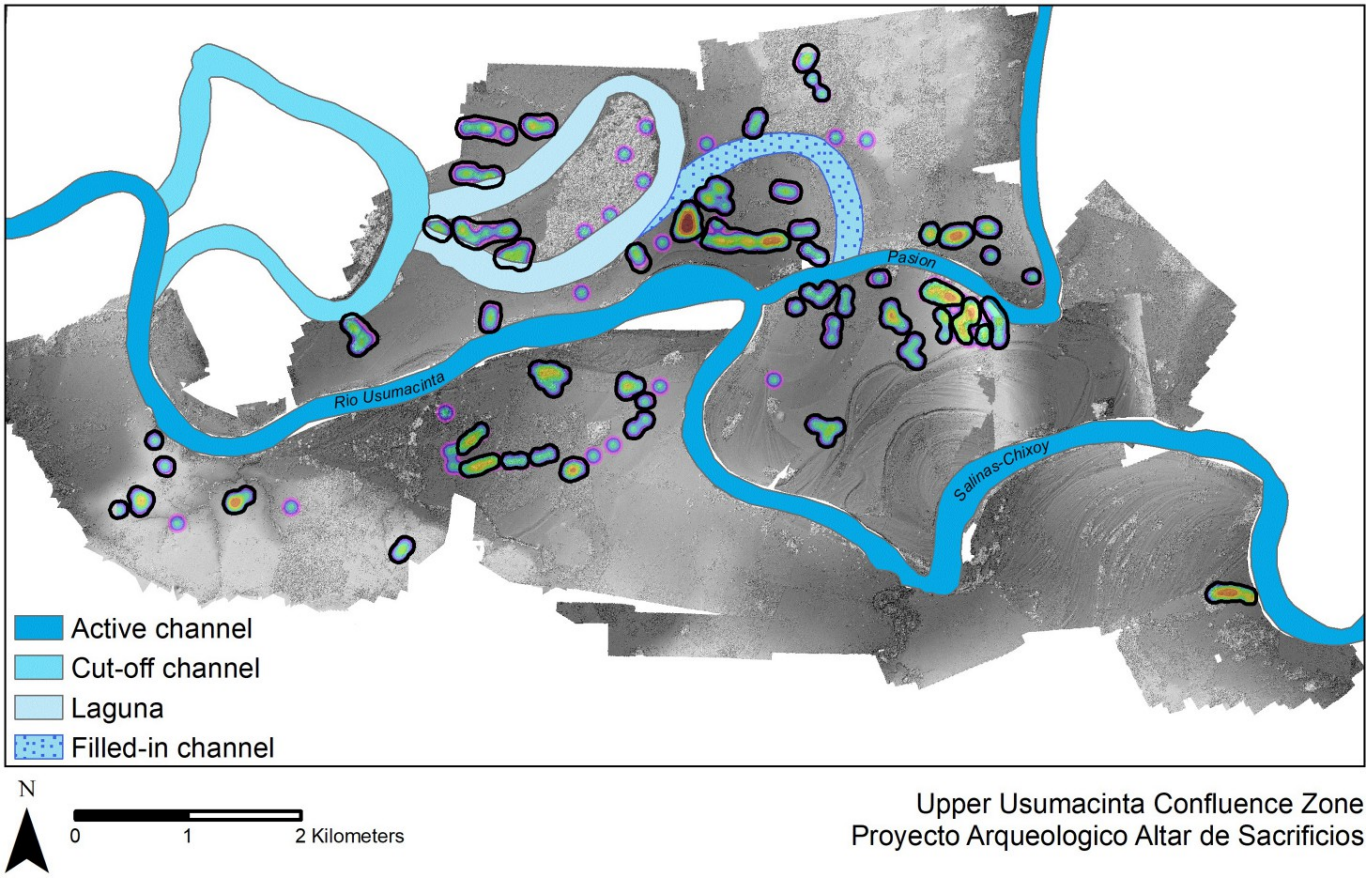
Altar de sacrificios. Close up of Altar de Sacrificios showing how changes in the river coursing affects settlement identification. (Map by JM; basemap made by Andrés G. Mejía-Ramon).

The largest of the centers, Caracol, is an outlier from the rest of the sample due to its large spatial extent (200 km^2^) and population of 100,000 residents. Like the size differences between modern NYC, which has a population of 8.8 million people, and other large cities in the US such as Los Angeles and Chicago, with populations of nearly 4 million and 2.7 million respectively, the largest ancient Maya centers vary in spatial extent and population. However, compared to medium and small centers, they exhibit greater spatial extent, population, and political trappings as evidenced by the archaeological record. Some of the larger centers contain seemingly small populations based on pedestrian survey but substantial civic ceremonial core architecture, alluding to large-scale social mixing and energetics required to construct such monumental spaces [206–208]. This discrepancy likely results from the dearth of survey data in the surrounding landscape deflating population estimates (e.g., Altar de Sacrificios, which was affected by the changing course of the Rio Chixoy (Fig 7)); “vacant terrain” settlement also skews such results [e.g., 181]. We recognized different clustering patterns across the larger centers, with more nucleated clustering in the middle, near monumental architecture, and smaller clusters of households dispersed across the landscape forming neighborhoods or face-blocks.

Medium centers have varying degrees of nucleation, with some exhibiting dense settlement near their civic ceremonial cores (e.g., Baking Pot, Pacbitun) and others following a low-density urbanism plan [*sensu* 102], where smaller households surround larger households thought to be the homes of intermediate elites at Lower Dover [76] and Uxbenká [75, 78]. The trends in the smallest settlements in our sample are harder to distinguish in part due to their small populations and small sample size. Generally, they do not exhibit the variation in clustering across the landscape as visible in the medium and larger centers, but reflect a rural landscape with low settlement density.

**Fig 8:**
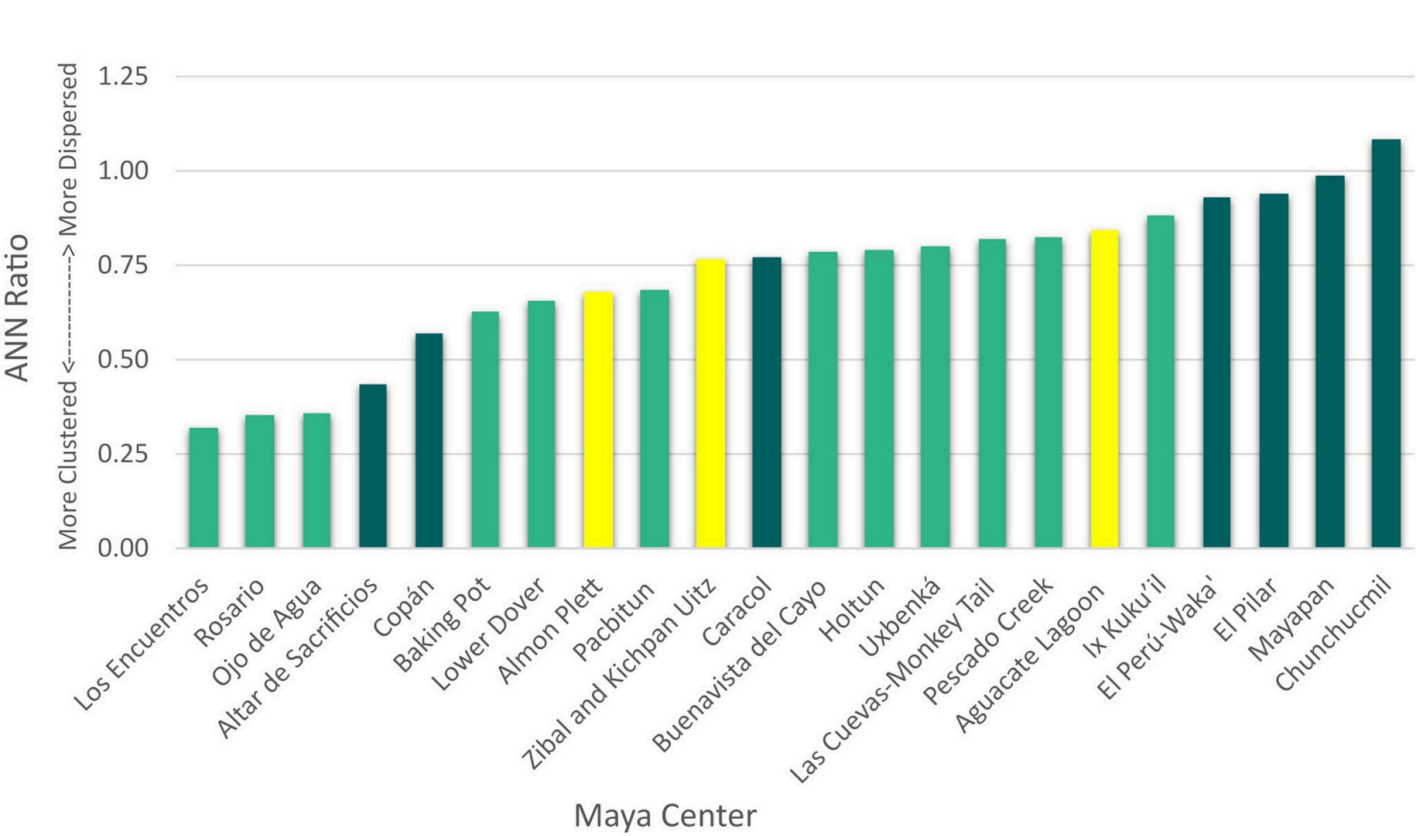
Average Nearest Neighbor (ANN) ratio by urban scale. Smaller = light green; Medium = turquoise; Larger = teal. (Image by AET).

To some degree, the ANN ratios correspond to the size of ancient Maya centers (Figs 6 and 8). The largest centers tend to have higher ANN ratios, with an average of 0.82, compared to medium size centers (mean ANN ratio: 0.64) (Table 3). The two large centers located in river valleys (Copán and Altar de Sacrificios) are an exception to this trend, with ANN ratios of closer to 0.50, while most of the other large centers in our sample (Mayapan, Chunchucmil, El Pilar, and El Perú-Waka’) have higher ANN ratios above 0.93. Yet, the largest center in the sample, Caracol, exhibits an ANN ratio of 0.77, matching values found in smaller centers; this ratio may result from the inclusion of sustained agricultural space into its landscape, in contrast to the more nucleated large centers analyzed here (Mayapan, Chunchucmil). Ongoing analyses of the spatial relationships between residential units and “vacant terrain” at El Pilar, however, suggest sustainable *milpa*-cycle agriculture was integrated into the urban landscape of this large center as well. Using a combination of ethnographic data on traditional agricultural methods and lidar-derived slope models, the El Pilar team found that spaces between residences within the city at appropriate grades for growing crops could provide food for the entire estimated population, with additional surplus production achievable through increased labor inputs [209]. This suggests another factor may have influenced the relative dispersion of residences at El Pilar as compared to Caracol and the more nucleated large centers.

In small centers, households group into discrete clusters (neighborhoods) that are spaced out across the landscape, with ANN ratios between 0.68 and 0.84, averaging 0.76. The slightly deflated ANN ratios of small centers and many medium size centers make it easier to identify clusters of houses, or neighborhoods. The heterogeneous layout of the large centers, including densely occupied places like Mayapan and Chunchucmil, or the variation in settlement density at El Perú-Waka’ and Caracol [172: Table 5.2, 174: Fig 11] resulted in difficulties identifying neighborhoods, particularly near civic ceremonial cores, through KD spatial analyses alone; at Caracol, different spatial analyses for neighborhood identification were carried out successfully [9]. In some of the centers in our analyses, neighborhoods were easier to distinguish in hinterland regions, removed from the civic ceremonial architecture. Nonetheless, we note a general trend in center size and ANN, suggesting that settlement density, population growth, farming practices, and geopolitical clout may affect patterns of social organization like neighborhoods.

### Different types of neighborhoods

Among 15 of the centers in our case study, the KD analysis often picked up smaller clusters composed of 5–15 households (Table 1), similar to the concept of face-blocks, but well within expected neighborhood sizes in most parts of the Maya world ([2], also see above). Such examples include Aguacate Lagoon, Almon Plett, Altar de Sacrificios, Baking Pot, Buenavista del Cayo, Caracol (far periphery), Copán, El Perú-Waka’, El Pilar, Ix Kuku’il, Las Cuevas/Monkey Tail, Pacbitun, Pescado Creek, Uxbenká, and Zibal and Kichpan Uitz. However, other centers, like the Rosario centers in Chiapas, have more households in their clusters (15–30 households). Caracol’s neighborhoods contain between 5–25 households [9]. The densely nucleated centers of Chunchucmil and Mayapan both have larger clusters of 20–40 households. This is also true of the larger clusters present in the vicinity of many civic-ceremonial centers where settlement nucleates (e.g., Baking Pot, El Perú-Waka’, Pacbitun). In some cases, the KD has delineated units comparable in scale–but not necessarily in the same layouts–to what some of us have called districts elsewhere [2, 12, 39, 40, 180, 210].

Social communities of varying sizes exist in past and present communities. These multi-scalar communities are nested within each other, resulting in complex social relationships with individuals interacting within and between multiple, interlocking communities. Teasing apart variability between nested units in different contexts in the past has proven difficult; while the types of comparative analysis conducted here clarify how a single method provides quantitative answers to household clustering, there still remains striking degrees of variability between centers. This method provides one way to assess neighborhood types; in the future, additional spatial analyses and analysis of material culture or architecture will provide further insights into the diversity of neighborhood types and composition.

### Broadening perspectives

Neighborhoods and smaller social communities are well-documented in global contexts past and present [5, 8, 41, 58]. These smaller social units were likely initially formed through cooperation with many residents working together through processes of collective action (e.g., *allyus* in the Andes, or *usk’ina’kin* in the Maya region). The observation that households within larger political centers spatially cluster into smaller social units has been documented around the world using both qualitative observations and quantitative analyses. Drennan and Peterson [179], documented shifts in the clustering of households among numerous culture groups through time. Likewise, the clustering of households to form neighborhoods has been noted by many others [190–192].

Our study presents a methodological approach that could be applied to any spatio-temporal context to identify potential neighborhoods. Based on our analyses, this approach works well in low-density cities and perhaps would also perform well in the Khmer empire of southeast Asia [211], West Africa near Jenne-Jeno and Kirikongo [191], or Postclassic Aztec communities outside of Tenochtitlan such as Cuexcomate [105]. Our approach will likely prove less fruitful in regions with densely settled, multi-room buildings, such as the Great Houses of the US SW, Indus Valley settlements like Harappa, or Roman cities like Ostia [212], where all households are, to some degree, interconnected, and in rural dispersed farmstead communities such as the Alto Magdalena in Colombia.

## Concluding remarks

This paper presents quantitative spatial analyses of 23 Maya centers from 15 collaborating archaeological projects across the Maya Lowlands. This scholarship builds on nearly a century of settlement survey in the Maya region (reviewed in Hutson [2]) and on recent multi-project, lidar-specific collaborations, including the Pacunam Lidar Initiative (PLI) consortium composed of nine projects [96] and the West-Central Belize Lidar Consortium comprising six archaeological projects [101, 213], to understand past human behaviors through settlement patterns and spatial analyses. We welcome other Mayanists to join our collaborative group of scholars seeking to study past human behaviors through standardized approaches.

Large groups of people often subdivide into smaller social units. Among modern cities, neighborhoods, boroughs, and wards delineate such social units and are often characterized by location, shared architectural styles, and identities. Neighborhoods existed in the past as well, although identifying them through the ephemeral remains of the archeological record remains a challenge. Clustering of residences among the Maya has been noted for nearly a century [27], and here we applied two methods, ANN and KD, to identify household clusters akin to neighborhoods among 23 Maya centers with comparable quantitative metrics. These centers ranged in size from small and medium centers to expansive and densely populated locales. Regardless of size or geographic location, neighborhoods were identified in most of our sample, albeit at times through additional methods [2, 9, 72]. The PLI assessed household clustering and density at the urban scale, identifying rural, peri-urban, and urban areas [96]; we advance this research by assessing intra-community residential clustering to understand the heterogeneous nature of Maya centers at different urban scales and within a dynamic human-environment relationship.

Like modern cities, ancient centers do not fit into a one-size-fits-all model, nor does a single spatial analysis work for every place. However, our findings provide quantitative metrics for evaluating variations in household clusters of the past. Centers situated in river valleys tend to have higher degrees of clustering (lower ANN ratios), making it easier to identify household clusters, while centers in the plains of the northern lowlands have lower degrees of clustering (higher ANN ratios). The diverse nature of large centers results in challenges for using the ANN / KD method at this scale of settlement; it is easier to identify household clusters among medium size and small centers. This study is relevant not only to archaeologists studying past human behaviors, but also to geographers assessing the environmental variability of a landscape, to sociologists studying social solidarity, and to urban planners evaluating neighborhood growth. Highlighting the diverse nature of ancient communities, this study builds a foundation for future collaborative endeavors using spatial analyses to assess past human behaviors.

## Declarations

### Field research–archaeological research permits

Belize: Research in Belize was conducted under the Belize Institute of Archaeology (IoA) permit numbers: #IA/R/39/15/19/03 to TGP (Pacbitun); #IA/H/2/1/14(08) to KMP (Uxbenká and Ix Kuku’il); #IA/H/2/1/19(05) to AF (El Pilar); #IA/R/44 to JMorris (Aguacate Lagoon, Almon Plett, Cadena, Pescado Creek, Zibal, Kichpan Witz); WR/46/20/ 02 to HM (Las Cuevas); #IA/H/2/1/13(17) to JRY and #IA/H/2/1/13(18) to MKB (Buenavista del Cayo), #IA/H/3/1/15(17) to JJA (Lower Dover, Baking Pot), and #IA/H/3/1/2020(03) to AFC and DZC (Caracol).

Guatemala: Research in Guatemala was conducted under the Instituto de Antropología e Historia, Ministerio de Cultura y Deportes and Guatemalan Instituto de Antropología e Historia (IDAEH) permit numbers: DAJ-285-2018 to DMB and JC Pérez (El Perú-Waka’); 08–2017, 12–2018 to JMunson (Altar de Sacrificios); 01–2018 to AF (El Pilar); DAJ-220-2015 to BK and MC (Holtun)

Honduras: Research in Honduras was conducted under the Instituto Hondureño de Antropología e Historia (IHAH) permits to HRR for the MayaArch3D Project (Copan).

Mexico: Research in Mexico was conducted under the Consejo de Arqueología, Instituto Nacional de Antropología e Historia (INAH) permit numbers: C.A. 401-36/800, C.A. 401-36/0232, C.A. 401-36/0461, C.A. 401-36/0472 to B. Dahlin and TA, C.A.401-36/1067 to SRH and TA (Chunchucmil); 401.1S.3-2019/780 to JMunson (Altar de Sacrificios); 401.B(4)19.2013/36/0189 to TSH (Mayapan).

## Supporting information

S1 File This includes settlement maps (S1 Fig) and kernel density maps (S2 Fig) for the 23 Maya centers used in this study, as well as maps for each center (S3– S21 Figs). Additional supplemental files include boundary effects for each center (S1 Table) and brief descriptions of centers analyzed by each archaeological project (S1 Text).
(DOCX)
