# Supplementary material for: Ancient Lowland Maya neighborhoods: Average Nearest Neighbor analysis and kernel density models, environments, and urban scale
Source: PLoS One. 2022 Nov 2;17(11):e0275916. doi: 10.1371/journal.pone.0275916 (PMC9629605; doi:10.1371/journal.pone.0275916)
Supplement: S1 File — Additional supplemental files include boundary effects for each center (S1 Table) and brief descriptions of centers analyzed by each archaeological project (S1 Text). (DOCX) [file pone.0275916.s001.docx]

**Supplemental Files for**

**Ancient Lowland Maya Neighborhoods: Average Nearest Neighbor Analysis and Kernel Density Models, Environments, and Urban Scale**

Thompson, Amy E., John P. Walden, Adrian S.Z. Chase, Scott R. Hutson, Damien B. Marken, Bernadette Cap, Eric C. Fries, M. Rodrigo Guzman Piedrasanta, Timothy S. Hare, Sherman W. Horn III, George J. Micheletti, Shane M. Montgomery, Jessica Munson, Heather Richards-Rissetto, Kyle Shaw-Müller, Traci Ardren, Jaime J. Awe, M. Kathryn Brown, Michael Callaghan, Claire E. Ebert, Anabel Ford, Rafael A. Guerra, Julie A. Hoggarth, Brigitte Kovacevich, John M. Morris, Holley Moyes, Terry G. Powis, Jason Yaeger, Brett A. Houk, Keith M. Prufer, Arlen F. Chase, and Diane Z. Chase

**Supplemental Figures**


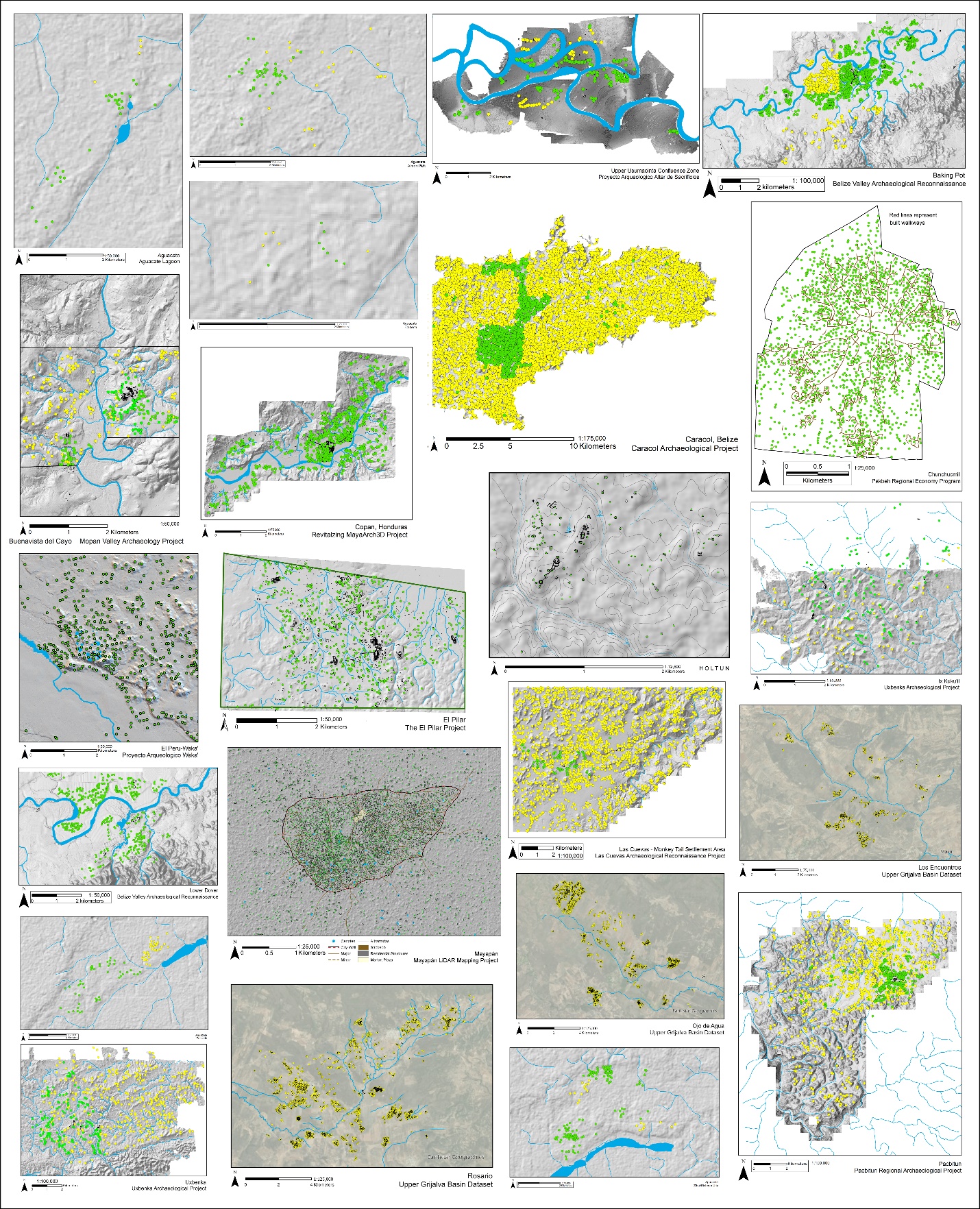


**S1 Fig**. **Settlement maps** for each of the 23 centers used in this study. Green points represent households identified during pedestrian survey and yellow points represent households identified through remote survey. Individual scales for each center. Basemaps for centers use lidar-derived hillshade models or ESRI basemaps or were produced from ASTER DEMs v.3. The basemap for Holtun (map by MRGP) produced from an ASTER DEM version 2. Basemaps for Aguacate Lagoon, Almon Plett, Cadena, Pescado Creek, and Ziabl (maps by ECF) were derived from a freely available ASTER GDEM version 3 (NASA). Basemaps for Rosario, Ojo de Agua, and Los Encuentros (maps by KSM) consist of 0.6m resolution imagery that is the intellectual property of Esri, Maxar, and Earthstar Geographics and is used herein under license. Copyright 2020 Esri, Maxar, and Earthstar Geographics and their licensors. All rights reserved.


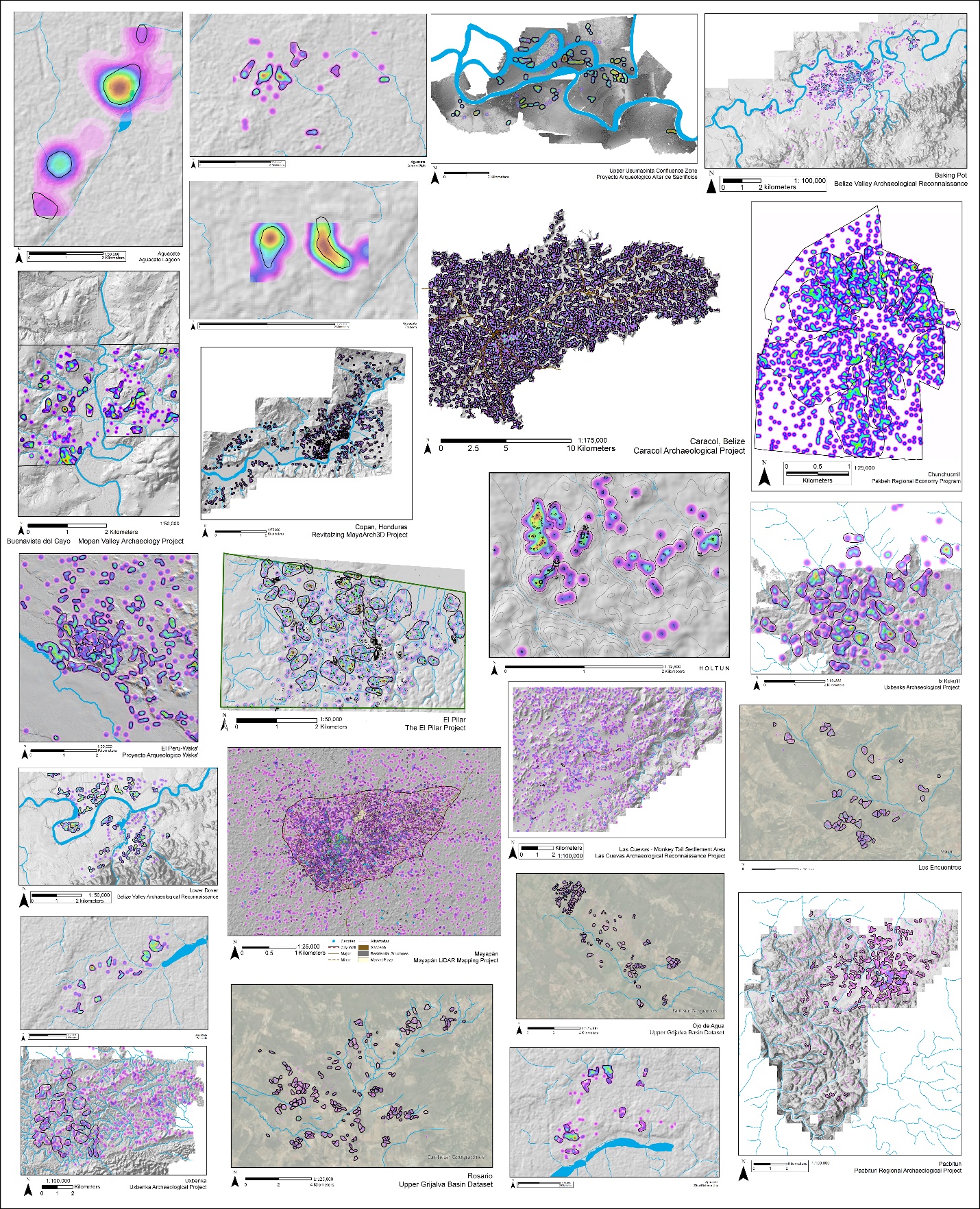


**S2 Fig**. **Kernel Density maps** showing the clustering of households at each of the 23 centers in this study. KD values are specific to each map. Individual scales for each center. Basemaps for centers use lidar-derived hillshade models or ESRI basemaps or were produced from ASTER DEMs. The basemap for Holtun (map by MRGP) produced from an ASTER DEM version 2. Basemaps for Aguacate Lagoon, Almon Plett, Cadena, Pescado Creek, and Ziabl (maps by ECF) were derived from a freely available ASTER GDEM version 3 (NASA). Basemaps for Rosario, Ojo de Agua, and Los Encuentros (maps by KSM) consist of 0.6m resolution imagery that is the intellectual property of Esri, Maxar, and Earthstar Geographics and is used herein under license. Copyright 2020 Esri, Maxar, and Earthstar Geographics and their licensors. All rights reserved.

**
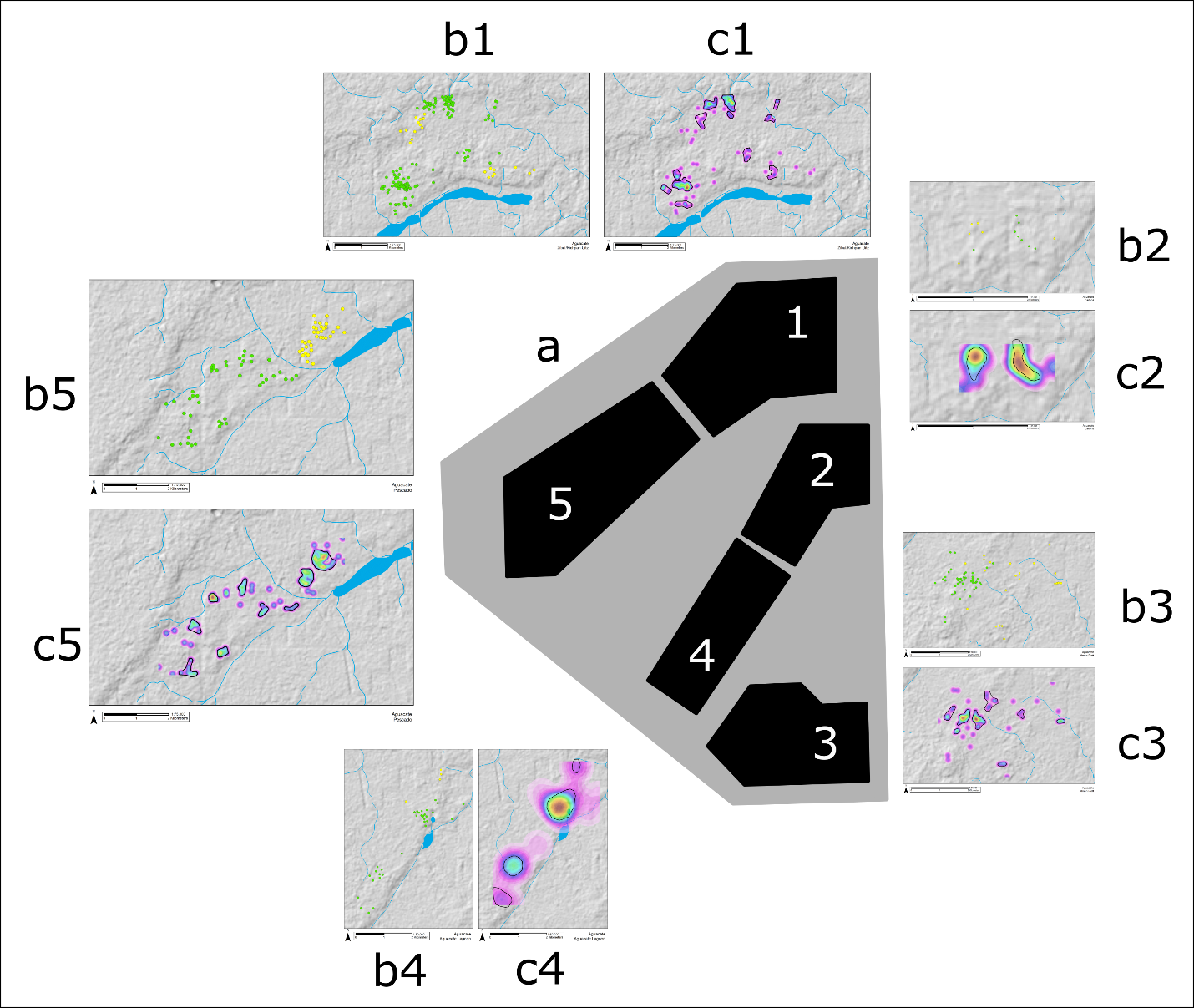
**

**S3 Fig**. **Aguacate Lagoon, Almon Plett, Cadena, Pescado Creek, and Zibal and Kichpan Uitz maps**. Scaled map (see Fig 2), settlement map (see S1 Fig), and kernel density map (see S2 Fig) of Aguacate Lagoon, Almon Plett, Cadena, Pescado Creek, and Zibal and Kichpan Uitz. Basemaps derived from a freely available ASTER GDEM version 3 (NASA). Maps by ECF; figure by AET.

**
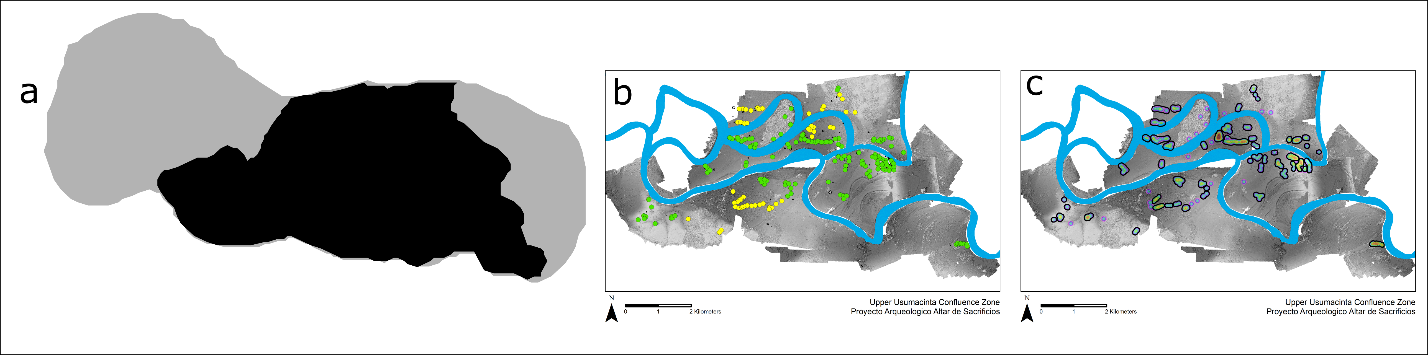
**

**S4 Fig**. **Altar de Sacrificios maps**. Scaled map (see Fig 2), settlement map (see S1 Fig), and kernel density map (see S2 Fig) of Altar de Sacrificios. Basemap made by Andrés G. Mejía-Ramon. Maps by JM; figure by AET.

**
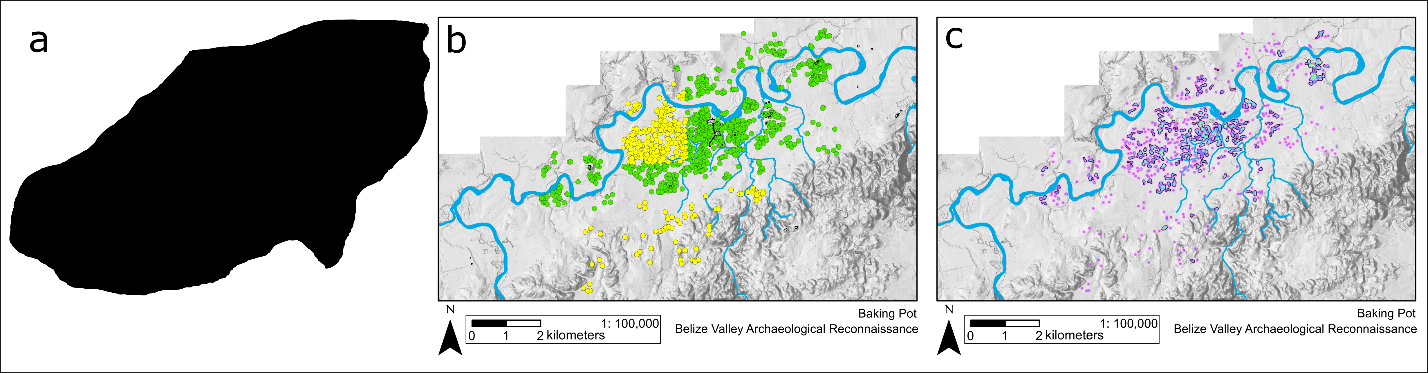
**

**S5 Fig**. **Baking Pot maps**. Scaled map (see Fig 2), settlement map (see S1 Fig), and kernel density map (see S2 Fig) of Baking Pot. Maps by JPW; figure by AET.

**
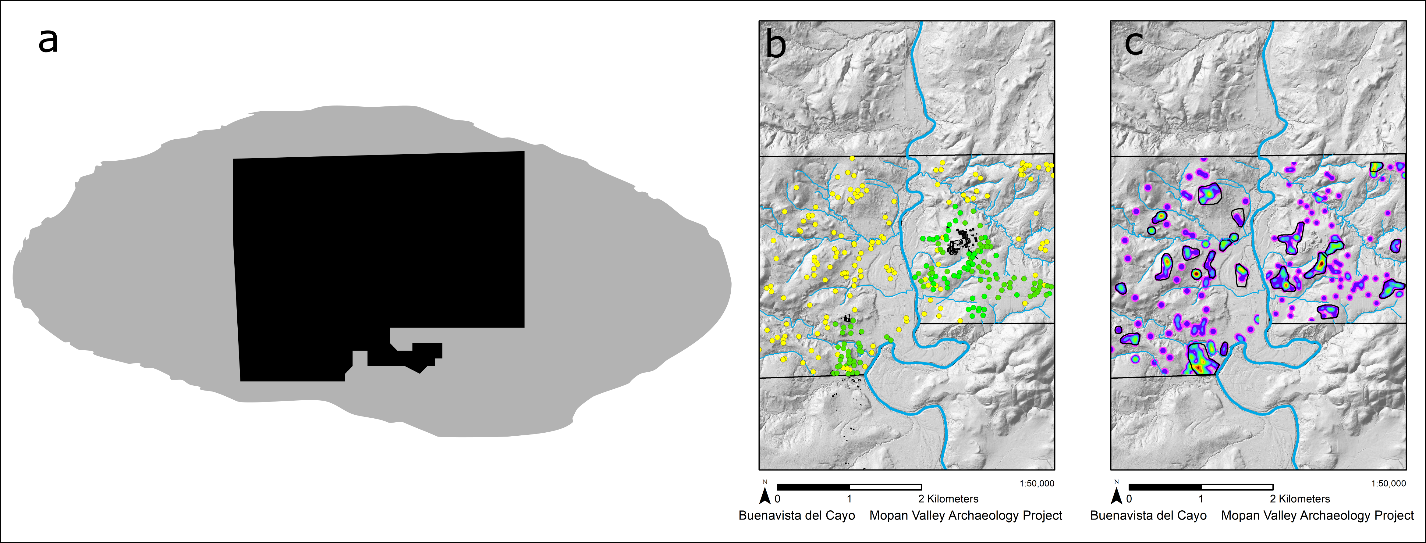
**

**S6 Fig**. **Buenavista del Cayo maps**. Scaled map (see Fig 2), settlement map (see S1 Fig), and kernel density map (see S2 Fig) of Buenavista del Cayo. Maps by BC; figure by AET.


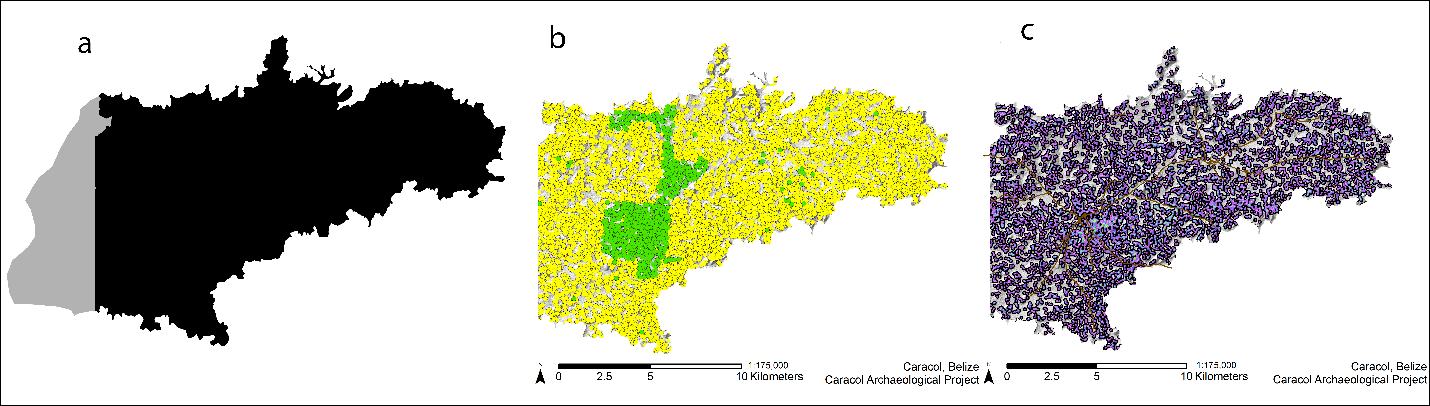
**S7 Fig**. **Caracol maps**. Scaled map (see Fig 2), settlement map (see S1 Fig), and kernel density map (see S2 Fig) of Caracol. Maps by ASZC; figure by AET.

**
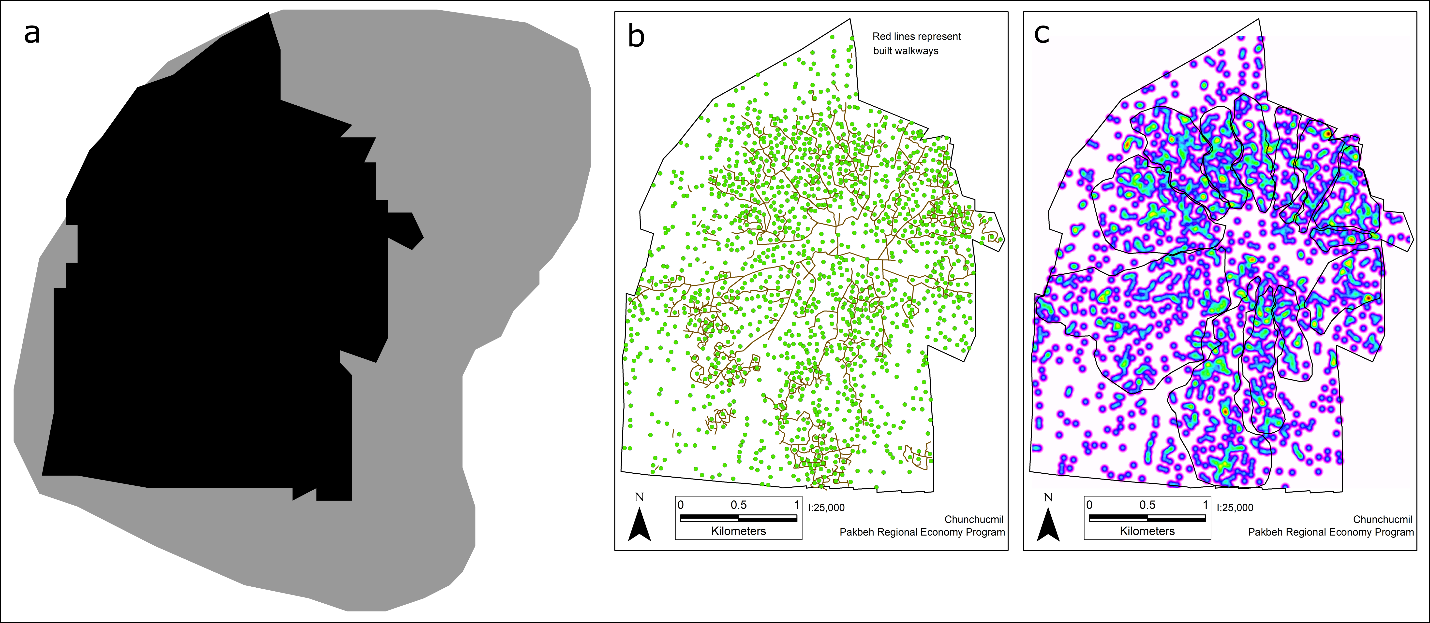
**

**S8 Fig**. **Chunchumil maps**. Scaled map (see Fig 2), settlement map (see S1 Fig), and kernel density map (see S2 Fig) of Chunchucmil. Maps by SRH; figure by AET.

**
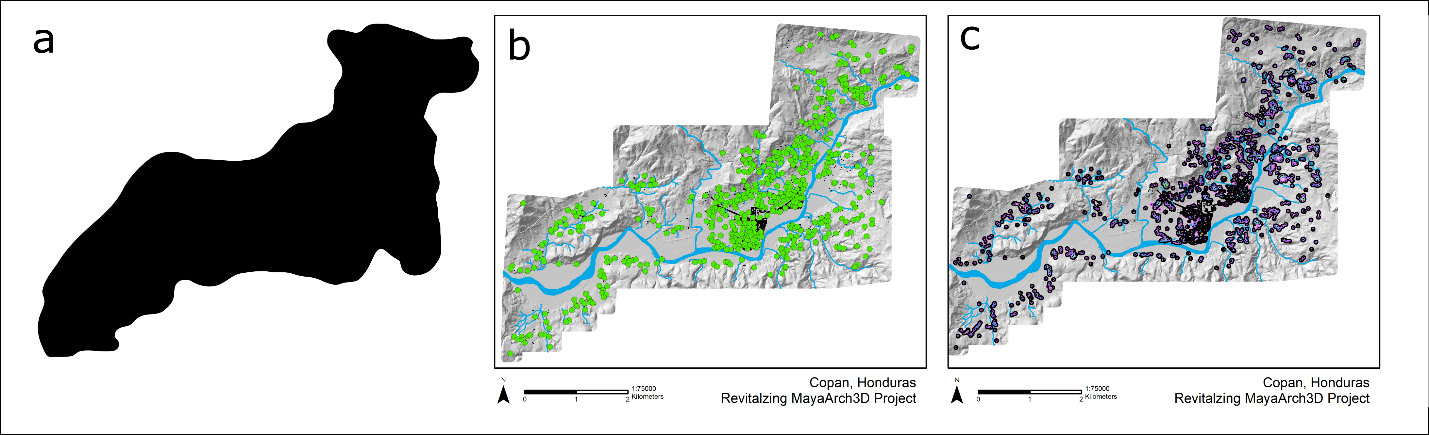
**

**S9 Fig**. **Copán maps**. Scaled map (see Fig 2), settlement map (see S1 Fig), and kernel density map (see S2 Fig) of Copán. Maps by HRR; figure by AET.

**
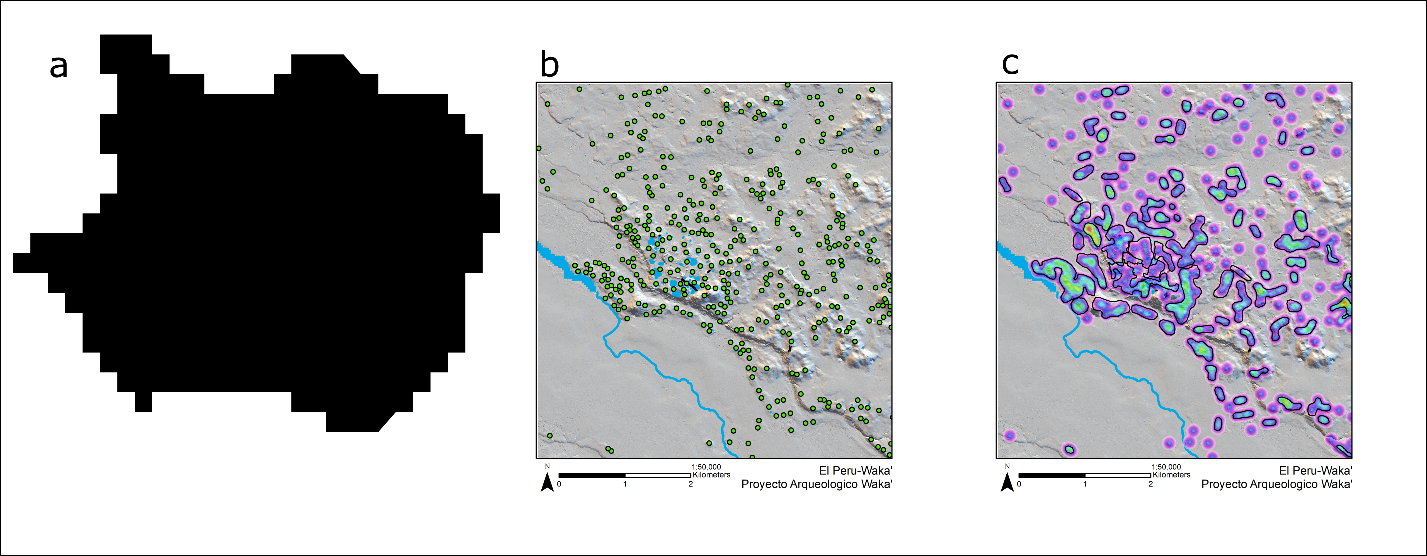
**

**S10 Fig**. **El Perú-Waka' maps**. Scaled map (see Fig 2), settlement map (see S1 Fig), and kernel density map (see S2 Fig) of El Perú-Waka’. Maps by DBM; figure by AET.

**
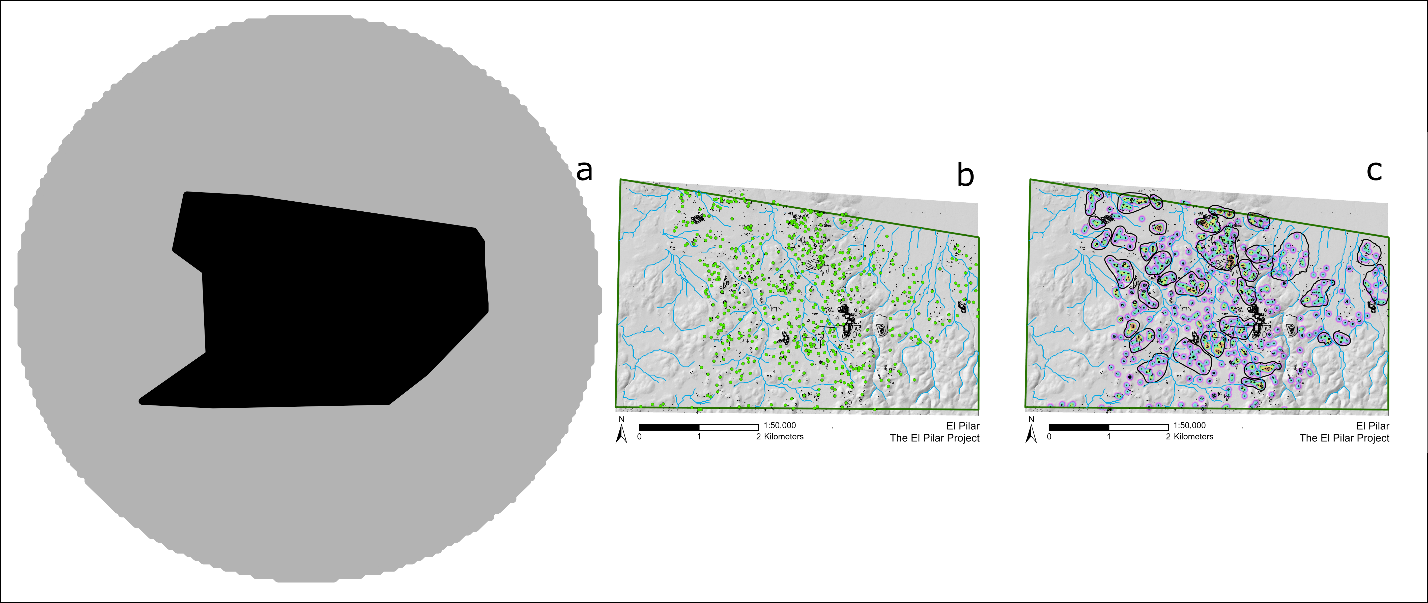
**

**S11 Fig**. **El Pilar maps**. Scaled map (see Fig 2), settlement map (see S1 Fig), and kernel density map (see S2 Fig) of El Pilar. Maps by SWH; figure by AET.

**
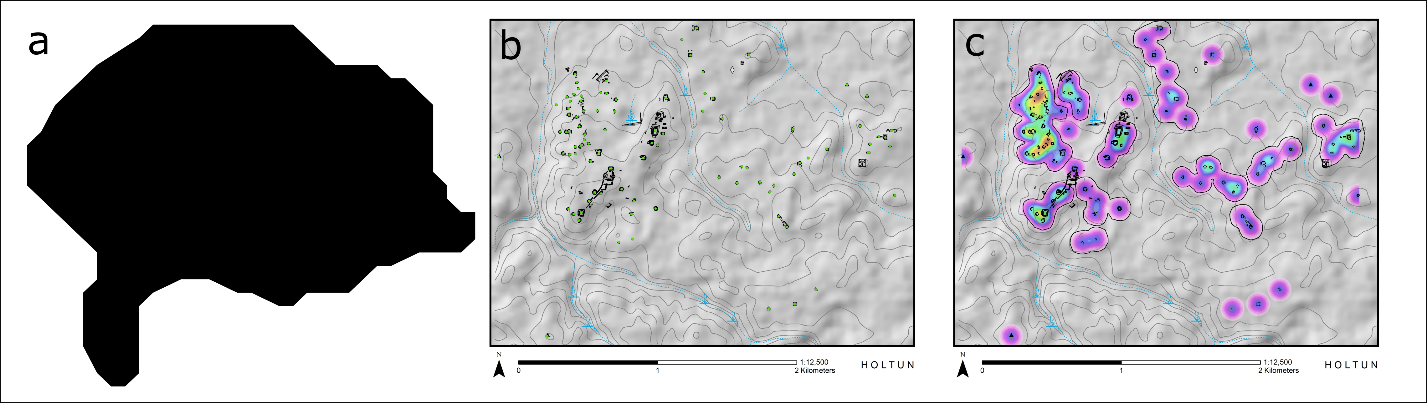
**

**S12 Fig**. **Holtun maps**. Scaled map (see Fig 2), settlement map (see S1 Fig), and kernel density map (see S2 Fig) of Holtun. Basemaps derived from a freely available ASTER GDEM version 2. Maps by MRGP; figure by AET.

**
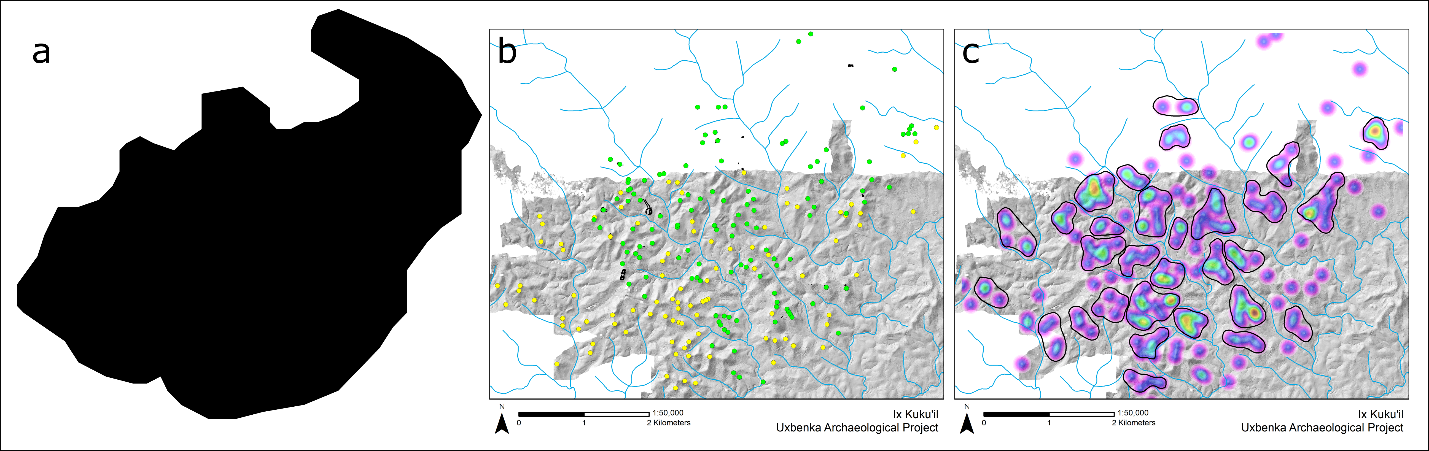
**

**S13 Fig**. **Ix Kuku’il maps**. Scaled map (see Fig 2), settlement map (see S1 Fig), and kernel density map (see S2 Fig) of Ix Kuku’il. Maps and figure by AET.


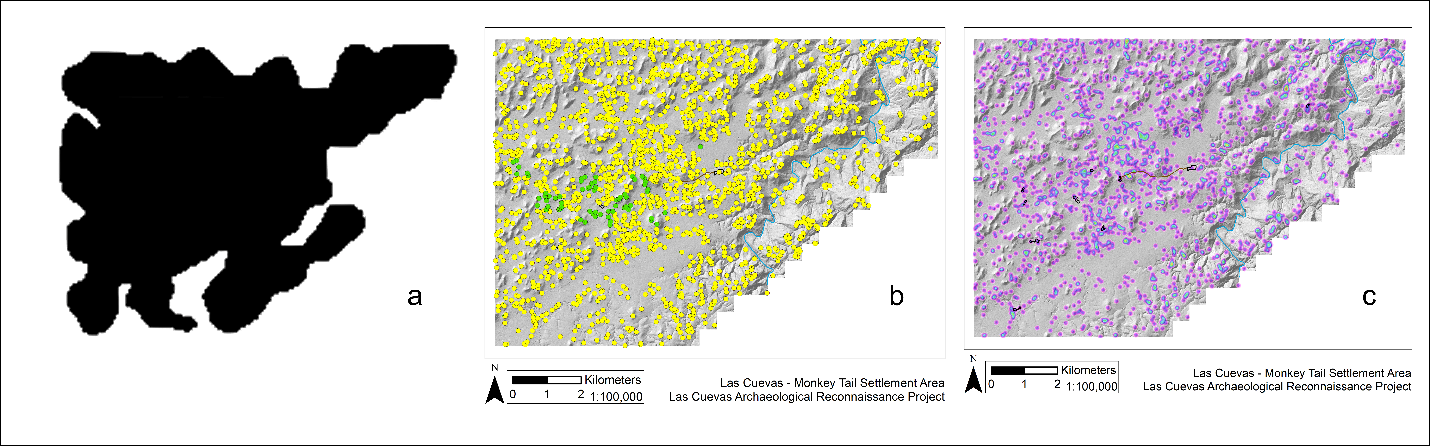


**S14 Fig**. **Las Cuevas-Monkey Tail maps**. Scaled map (see Fig 2), settlement map (see S1 Fig), and kernel density map (see S2 Fig) of Las Cuevas-Monkey Tail. Maps by SMM; figure by AET.

**
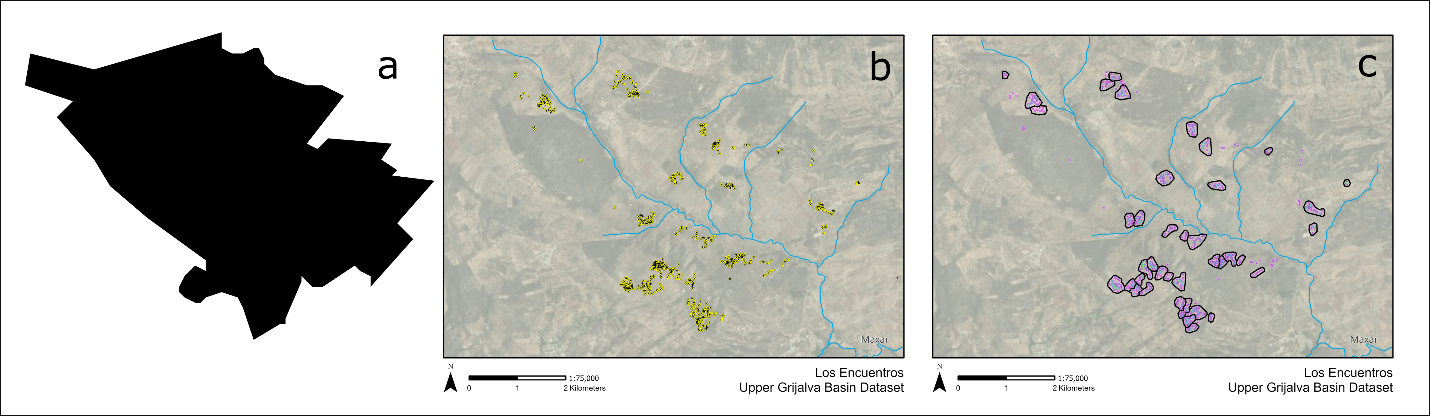
**

**S15 Fig**. **Los Encuentros maps**. Scaled map (see Fig 2), settlement map (see S1 Fig), and kernel density map (see S2 Fig) of Los Encuentros. Basemap consists of 0.6m resolution imagery that is the intellectual property of Esri, Maxar, and Earthstar Geographics and is used herein under license. Copyright 2020 Esri, Maxar, and Earthstar Geographics and their licensors. All rights reserved. Maps by KSM; figure by AET.

**
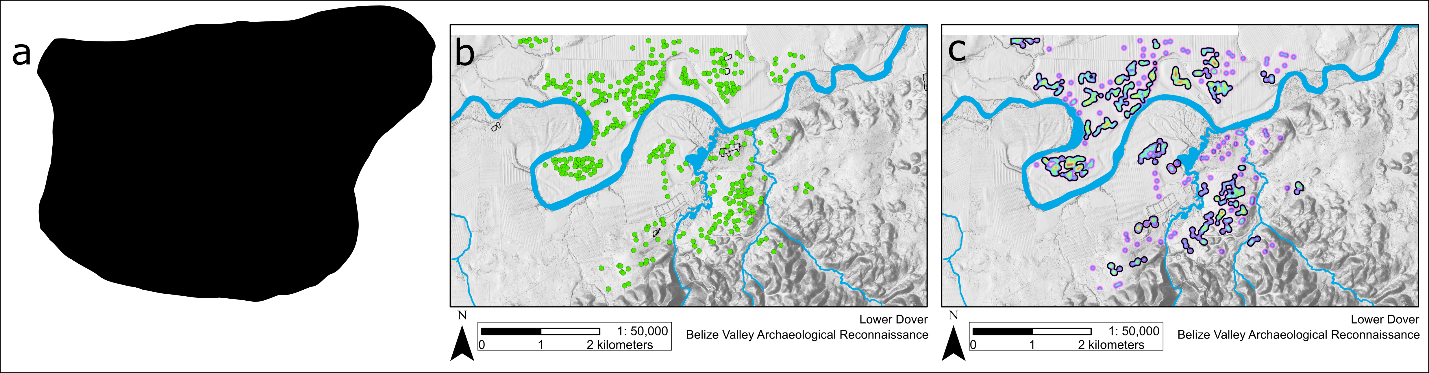
**

**S16 Fig**. **Lower Dover maps**. Scaled map (see Fig 2), settlement map (see S1 Fig), and kernel density map (see S2 Fig) of Lower Dover. Maps by JPW; figure by AET.

**
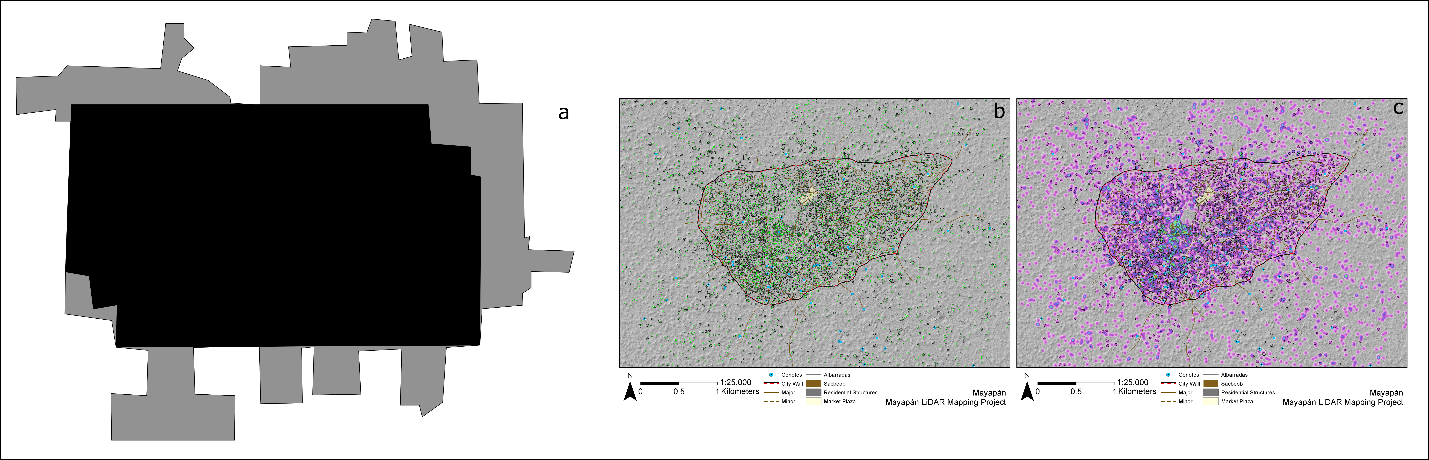
**

**S17 Fig**. **Mayapan maps**. Scaled map (see Fig 2), settlement map (see S1 Fig), and kernel density map (see S2 Fig) of Mayapan. Maps by TSH; figure by AET.

**
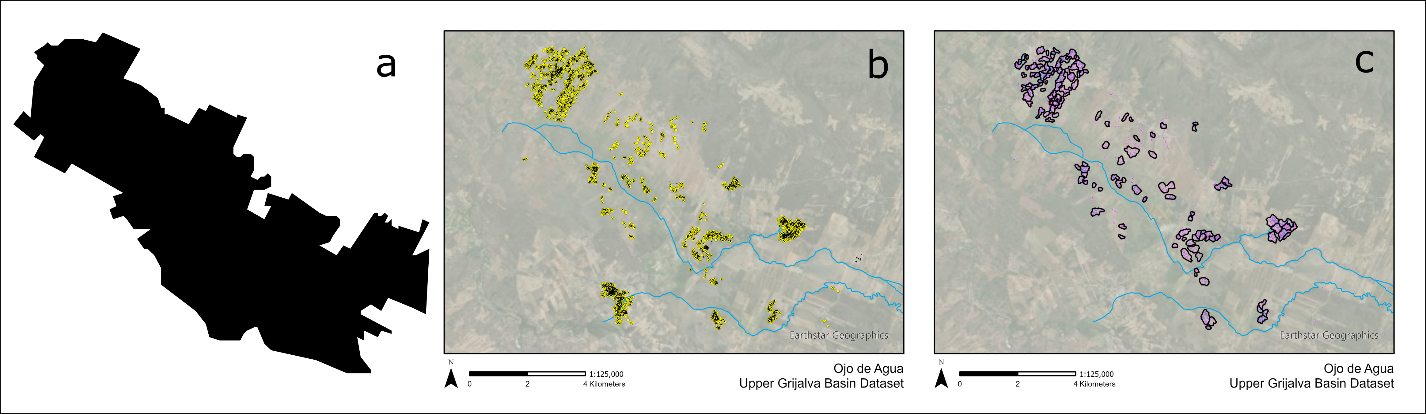
**

**S18 Fig**. **Ojo de Agua maps**. Scaled map (see Fig 2), settlement map (see S1 Fig), and kernel density map (see S2 Fig) of Ojo de Agua. Basemap consists of 0.6m resolution imagery that is the intellectual property of Esri, Maxar, and Earthstar Geographics and is used herein under license. Copyright 2020 Esri, Maxar, and Earthstar Geographics and their licensors. All rights reserved. Maps by KSM; figure by AET.

**
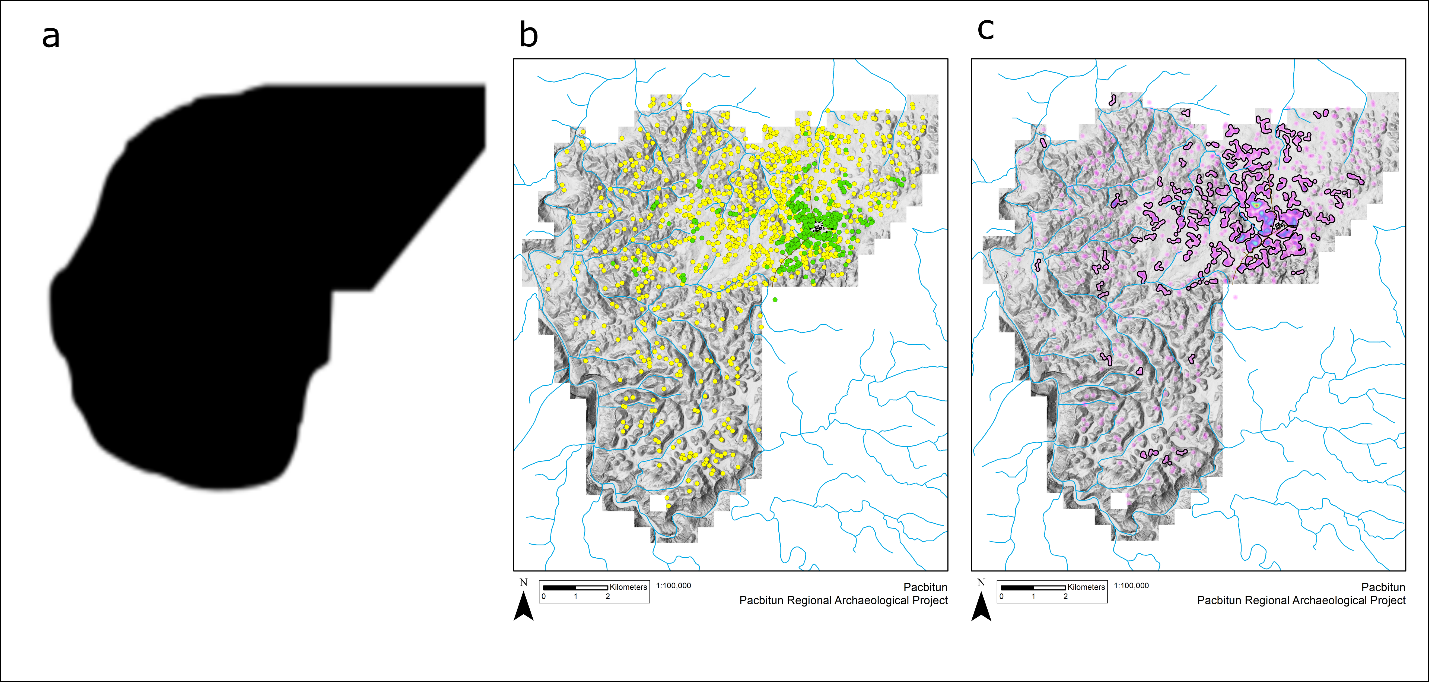
**

**S19 Fig**. **Pacbitun maps**. Scaled map (see Fig 2), settlement map (see S1 Fig), and kernel density map (see S2 Fig) of Pacbitun. Maps by GM; figure by AET.

**
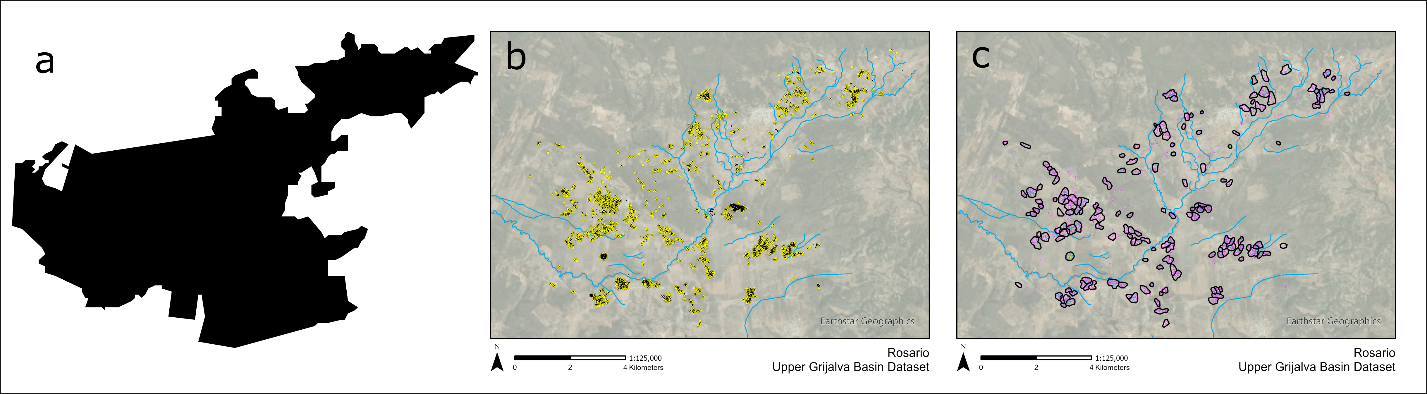
**

**S20 Fig**. **Rosario maps**. Scaled map (see Fig 2), settlement map (see S1 Fig), and kernel density map (see S2 Fig) of Rosario. Basemap consists of 0.6m resolution imagery that is the intellectual property of Esri, Maxar, and Earthstar Geographics and is used herein under license. Copyright 2020 Esri, Maxar, and Earthstar Geographics and their licensors. All rights reserved. Maps by KSM; figure by AET.

**
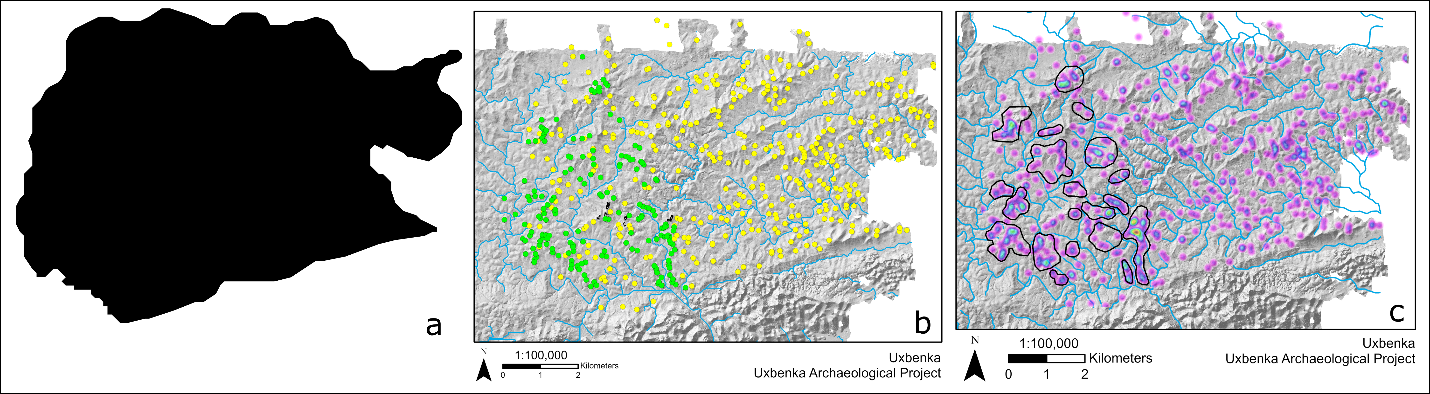
**

**S21 Fig**. **Uxbenká maps**. Scaled map (see Fig 2), settlement map (see S1 Fig), and kernel density map (see S2 Fig) of Uxbenká. Maps and figure by AET.

**Supplemental Tables**

**S1 Table.**

| **Center** | **Boundary Effect Comments** |
| --- | --- |
| Aguacate Lagoon | Modern agricultural practices likely plowed over, destroying households. |
| Almon Plett | Modern agricultural practices likely plowed over, destroying households. |
| Altar de Sacrificios | Sections of settlement have been covered up or wiped away by the changing course of the Rio Chixoy. |
| Baking Pot |  |
| Buenavista del Cayo | Given the size of the civic ceremonial core architecture and settlements visible on lidar data, there are likely additional, non-ground truthed settlements associated with Buenavista del Cayo. |
| *Cadena* | Modern agricultural practices likely plowed over, destroying households. |
| Caracol | Ancient settlement likely extends into modern day Guatemala and are not included in the analysis. |
| Chunchucmil | Additional settlements in the periphery that are visible on lidar were not included. |
| Copán |  |
| El Perú-Waka' | Pedestrian survey captures a large portion of the settlement, but the settlement likely extends an additional 40 km2 beyond the area included in this study |
| El Pilar | pedestrian survey captures much of the settlement but based on lidar, settlement likely extends 2-3 km beyond the area that is included in the study. Future field verification will confirm the presence of archaeological features (Horn and Ford 2019). |
| Holtun | Given the size of civic ceremonial core architecture, Holtun likely has additional settlement but the project does not yet has lidar at the time of writing. |
| Ix Kuku’il | Pedestrian survey supplemented with households identified on lidar data. Additional settlement may extent to the north and west. |
| Las Cuevas-Monkey Tail | Northwesternmost parts of the settlement may be part of greater Caracol. |
| Los Encuentros | Although structures are not as badly damaged by agricultural activities as they would be in more densely populated regions, many structures were clearly destroyed by plowing, resulting in artifact scatters noted in the survey, but not always assigned a house-group. Additionally, due in part to the pedestrian survey, it seems that structures are disproportionately under-recognized in forested areas. |
| Lower Dover |  |
| Mayapan |  |
| Ojo de Agua | Although structures are not as badly damaged by agricultural activities as they would be in more densely populated regions, many structures were clearly destroyed by plowing, resulting in artifact scatters noted in the survey, but not always assigned a house-group. Additionally, due in part to the pedestrian survey, it seems that structures are disproportionately under-recognized in forested areas. |
| Pacbitun |  |
| Pescado Creek | Modern agricultural practices likely plowed over, destroying households |
| Rosario | Although structures are not as badly damaged by agricultural activities as they would be in more densely populated regions, many structures were clearly destroyed by plowing, resulting in artifact scatters noted in the survey, but not always assigned a house-group. Additionally, due in part to the pedestrian survey, it seems that structures are disproportionately under-recognized in forested areas. |
| Uxbenká | Pedestrian survey supplemented with households identified on lidar data. Unclear where the boundary is between Uxbenká and Lubaantun to the NE and some of the housemounds may belong to Lubaantun. However, ANN on a smaller sample has the sample ANN ratio. |
| Zibal and Kichpan Uitz | Modern agricultural practices likely plowed over, destroying households. |

Possible boundary effects at centers included in this study.

**Supplemental Texts**

**S1 Text – Center Summaries**

**Aguacate Regional Archaeology Project (Aguacate Lagood, Almon Plett, Cadena, Pescado Creek, Zibal/Kichpan Witz) Background – Eric C. Fries**

The Aguacate region of western Belize covers five minor Maya centers and their surrounding settlement, arranged along a series of hogback ridges northwest of the modern settlement of Spanish Lookout. Settlement features are found mostly on the upper portions of these ridges, as creeks running along the foot of each ridge create a *bajo* environment downslope. The centers themselves are located on karst outcrops or ridges, and settlement is mostly on low rises, following the pattern predicted for the area by Fedick (1995). Each center consists of a single plaza of roughly similar dimensions (~100 m by 50 m) surrounded by monumental construction, with the exception of Zibal and Kichpan Uitz. Zibal is a monumental plaza group similar to the centers and Kichpan Uitz is a single large pyramidal structure with smaller range structures atop a nearby hill; these two monumental groups are treated as one center in this analysis. Ceramics typologies indicate that all of the centers were occupied from the Early Classic through the Late Classic (250-900 CE), with the exception of Almon Plett, where ceramics indicate an occupation sequence starting in the Late Preclassic and ending before the onset of the Late Classic. Additionally, Aguacate Lagoon has some evidence of Terminal Classic occupation where the upper platforms of monumental structures were repurposed as walled residential areas.

The Aguacate Regional Archaeology Project (ARAP) builds upon the work of the UCLA Maya Archaeology Project (UMAP), which investigated the site of Aguacate Lagoon from 2010 to 2012. Settlement survey around the Aguacate Lagoon monumental cluster located a large number of settlement features in the surrounding landscape, much of which has been cleared for agriculture and cattle pasture. After UMAP concluded work at Aguacate Lagoon, ARAP continued to document ancient Maya settlement in the area through a combination of remote sensing using satellite imagery, and pedestrian survey. Many features in the region are heavily disturbed; some exist only as footprints, visible on satellite imagery as white rectilinear marks in plowed fields and identifiable on the surface through the presence of cut stone blocks and dense artifact scatters. Therefore, survey protocol was developed through an iterative process of identifying potential sites, ground-truthing them, and using the results to guide identification of further potential sites in the next remote sensing cycle. At present, 347 household groups have been identified across a total study area of 100 km^2^, broken down by center as follows: Aguacate Lagoon, 37 groups in 15 km^2^ (33 by pedestrian survey and 4 by remote sensing); Cadena, 16 groups in 11 km^2^ (9 by pedestrian survey and 7 by remote sensing); Almon Plett, 69 groups in 20 km^2^ (46 by pedestrian survey and 23 by remote sensing); Pescado Creek, 82 groups in 20 km^2^ (47 by pedestrian survey and 35 by remote sensing); Zibal and Kichpan Uitz, 143 groups in 30 km^2^ (122 by pedestrian survey and 21 by remote sensing). Many of these groups were initially identified via remote sensing and later ground-truthed during pedestrian survey.

**Belize Valley Archaeological Reconnaissance (Baking Pot and Lower Dover) Background – John P. Walden**

Baking Pot and Lower Dover are two Classic Maya centers situated in the Belize River Valley in modern-day western Belize. Baking Pot which is situated in the western-central Belize River Valley is almost entirely on a fertile alluvial plain, which is agriculturally productive for both modern agriculture and hand cultivation. The soils of the region likely supported cacao in prehistory. Baking Pot is situated close to the Belize River and the hinterland contains several seasonal streams which run from the foothills on the southern flank of the Belize River Valley. These hills form the main source of limestone. The Lower Dover civic-ceremonial center is 7 km east of Baking Pot and is at a point in the valley where the southern foothills extend north to the river. Subsequently, unlike Baking Pot, much of the southern settlement of Lower Dover is on shallow hillside soils, which were still relatively productive under traditional hand cultivation (if not modern agriculture; Awe, Hoggarth, and Helmke 2014; Walden 2021). However, the limestone outcrops in this region provided easy access to construction materials. Upper and Lower Barton Creeks run from the Belize River south into the foothills. Upper Barton Creek was navigable by canoe and connected Lower Dover to the small center of Lower Barton Creek to the south. The northern half of Lower Dover forms the settlement of Barton Ramie which is situated on fertile alluvium (like Baking Pot). Baking Pot was occupied from the Middle Preclassic period (900-300 BC). The civic ceremonial core probably rose at some point in the Late Preclassic (300 BC-AD 300). By the Early Classic period (AD 300-600) Baking Pot was one of the largest political centers in the valley architecturally (~284,000 m^3^; Walden et al. 2019), and demographically (~6000 people; Helmke and Awe 2012; Hoggarth 2012). The Lower Dover civic ceremonial center dates to the Late Classic period (AD 600-900; Guerra and Awe 2017). In contrast, the larger minor centers in the hinterland were settled in the Middle Preclassic and grew substantially in the Late Preclassic. By the Terminal Preclassic/Early Classic transition the center had a population of ~1500 people. This increased to ~2400 people by the Late Classic when the civic ceremonial center was constructed (~148,000 m^3^; Walden 2021). Both centers collapsed in the Terminal Classic when the civic ceremonial cores were abandoned. It increasingly looks like commoner populations abandoned the region around this time, and then reoccupation occurred in the Late Postclassic (Hoggarth et al. 2014).

Settlement survey in the region began with the efforts of Willey and colleagues (1965) at Baking Pot, Barton Ramie, Floral Park, and Spanish Lookout. Subsequently survey at Baking Pot was undertaken by James Conlon (Conlon and Ehret 2001) and Julie Hoggarth (Hoggarth et al. 2010; see also Helmke et al. 2020). The majority of Baking Pot has been subjected to full coverage pedestrian survey. In addition, ~60 mounds have been subjected to excavations (of which 100% showed evidence of Late Classic occupation). The Western Belize LiDAR consortium provided LiDAR data in 2015 (Ebert, Hoggarth, and Awe 2016). While most of Baking Pot was surveyed by this point, the new settlement data confirmed known patterns and was fundamental to survey efforts at Lower Dover. Several small surveys in the vicinity of Lower Dover were undertaken (Driver and Garber 2004; Petrozza 2015), full coverage pedestrian survey of the Lower Dover polity was undertaken in 2016 and a center settlement database was created which included Barton Ramie (Walden 2021). Excavation revealed Late Classic construction at all 96 households (20% of the center) sampled at Lower Dover. The two most important comparative findings from settlement research in the two centers is that Baking Pot was more politically and demographically centralized than Lower Dover. Political centralization is evident by the fact 90% of monumental construction occurred in the Baking Pot core (versus 10% by intermediate elites) whereas at Lower Dover only 70% of monumental construction was in the core (versus 30% by intermediate elites; Walden 2021; Walden et al. 2020). Baking Pot was clearly the focus of the most dramatic settlement aggregation in the area, Lower Dover was demographically disembedded. The vast amount of commoner population resided in several large intermediate elite headed districts.

**Caracol Archaeological Project (Caracol) Background – Adrian S.Z. Chase**

Caracol was one of the largest Classic Period Maya cities and served as the political capital for a regional state encompassing minimally 5,544 square kilometers (AF Chase and Chase 1998a:17) as well as the cities of Tikal and Naranjo during its maximum extent during the early part of the Late Classic Period (AF Chase and Chase 2020a, 2021; DZ Chase and Chase 2003). The city, or center, of Caracol covered about 200 square kilometers in Belize with an additional estimated 40 square kilometers in Guatemala; the center occupied land between the Mopan/Chiquibul and Macal Rivers in the foothills of the Maya mountains on the Vaca Plateau (AF Chase, et al. 2020a:345-347; DZ Chase, et al. 2020c:110-111). The ancient residents of this center completely modified their landscape over about 1500 years of occupation into a fully anthropogenic cityscape (AF Chase and Chase 2016; AF Chase, et al. 2020a). They interconnected nodes of monumental architecture with a dendritic causeway system centered on downtown Caracol (AF Chase and Chase 2001). This system ultimately linked together at least 18 of Caracol’s 25 monumental nodes (ASZ Chase 2021, in press 2022), and these distributed nodes acted as urban districts that administered the city and provided urban services to the city’s residents (ASZ Chase 2016b). Their placement also ensured widespread marketplace access (AF Chase, et al. 2015; DZ Chase and Chase 2014b, 2020b) that encouraged household production of crafts for exchange in the marketplace (AF Chase and Chase 2015). The non-monumental parts of this center consisted of plazuela groups (AF Chase and Chase 2014a), agricultural terraces (ASZ Chase and Weishampel 2016; AF Chase and Chase 1998b; Murtha 2009), and residential reservoirs (ASZ Chase 2016a). During its apogee around 600 CE, Caracol possessed a population of over 100,000 people (AF Chase and Chase 1996:2-3; DZ Chase and Chase 2017:208-209) with initial settlement starting around 600 BCE (AF Chase and Chase 2006:42) and lasting until about 900 CE (AF Chase and Chase 2007; DZ Chase and Chase 2017:216-217).

The Caracol Archaeological Project (CAP) has conducted nearly four decades of research and excavation at Caracol since 1983 after prior research near the center’s urban core in the 1950s by the university of Pennsylvania (AF Chase, et al. 2020b; DZ Chase and Chase 2017:189-198). CAP research initially focused on identifying urban limits through an extensive pedestrian survey effort (AF Chase 1988; AF Chase and Chase 1987); however, after surveying 23 square kilometers between 1986 and 2003, no formal boundary had been identified (DZ Chase and Chase 2017:195-196). This changed with the addition of lidar data from flights in 2009 and 2013 (AF Chase, et al. 2014; AF Chase, et al. 2011) that facilitated the identification of a potential center boundary based on a drop off in intensive terracing on hilltops, hillslopes, and valley bottoms to just valley bottom terracing in tandem with a drop-off in settlement (ASZ Chase 2016b:Figures 1, 4, and 5; ASZ Chase and Weishampel 2016:365). Research with lidar has identified about 200 square kilometers of settlement area in Belize alone with over 7709 residential groups (ASZ Chase 2021; AF Chase, et al. in press 2021), over 177 square kilometers of agricultural terracing (AF Chase and Chase 1994:5; AF Chase, et al. 2014:8685-8686; AF Chase, et al. 2011:397), and over 1590 residential reservoirs (ASZ Chase 2016a). However, these values provide under-estimates that exclude features in Guatemala and those invisible within the lidar dataset (AF Chase, et al. in press 2021).

**El Perú-Waka’ Background – Damien B. Marken**

The ruins of the El Perú-Waka’ urban core sit atop the southwest corner of the Petén Karst Plateau. This core is defined by topography (the plateau escarpment to the south and west) and an enigmatic non-hydrological, “canal” feature on the north (Marken et al. 2019). While center’s overall spatial form varied throughout its occupation (300 BCE-1000 CE; see Eppich, Marken and Meléndez 2022), during much of the Classic, the compact Waka’ urban core (1.38 km^2^) was one of the most heavily occupied places across the Maya lowlands (Marken et al. 2020; see also Canuto et al. 2018; Marken 2015), with a synchronic structure density of 881 strs/km^2^, and an estimated maximum absolute population of 3,307 people/km^2^ during its Late Classic height (Marken and Ricker 2023). Surrounding this core is a multi-functional periurban zone with heavily settled sectors interspersed between sparsely populated, but intensively utilized areas. Beyond this, the city hinterlands extend across an undefined karstic landscape of ridges and hills to the north and east.

The current analysis examines a 29.89 km^2^ lidar DEM clip around the Waka’ urban core (Figure 4, S1 Figure). Within this area 2,827 ground-truthed ancient structures were organized into a variety of settlement group types: single-structure households, multi-structure patios, multi-patio groups and public monumental complexes (Marken et al. 2020). Spatial definition of settlement groups combined topography and spatial proximity (50 m buffer), resulting in 421 settlement groups. ANN (Table 2) and KD analyses (Figure 4; S2 Figure) were conducted on this sample in ArcMap 10.7.2.

**El Pilar Archaeological Project (El Pilar) Background – Sherman W. Horn III**

El Pilar is a large Maya center situated along the eastern edge of the karstic limestone ridge-lands of the greater Petén. More than 150 hectares of monumental architecture, built in a 2-km-swath across what is now eastern Belize and western Guatemala, comprise the El Pilar epicenter, which is surrounded by dense residential settlement, smaller civic architectural groups, and two minor centers (Ford and Horn 2017, 2018a; Horn et al. 2020). El Pilar sits at an ecotone atop a roughly north-south running limestone escarpment, with deeply incised terrain descending eastward to low-lying plains, and ridge-lands transitioning to foothills before reaching the Belize River valley around 10 km to the southeast. The elevated area west of the escarpment edge, containing the majority of mapped ancient residential features, consists of karstic hilltops interspersed among flatlands, with a few seasonal stream courses providing additional relief. Construction activity in the El Pilar core spans nearly 2,000 years in unbroken sequence, beginning in Middle Preclassic times and ending in the Terminal Classic (c. 800 BCE – 1000 CE; Wernecke 2005). Settlement surveys in the upper Belize River area suggest some ancient residences around El Pilar have roots in the Middle Preclassic (Fedick and Ford 1990; Ford and Fedick 1992), although the extent of early settlement is not clear. Populations expanded across the local area in the Late Preclassic (c. 300 BCE – 200 CE), and this filling in of the landscape was paralleled by massive investment in monumental architecture at the city center.

Research at El Pilar focuses on documenting cultural remains and vegetation communities across the 20 km^2^ El Pilar Reserve for Archaeological Flora and Fauna. The acquisition of aerial lidar data in 2012 initiated the current program of full-coverage survey by the El Pilar Project (Ford 2014; Pingel et al. 2015), which has mapped 14 km^2^, or about two-thirds of the reserve, at time of writing. Since 2014, these pedestrian surveys have been instrumental in developing methods to validate lidar image analyses and efficiently map large areas (Ford and Horn 2018b; Horn and Ford 2019), and they have identified 556 primary residential units across the surveyed area. We define primary residential units as groups of two or more associated mounds – or one mound built on a small platform/*plazuela* – based on ethnographic studies of traditional Lowland Maya residences (e.g., Zetina and Faust 2011), which document a minimum of two structures that comprise a family’s primary home: a domicile, and a detached cooking area or kitchen. The remains of households’ primary residences are the fundamental units we use to derive population estimates, investigate land use and environmental interactions, and examine settlement patterning to discover meaningful social groupings, such as neighborhoods.

**Holtun Archaeological Project (Holtun) Background – M. Rodrigo Guzman**

The site of Holtun is an intermediate civic center in the northeast of the department of Peten, Guatemala, or the Central Maya Lowlands. The settlement of Holtun is located on karstic hills in the south of the Yaxha-Sacnab lakes, within the Central Lakes region. The site is adjacent to the modern village of La Maquina, in the municipality of Flores, 12 km southwest of the monumental site of Yaxha (Fialko 2011, Kovacevich et al. 2011: 226). The location of the center is approximately 35 kilometers southeast of Tikal and 12.3 kilometers southwest of Yaxha (Ponciano 1995: 485). Since 2010, Holtun Archaeological Project has conducted investigations in the area with the sponsorship of the University of Central Florida (Callaghan et al. 2017).

The center of Holtun is a modest size settlement, constituted by at least 350 structures organized around patios and plazas (Guzman 2019, 2020). Some of the plaza groups are characterized by the presence of monumental mask facades, or mascarones. One of the plaza groups contains an architectonic compound for astronomical observations and ritual commemorations, or E-Group (Fialko 2011, Kovacevich et al. 2011). The plaza and patio groups are organized in clusters on top of the hills of the tropical karstic landscape. The hills are surrounded by a system of ravines. Although a substantial body of water is not evident in the landscape, the ravines contain at least 4 water springs and several natural pools from moisture filtering through the sediments in the beds of the ravines (Guzman 2017, 2019).

Holtun developed social complexity during the Middle Preclassic period (1000 – 800 BCE). The community of Holtun experienced a cultural fluorescence during the Late Preclassic Period (250 BCE – 250 CE), and then a depopulation or an abandonment in the course of the Early Classic Period (250-500 CE). Later, the settlement experienced a modest repopulation during the Late Classic period (500-800 CE) until a definitive abandonment during the Terminal Classic period (800-900 CE). Social complexity at Holtun was catalyzed by the inclusive and communal rituals in the central plazas and the patterns of production and consumption in the households (Callaghan et al. 2017). The distribution of the settlement in Holtun suggests that the plazas, as the infrastructure that supported the social and ideological power, were placed strategically to demarcate key elements of the landscape such as natural resources and water (Guzman 2020).

**Las Cuevas Archaeological Project (Las Cuevas and Monkey Tail) Background – Shane M. Montgomery and Holley Moyes**

The Las Cuevas study area, situated within the Chiquibul Forest Reserve of western Belize, straddles the southeastern edge of the Vaca Plateau. Low modified hills, constricted valleys, steep-sided ridges, and low-relief, seasonally inundated depressions (pocket *bajos*) are common throughout the landscape. To the east, the Raspaculo and Macal drainages form a rugged divide between the karstic plateau and the metamorphic and granitic materials exposed throughout the Maya Mountains (Bateson 1972). Thin soils characterize much of the plateau, which erode easily to reveal the underlying limestone (Penn et al. 2004). Hillslopes throughout the Las Cuevas area have been stabilized through the expansive construction of masonry terraces, and ditched field systems were recently documented in bottomlands on the northern and southern peripheries of the study area (Montgomery 2019). Two major centers of roughly equal size—Las Cuevas and Monkey Tail—likely serviced much of the population in this portion of the plateau, although eight additional minor centers were present as well. Las Cuevas, occupied for a brief period during the Late and Terminal Classic periods (ca. 700—900 CE), represented a unique example of the direct integration of surface architecture with an expansive cave system, suggesting a dual ritual and civic role. Limited excavation and mapping at Monkey Tail, three kilometers to the east, suggests that the center was comparable in size, function, and importance during these periods (Ramos 2013:114), with a moderate density of residential groups scattered amongst the undulating terrain between the two sites.

The Las Cuevas Archaeological Reconnaissance Project (LCAR) began research in 2011 with the goal of understanding the role of Las Cuevas as a potential pilgrimage center during its apogee in the Late and Terminal Classic periods (Moyes et al. 2012). Investigations focused both on excavations within the ceremonial core and systematic settlement survey and testing within select hinterland areas, mainly to the west of Las Cuevas near the sites of Ta’ Ek’, Cahal Xux, and Ox Tun. Lidar data for LCAR was acquired by the National Center for Airborne Laser Mapping (NCALM) in 2013 through a collaborative effort between multiple archaeological projects established in western Belize (Chase et al. 2014), allowing the project a detailed view of anthropogenic constructions across the landscape. Preliminary settlement research in the Las Cuevas region was initiated by Pedro Carvajal (2016) as part of his master’s research, analyzing 222 km^2^ of lidar-derived data between Ix Uo and the Chalillo Dam to the north. Combined with two seasons of semi-systematic pedestrian survey co-supervised by Dr. Mark Robinson, Carvajal documented 272 major residential groups within the 95 km^2^ territory surrounding Las Cuevas, with a total of 878 structures. Utilizing Local Relief Modeling (LRM) and point cloud visualizations, Moyes and Montgomery (2016; 2019) returned to the aerial lidar to re-examine the data set for more subtle residential features not detected in the Carvajal analysis, including hillslope groups, residences lacking substantial platforms, and households with informal layouts. Further pedestrian survey during the 2017 and 2018 field seasons resulted in the verification of 66 additional residential groups (Montgomery 2018; 2019). The original settlement data resulted in the remote documentation of 1678 residential groups and excluded isolated structures, platforms lacking visible superstructures, and architectural constructions displaying attributes of hilltop shrines. These features were omitted as residential function could not be definitively ascribed without additional excavation; however, the constructions were reintroduced into the residential total (n=1953 residential groups; 16.4% increase in residential groups) in the final analysis as overall settlement distribution did not change dramatically. Overall, the Las Cuevas area averages approximately three structures per group, with an average of 21 groups per km^2^. Late/Terminal Classic settlement within the Las Cuevas area is represented through clustered scatters of residential groups, dissipating to the east beyond Monkey Tail, where terrain becomes unfavorable and identified household groups are reduced by 47 percent per km^2^. Several zones of higher density are detectable within the study area, namely on the southern slopes and low hills between Las Cuevas and Monkey Tail, the gentle slopes northwest of Ox Tun, the restricted valley northwest of Yum Ka’ax, and the depression-filled landscape spreading north from Xul Te’. If such settlement clusters represent neighborhoods, their divisions and boundaries are likely enforced by natural buffers such as karstic ridges, incised drainages, or seasonal inundated lowlands.

**MayaArch3D Project (Copan) Background – Heather Richards-Rissetto**

Copán, the largest Maya center in the southeast periphery of the Maya Lowlands, is located in the Copán Valley of Honduras about 14 kilometers east of Guatemala. The center itself, situated within the Copán Pocket, is approximately 12.5 km long and 6 km wide with altitudes ranging from 600-1400 masl. Scientific evidence indicates that people settled the area circa 1400 BCE (Hall and Viel 2004) with an introduction of Maya elite accoutrements and sociopolitical control staring with the arrival of *Yax Kuk Mo* in 426 CE. Likely, the area was attractive to the Maya due to its high agricultural potential, access to important resources (such as water, construction materials, granite, kaolin, and limestone), and location near major obsidian and jade sources prompted settlement (W. Fash 2001). From 426-ca 820 CE, a lineage of sixteen dynastic rulers reigned over the political center, which at its peak controlled up to 250 km^2^, and the Pocket itself supported up to 25,000 people during the Late Classic (ca. 600-820 CE). Throughout its history, various lines of evidence ranging from ceramics to strontium isotopic analysis indicates a cosmopolitan history (Gerstle 1988, Miller Wolf and Freiwald 2018), likely influencing household and neighborhood organization.

From 2009-2015, the MayaArch3D Project developed a collaborative 3D WebGIS platform for Maya archaeological studies. Two key components were to create geospatial data (vector and raster) for GIS and acquire airborne lidar data for a 25 km^2^ area surrounding the UNESCO World Heritage site of Copan, Honduras. The original GIS data was georeferenced and digitized using analog maps from the Copan Archaeological Project (PAC 1), which surveyed and instrument-mapped 24 km^2^ surrounding Copan’s civic-ceremonial center (Fash and Long 1983; Richards-Rissetto 2010). These pedestrian survey data were integrated with the MayaArch3D airborne lidar data (von Schwerin et al. 2016). Together, these data provide a dataset with 884 “sites” including architectural complexes, isolated mounds, and individual monuments (primarily stelae) based on modifications of the Harvard Typology (Willey et al. 1978), which classifies sites into five types based on mound height, mound complexity, and construction materials. As for neighborhood organization at Copan, archaeologists posit *sian otots*, centered on water sources, as structuring community dynamics within the Pocket (Fash 1983; Davis-Salazar 2003).

**Mopan Valley Archaeology Project (Buenavista del Cayo) Background – Bernadette Cap**

The Classic Maya center of Buenavista del Cayo was built on ancient floodplain terraces above the Mopan River in west-central Belize. Lidar data has revealed relic channels and oxbows indicating the dynamics of the riverine landscape (Friedel 2021; Yaeger et al. 2016). Buenavista del Cayo was built atop the oldest floodplain terrace, which is composed of deep clay-rich soils suitable for grain crops (Cap 2015). The center was established by the Middle Preclassic period, as indicated by a cenote style E-Group (Ball and Taschek 2004; Yaeger et al. 2016). The first major period of growth occurred in the Early Classic, as Buenavista became a regional capital. Sporadic growth throughout the Late Classic was interrupted near the end of the Late Classic period, in the late AD 700s, when major ceremonial architecture was torn down and burned (Cap et al. 2018). Household occupation also sharply declined around this time (Peuramaki-Brown 2012). Despite that, the center was used for ceremonial events and occupied by a lesser noble family in the Terminal Classic period (AD 800s; Cap and James 2016; Helmke et al. 2008). Just across the river, the occupation chronology of the minor center of Callar Creek appears to be tied to competition between Buenavista and its Late Classic rival just upriver, Xunantunich (LeCount and Yaeger 2010). Callar Creek’s first occupation dates to the Middle Preclassic and it emerged as a center during the transition from the Early Classic to the Late Classic and was abandoned at the end of the Terminal Classic (Kurnick 2013, 2016).

Initial investigations at Buenavista by Joseph Ball and Jennifer Taschek in the late 1980s focused on the monumental core and adjacent household groups and on the Guerra settlement group located 1 km to the south. Yaeger initiated investigations at the site core and adjacent settlement areas in 2007 as part of the Mopan Valley Archaeological Project (MVAP). Callar Creek, a nested community or district of Buenavista, was first mapped by the Xunantunich Settlement Survey (XSS) directed by Wendy Ashmore. It lay at the north end of a settlement transect that extended north from Xunantunich (Leventhal et al. 2010). The project undertook testing in the site center (Ehret 1995), which was followed up by extensive excavations by Sarah Kurnick (2013) as part of MVAP.

In 2013, MVAP comprised part of the Western Belize Lidar Consortium and acquired Lidar data covering a 125 km^2^ zone of the Mopan Valley and adjacent areas (Chase et al. 2014). Pedestrian surveys have focused on settlement around the Buenavista and Callar Creek civic ceremonial core and took place over several seasons (Ashmore 1994; Deluca and Yaeger 2019; Peuramaki-Brown 2012). In total, 2.15 km^2^ have undergone pedestrian survey during which a total of 131 house groups were mapped. An additional 161 possible house groups were identified through examination of Lidar-derived visualizations (e.g., hillshade, slope, open-positive, open-negative, low relief models, etc.). For the purposes of this analysis, we defined house groups as isolated mounds less than 2 m in height and formal or informally arranged mound groups with two to five mounds, all less than 2 m in height. The area presented in this study has an average density of 27 house groups per km^2^, which aligns with Canuto et al.’s (2018) expectation of a rural settlement zone. An important note of caution about this average density statistic: it significantly under-represents the ancient settlement density, as testing the fidelity of the Lidar data in this mosaic landscape, Cap et al. (2018) found that more than half of expected mound groups were not visible, especially those informally arranged and less than 2 m in height. If we were to double the settlement density to 54 groups house groups per km^2^ to account for that, our area would fall near Canuto et al.’s (2018) threshold for peri-urban settlement, 60 groups house groups per km^2^.

**Pacbitun Regional Archaeological Project (Pacbitun) Background – George J. Micheletti**

Pacbitun is a medium-sized center tied to the Belize River Valley, located on the northern foothills of the Maya Mountains in the Cayo District of west-central Belize. The center is set at the cusp of a tropical/subtropical broadleaf moist forest biome and a premontane pine forest biome (Hartshorn et al. 1984). The tropical forest terrain surrounding the center and running north is composed of low rolling hills characterized by crystalline limestone bedrock and deep calcareous soils settled in low-lying areas (Hartshorn et al. 1984; Healy 2020). Fertile soils combined with plentiful annual rainfall of the Belize Valley, averaging around 1500-2000 mm, made this a strategic locale for subsistence farming (Hartshorn et al. 1984; Healy 2020). Additionally, reliable freshwater sources in the form of numerous springs, streams, and creeks in the immediate vicinity of the site are thought to have been one of the driving forces for selecting this settlement locale (Healy 2020). Although the poor, sandy soils of the Mountain Pine Ridge to the south were not suitable for agriculture, its diverse ecology offered an array of floral, faunal, and geological resources for exploitation (Healy 2020; Healy and Emery 2014; Tibbits 2020). Despite the Middle Preclassic period (ca. 900 BC) foundation of the epicenter or civic ceremonial core (Powis 2020), settlement did not begin to expand into the periphery until the Late Preclassic period (300-100 BC) (Healy et al. 2007). Population steadily increased through the Early Classic period (AD 300-550), rose sharply in the Late Classic period (AD 550-800), and surged during the Terminal Classic period (AD 800-900) (Healy et al. 2007). However, by AD 900 Pacbitun was mostly abandoned with some occupation continuing into the Early Postclassic period (AD 900-1250) (Helmke and Ting 2020).

To gain a better understanding of Pacbitun’s settlement demographic, distribution/density, and mound/group configuration, initial settlement research by Paul Healy and the Trent University-Pacbitun Archaeological Project in the 1980s conducted a full coverage survey of Pacbitun’s “core” zone (1 km²) and laid out four intercardinal transects extending 1 km from this zone out into the “periphery” zone resulting in the identification of 225 groups composed of 330 mounds (171 isolated mounds, 159 clustered mounds composing 54 groups) (Healy et al. 2007). Of these, 74 structures were randomly selected for excavation to recover chronological and functional information (Campbell-Trithart 1990; Richie 1990; Sunahara 1995). After the establishment of the Pacbitun Regional Archaeological Project (PRAP) in the late 2000s, approximately 50 new groups were identified during cave, causeway, and ground-stone production research (Spenard 2014; Weber 2011; Skaggs et al. 2020). The acquisition of lidar data of the 104 km² PRAP permit area in 2015, courtesy of the 2013 lidar survey of west-central Belize (Chase et al. 2014), guided periphery investigations of middle and lower-level settlement to document an additional 93 groups through unsystematic pedestrian survey (Micheletti and Powis 2020.). Various methods of lidar visualizations allowed for the identification of 953 potential groups spread across the 104 km² permit area. Thus, a total of 1,321 groups were used for this analysis of Pacbitun’s settlement (368 verified through pedestrian survey, 953 identified through remote sensing).

**Pakbeh Regional Economy Program (Chunchucmil) Background – Scott R. Hutson**

Located in the northwest corner of the Yucatan peninsula, Mexico, Chunchucmil was a large, very densely populated Maya center that reached its peak population at the end of Early Classic period. Covering scarcely more than 15 km^2^, the city housed between 31,000 and 47,000 people at its peak (Hutson 2017). The center occupied a semiarid karst plain elevated approximately 3 m above sea level. The region receives between 700 and 1000 mm of rain annually, making it one of the driest parts of the Maya world. The parent limestone is highly porous, such that rain quickly seeps to the water table, which is located approximately 2 meters below the ground surface. Thus, there are no rivers, streams, or lakes but water is easily accessible in small sinks and excavated wells. The landscape is largely flat, with small hills that do not exceed 1m. Soils are generally thin (20 cm deep) and poor, and about a third of the land surface consists of exposed bedrock. The major soil types consist of dark reddish silty clays (Paleustaffs and Paleustolls, locally known as *kancab*; “red earth”) and darker, deeper soils (locally known as *boxluum*; “black soil”), formed over the last thousand years, with less clay and higher amounts of organic carbon and carbonate (Beach 1998).

Bruce Dahlin initiated what came to be known as the Pakbeh Regional Economy Program and was joined at different times by co-directors Anthony Andrews, Traci Ardren, and Scott Hutson (Dahlin et al. 1998; Dahlin and Ardren 2002; Hutson 2017). Mapping of Chunchucmil began in 1996 and ended in 2006. For the central 9.3 km^2^ of the settlement map, mappers took advantage of pre-existing grids of 20 m by 20 m blocks, a relic of early 20^th^ century *henequen* cultivation, as well as new grids produced with a theodolite. The grids were georeferenced by high-precision GPS and the features within them were mapped largely by pacing. Five fishbone transects extending beyond the central map were surveyed with tape, compass, and GPS. The central 9.3 km^2^ contains 1410 settlement groups, each consisting, on average, of 3.5 houses. Most of these can be considered residential *plazuela* groups. Data from the transects suggest that about 1000 groups of similar size pertain to Chunchucmil but lie beyond the boundaries of our map. The nearest neighbor analysis shows that these groups are relatively evenly dispersed, as opposed to clustered. Thus, kernel density is not particularly helpful in discerning neighborhoods at Chunchucmil. Nevertheless, a system of built walkways broadly organized in a hub and spoke pattern permit the suggestion of 14 neighborhoods of various sizes (Hutson 2016).

**Proyecto Arqueológico Altar de Sacrificios (Altar de Sacrificios) Background – Jessica Munson**

The Upper Usumacinta Confluence Zone (UUCZ) encompasses the region where the Pasíon and Salinas rivers join to form the Río Usumacinta along the modern-day border of Guatemala and Mexico. Aliphat (1994) describes lower stretches of the Usumacinta as a bedrock river with a stable, fault-controlled course; however, the confluence zone represents a much more dynamic fluvial environment. The landscape is characterized by a low-lying plain of rich alluvial soils and meandering channels with slightly elevated areas surrounding seasonally flooded lagoons, oxbow lakes, and numerous remnants of abandoned meander loops, which attracted ancient Maya people to settle along these waterways. Many important pre-Columbian centers were established along rivers because they offered concentrated resources, rich soils, and access to transportation corridors for both early agriculturalists and more mobile forager populations across the Americas (Heckenberger, Petersen, and Neves 1999; Hudson 2004; Rosenswig et al. 2014). For the Maya region, the UUCZ connects multiple major watersheds, and with its rich alluvial deposits and abundant aquatic resources, it should not be surprising that this was an important zone of past human activity. Altar de Sacrificios is the largest recorded center in the UUCZ with its civic ceremonial core strategically situated on high ground along the southern bank of the Pasíon approximately 2 km from the current confluence. Previous investigations by Harvard University focused on an intensive excavation program in the site core along with ceramic analysis from limited house mound excavations to establish one of the earliest continuous ceramic chronologies in the Maya lowlands (Adams 1971; Smith 1972). Recent investigations in the greater Pasíon region indicate semi-mobile groups likely co-existed with more sedentary ones for several centuries after the introduction of ceramics around 1000 BCE at nearby Ceibal (Inomata et al. 2015). At Altar de Sacrificios, populations were probably small and dispersed throughout the Middle Preclassic period with low earthen constructions dotting the landscape. Monumental architecture and ritual activities in Group B coincided with population expansion during the Late Preclassic, but it is unclear if these were sponsored by elites or whether these were more communal ceremonies. By the end of the fifth century CE, however, several carved monuments were dedicated in Group B that included figural representations and the earliest inscription of Altar’s original place name (Houston 1986). Group B continued to be the core of ceremonial activity throughout the Early Classic period until construction and ritual activities shifted to Group A at the beginning of the 7^th^ century CE. Populations continued to grow until the mid-8^th^ century CE when warfare and political fragmentation swept across the greater Petexbatun-Pasion region (Houston 1993; Inomata 2003; 1995). Occupation persisted at Altar de Sacrificios into the Terminal Classic, but the civic ceremonial core was largely abandoned by the end of the 10^th^ century CE.

Initiated in 2016, the Proyecto Arqueológico Altar de Sacrificios (PAALS) has focused on human-environment interactions in the UUCZ to understand the history of occupation and landscape change through extensive settlement survey, household excavations, and geoarchaeological research (Paiz Aragón and Munson 2017; Munson, Mejía Ramón, and Aragón 2019; Munson and Paiz Aragón 2020). PAALS has conducted a set of unmanned aerial system (UAS) Structure-from-Motion (SfM) surveys covering a total of 36 km^2^ to date. From these images we have generated high-resolution topographic models (15 cm/pixel) of the landscape to aid in the identification of cultural features (e.g., mounds, depressions, canals, terraces, etc.) and documentation of river channel migration. A total of 373 mounds have currently been identified in the surveyed area. These are typically low earthen platforms that would have provided a level surface for the construction of perishable structures. Depending on their size, most mounds could have accommodated 2-4 structures and can be considered a household unit. For the purpose of this analysis, individual mounds were classified into “house mound groups” based on mound size (height and basal area) and their proximity to other smaller mounds and depression features. A total of 212 house mound groups were included in the current study. Real-time processing has enabled field verification of 147 (69%) house mound groups. The remaining 65 house mound groups have been identified solely through remote sensing.

**Proyecto Económico de Mayapán Project (Mayapan) Background – Timothy S. Hare**

The Postclassic Maya center of Mayapán is located 40 km southeast of the modern city of Mérida on a flat karstic plain lying just above sea level near a “ring of cenotes” around the rim of the Chicxulub crater (Hare et al. 2014a; Peraza Lope and Masson 2014). The location was probably influenced by the high density of water bearing cenotes in the area. Soils are thin, rarely measuring more than 40 cm in depth. Limestone bedrock is just as common as soil when the surface is cleared of the low forest canopy and scrub vegetation. The level landscape is pockmarked by numerous scattered low hills that are typically only a few meters tall. Structures are commonly located on the tops and edges of the small hills. Occupied hills are frequently modified by leveling or extension. Soils develop and accumulate in the swales between the hills and constitute the main areas of documented agricultural production. Structures are also located on the swales. The focal point of the region is Postclassic Mayapán (A.D. 1150-1450), a walled political center housing over 15,000 people in a 4.1 km^2^ walled zone that functioned as the capital of a confederacy of Northern Lowland centers (Peraza Lope and Masson 2014). Prior to Mayapán's founding, the region was occupied by dispersed populations (Russell 2008; Masson and Peraza Lope 2014a, 2014b, 2014c). For instance, Tichac, a minor center, located less than a kilometer north of Mayapán, was a focal point for houselots distributed loosely across the landscape in all directions during the Late and Terminal Classic. Late/Terminal Classic rural settlement extends through the zone that later became the walled city. Sherds from earlier periods are present across the Postclassic city, but all prior architecture was razed by later construction (Peraza et al. 2006; Peraza and Masson 2014c). Minor centers, surrounded by sprawling rural settlement, were distributed across the surrounding region of at least 40 km^2^ during the Postclassic.

Settlement mapping of Mayapán began in 1948 with a team from the Carnegie Institution (Pollock et al. 1962; Smith 1971; Weeks 2009). This project mapped 4,100 structures within the 4.2 km^2^ walled portion of the site (A.L. Smith 1962). Walls defining boundary walls within the center were mapped for only a small portion of the walled center (Bullard 1952, 1953, 1954; A.L. Smith 1962:208-209). Carlos Peraza’s INAH-Mayapán Project excavated and restored buildings of the city’s primary monumental plazas (e.g., Peraza et al. 1999, 2001; 2002). Brown (1999) followed up with mapping and a series of excavations in the center's residential areas and identified potential wards or neighborhood groups of walled houselots. The Proyecto Económico de Mayapán Project (PEMY) accomplished intensive mapping, surface survey, surface collection, test-pitting program, or horizontal excavations in 36 sample areas inside and outside the city wall from 2002-2009 (Masson et al. 2008). Results of the work have examined the spatial patterning of features such as lanes, plazas, administrative halls and residences. PEMY activities also included Russell’s (2008) survey outside of the city wall. He mapped all features in eight 250 X 1000 m transects radiating out from the wall doubling estimates of residential portion of the site. The 2013 Mayapán lidar Mapping Project documented a 40 km^2^ area of settlement and environmental features around Mayapán using aircraft-borne lidar. Features identified on lidar-based maps were ground-checked and surface collected to assess their function and chronology. 16.08 km^2^ were “lightly” checked (36.38% of lidar area) and 38 locations were intensively checked and mapped. Surface collections were conducted to assign chronology and function to checked locations. We also conducted test excavations at a sample of intensively mapped locations to provide a further basis for estimating the time periods of occupations. Artifact analysis focused on chronological markers and evidence of function and production activities. Further ground checking of the lidar survey area continued in 2015 along with excavations of eight house groups outside the city wall. There are currently 4297 verified house groups mapped within the ground checked area of 20.6 km^2^. Of these, 1246 are clearly defined by mapped property walls, average 1147.2 m^2^ in area, and average 2.1 structures per group. The ground-checked portion does not encompass the entire area of settlement directly linked to Mayapán, but analysis of non-ground-checked features indicates the sample constitutes the majority. The focus of the settlement, urban Mayapán had an average density of approximately 33 people per hectare (pph) but reached 126 pph in parts of the downtown zone (Masson et al. 2014:266). The center wall’s circumference is 9.1 km is the primary impediment to movement in the region, but allowed travel through twelve formal gates. The regional tradition of houselot boundary walls and lanes partitions the cityscape and settlement near the city wall into residential, open, and public spaces. Areas of contiguous residences extended beyond the wall to approximately half a kilometer in all directions. Ten settlement centers, marked by temples, shrines, and often, cenotes, dot the periphery within one to three kilometers of the city wall. Low-density residential settlement sprawls from the city wall to these localities in the outskirts of greater Mayapán. Compared to the urban zone, Postclassic Period residences outside of the center wall were are dispersed (Hare et al. 2014b; Russell 2008).

**Upper Grijalva Basin Dataset (Ojo de Agua, Rosario, and Los Encuentros) Background – Kyle Shaw-Müller**

Ojo de Agua, Rosario, and Los Encuentros were Classic Maya centers located in the Greater Rosario Valley, in what is today southeast Chiapas, Mexico. This valley is the highest and northernmost of the Grijalva River’s Upper Tributaries, which cuts through a 35 km-wide downfold between the Cuchumatán Mountains and Comitán Plateau (de Montmollin 1995:22–23). The Greater Rosario Valley has two upper branches in the northeast and southeast and is constricted by low foothills in its mid-valley, beyond which it descends to the lower valley in the northwest. The wide valley floor is watered by 1000 mm of rain annually and three principal water courses that lead into the Grijalva River, each fed largely by karstic springs: the Santa Inés (a.k.a. El Sabinal) and Ojo de Agua rivers, which flow southwestward into extensive swamps and the Ontelá River, and a separate set of tributaries in the southeast branch that flow south into the San Gregorio River (de Montmollin 1995: 23-27). Before the eastern edges of the Rosario Valley began to be settled by Mayas in the Terminal Formative (300-50 BCE), the valley was inhabited exclusively by the Mizoques (De Montmollin 1995; Lowe 1983). Maya settlement expanded westward through the Proto-Classic, followed by a hiatus during the Early-Middle Classic, and finally the maximum population extent was reached in the Late-Terminal Classic (650-1000 CE) before demographic collapse occurred between 900 and 1000 CE (de Montmollin 1995:3-4).

The Upper Grijalva Basin Dataset (de Montmollin 2018) was compiled from decades of reconnaissance, surveys, and excavations in the region. Most data were collected by teams from the Instituto Nacional de Antropología e Historia and the New World Archaeological Foundation (Blake et al. 2016; Bryant 1981; Lee 1984; Lowe and Mason 1965; Rivero T. 1990), especially during salvage operations which included the Angostura Project (Con 1981; Gussinyer 1973; Martínez and Navarrete 1978). In 1983, 1988, and 1990, de Montmollin conducted systematic surveys of the Greater Rosario Valley, combining these data with earlier findings for comprehensive analyses of the valley (de Montmollin 1989, 1995). He used wide block, full coverage, pedestrian survey to collect these data (de Montmollin 1995:253). Compiled data consist largely of plan-maps for all recorded structures and points for amorphous mounds and scatters. Although de Montmollin took written note of house group quantities for each “site” (i.e., house-cluster), these groups were neither marked on maps nor denoted in spreadsheets. As a result, points for each house group were placed according to structure location and other contextual data. The largest center and accompanying study area (70.11 km^2^) was Rosario. Its Late Classic apical center was Tenam Rosario, and 2276 house groups were identified within the study area. The Los Encuentros center’s study area, to the south, was much smaller (24.7 km^2^) and included 561 residential groups. El Zapote was the main civic-ceremonial center of Los Encuentros and, like Tenam Rosario, was likely built in the Late Classic Period. The Ojo de Agua center was located at a lower elevation in a relatively flatter, wider portion of the valley floor to the West. Its study area was 57.21 km^2^ and contained 2004 house groups. The capital was Ojo de Agua, which was likely occupied since the Formative period (de Montmollin 1995:107), and was located on the study area’s eastern edge.

**Uxbenká Archaeological Project (Uxbenká and Ix Kuku’il) Background – Amy E. Thompson**

Uxbenká and Ix Kuku’il are Classic Maya centers located in the southern foothills of the Maya Mountains in modern-day southern Belize. The foothills, or Toledo Uplands, are characterized by interbedded sandstone and limestone with soils that undergo rapid pedogensis, making them favorable for agriculture (King et al. 1986; Wright et al. 1959). Regionally, perennial streams drain from the northwest to southeast, cumulating from higher elevation areas in the Maya Mountains and fed with freshwater springs throughout the foothills. The foothills are characterized by discrete hilltops and elongated ridges which were preferred locations for the Classic Maya constructions (Dunham 1990; Kalosky and Prufer 2012; Prufer et al. 2015). The earliest founded center in the region, Uxbenká, had its humble beginning centuries later than many other Lowland Maya centers, with the first permanent architecture dating to around 100 CE (Prufer et al. 2011). Populations expanded and monumental architecture was constructed by 200 CE (Prufer et al. 2017). Settlements dispersed across the nearby hilltops, and by 400 CE, kin groups fissioned off forming a new political center, Ix Kuku’il, approximately 7 km from stela plaza to stelae plaza (Thompson and Prufer 2016, 2021). Populations at both Uxbenká and Ix Kuku’il continued to grow until 800-900 CE, when political disintegration swept across the region (Ebert et al. 2014). Ephemeral populations persisted well into the Early Postclassic (Thompson and Prufer 2021) and beyond (Prufer and Kennett 2020).

Beginning in 2005, the Uxbenká Archaeological Project (UAP) focused on human-environment dynamics to understand the development and decline of Uxbenká through extensive settlement survey and excavations in the civic ceremonial core and hinterland households alike (Prufer 2005; Prufer et al. 2011, 2017). The UAP acquired 135 km^2^ of lidar data in 2011, which provided detailed coverage of the landscape resulting in more effective and efficient survey of Classic Maya households (Prufer et al. 2015; Prufer and Thompson 2014, 2016; Thompson and Prufer 2015). Over the course of 13 years, the UAP identified more than 302 household plazuelas through pedestrian surveys of nearly 35 km^2^ (Thompson 2019; Thompson and Prufer 2019). Drawing on previously published literature of regional settlement surveys (Dunham 1990; Hammond 1975; Jamison 1993), systematic pedestrian survey focused on hilltops. In addition to pedestrian survey, lidar relief visualizations were used to identify more than 550 new plazuelas across the landscape (Thompson 2020) that were used in these analyses. In total, Uxbenká contains 568 household plazuelas (180 verified through pedestrian survey, 388 identified solely through remote sensing) across 75 km^2^. At Ix Kuku’il, 122 household plazuelas have been verified through pedestrian survey and an additional 93 households identified through lidar, for a total of 215 plazuelas across 23 km^2^ (Thompson 2020; Thompson et al. 2021; Thompson and Prufer 2021). Uxbenká’s and Ix Kuku’il’s settlement systems exhibit low-density urbanism (sensu Fletcher 2009) with an average of 3.4 building platforms per plazuela group (Thompson 2019: Table 1.4).

**Works Cited for Supplemental Text S1**

Adams, R. E. W.

1971 *Ceramics of Altar de Sacrificios, Guatemala*. Vol. 63, No. 1. Papers of the Peabody

Museum of Archaeology and Ethnology. Cambridge: Harvard University.

Aliphat, Mario M.

1994 *Classic Maya Landscape in the Upper Usumacinta River Valley*. Ph.D. Dissertation in

Archaeology, Calgary: University of Calgary.

Awe, Jaime J., Julie A. Hoggarth, and Christophe Helmke

2014 Prehistoric Settlement Patterns in the Upper Belize River Valley and their Implications for Models of Low-Density Urbanism. In *Special Edition of Acta Mesoamericana in Honour of Pierre R. Colas* Vol 27, edited by Christophe G.B. Helmke, and Frauke Sachse, pp. 263-285. Verlag Phillipp von Zabern Mainz, Germany.

Ball, Joseph W., and Jennifer T. Taschek

2004 Buenavista del Cayo: A Short Outline of Occupational and Cultural History at an Upper

Belize Valley Regal-Ritual Center*.* In *The Ancient Maya of the Belize Valley: Half a*

*Century of Archaeological Research*, edited by James F. Garber, pp. 149-167. University

Press of Florida, Gainesville.

Bateson, J.H.

1972 New Interpretation of Geology of Maya Mountains, British Honduras. *AAPG Bulletin*,

*56*(5):956-963.

Beach, Tim

1998 Soil Constraints on Northwest Yucatan, Mexico: Pedoarchaeology and Maya Subsistence at Chunchucmil. *Geoarchaeology* 13(8):759-791.

Blake, Michael, Thomas A. Lee, Mary E. Pye, and John Clark
2016 *Upper Grijalva River Basin Survey*. Papers of the New World Archaeological Foundation

79. The University of Utah Press, Salt Lake City, UT.

Brown, Clifford T.

1999 *Mayapan Society and Ancient Maya Social Organization*. Ph.D. dissertation, Department

of Anthropology, Tulane University. University Microfilms, Ann Arbor.

Bryant, Douglas D.
1981 *An Introduction and Preliminary Culture Historical Synthesis of Ojo de Agua, Chiapas,*

*Mexico*. Unpublished manuscript in New World Archaeological Foundation files.

Bullard, William R.

1952 Residential Property Walls at Mayapan. *Current Reports* 3:25-29. Carnegie Institution of Washington, Department of Archaeology. Washington, DC.

1953 Property Walls at Mayapan. *Year Book* 52:258-264. Carnegie Institution of Washington. Washington, DC.

1954 Boundary Walls and Houselots at Mayapan. *Current Reports* 13:143-155. Carnegie Institution of Washington, Department ofArchaeology. Washington, DC.

Callaghan, Michael G., Brigitte Kovaceivch, Karla Cardona, Melvin Rodrigo Guzman Piedrasanta, and Dawn Crawford.

2017 La Comunidad Preclásuca en Holtun, Guatemala: Vista del Grupo F. In *XXX Simposio de Investigaciones Arqueológicas en Guatemala*, Tomo 1, 2016 edited by B. Arroyo, L. Méndez Salinas, and G. Aju Alvarez. Pp. 93-106. Museo Nacional de Araueología y Etnología, Guatemala.

Campbell-Trithart, Melissa J.

1990 *Ancient Maya Settlement at Pacbitun, Belize*, Unpublished Master's thesis, Department of

Anthropology, Trent University, Peterborough, Ontario.

Canuto, Marcello A., Francisco Estrada-Belli, Thomas Garrison, Stephen D. Houston, Mary Jane Acuña, Milan Kováč, Damien Marken, Philippe Nondédéo, Luke Auld-Thomas, Cyril Castanet, David Chatelain, Carlos R. Chiriboga, Tomáš Drápela, Tibor Lieskovský, Alexandre Tokovinine, Antolín Velasquez, Juan C. Fernández Díaz, and Ramesh Shresta

2018 Airborne laser scanning of northern Guatemala: A Reckoning with Ancient Lowland Maya Complexity. *Science* 361: eaau0137 DOI: 10.1126/science.aau0137

Cap, Bernadette

2015 *Classic Maya Economies: Identification of a Marketplace at Buenavista del Cayo, Belize*.

PhD dissertation, University of Wisconsin-Madison.

Cap, Bernadette and Nathaniel James

2016 Initial Investigations of the Buenavista del Cayo Site Center Structure 3. In *Mopan Valley*

*Archaeology Project: Results of the* *2015 Field Season*, report submitted to the Belize

National Institute of Culture and History Institute of Archaeology, Belmopan.

Cap, Bernadette, Jason Yaeger, and M. Kathryn Brown

2018 Fidelity Tests of LiDAR Data for the Detection of Ancient Maya Settlement in the Upper

Belize River Valley, Belize. *Research Reports in Belizean Archaeology* 15:39-51.

Cap, Bernadette, Jason Yaeger, Rebecca Morris, and Devonshire Locke

2018 Buenavista del Cayo Structure 3 Summit Excavations: 2017 Findings. In *Mopan Valley*

*Archaeology* *Project: Results of the 2017 Field* *Season*, edited by Jason Yaeger, M.

Kathryn Brown and Bernadette Cap. Report submitted to the Belize National Institute of Culture and History Institute of Archaeology, Belmopan.

Carvajal, Pedro

2016 *The Settlement Patterns at Las Cuevas, Belize during the Late Classic Period*. Diss.

University of California, Merced.

Chase, Adrian S.Z.

2016a Beyond elite control: residential reservoirs at Caracol, Belize. *WIREs Water* 3(6):885-897. doi:10.1002/wat2.1171

2016b Districting and Urban Services at Caracol, Belize: Intrasite Boundaries in an Evolving Maya Cityscape. *Research Reports in Belizean Archaeology* 13:15-28.

2021 *Urban Life at Caracol, Belize: Neighborhoods, Inequality, Infrastructure, and Governance,* School of Human Evolution and Social Change, Arizona State University, Tempe.

2022 Sociopolitical Integration in Prehispanic Neighborhoods*.* In *Sociopolitical Integration in Prehispanic Neighborhoods*, edited by Gabriela Cervantes and John Walden. Center for Comparative Archaeology, Pittsburgh. *Forthcoming.*

Chase, Adrian S.Z. and John F. Weishampel

2016 Using LiDAR and GIS to Investigate Water and Soil Management in the Agricultural Terracing at Caracol, Belize. *Advances in Archaeological Practice* 4(3):357-370. doi:10.7183/2326-3768.4.3.357

Chase, Arlen F.

1988 Jungle Surveying: Mapping the Archaeological Site of Caracol, Belize*.* In *P.O.B. (Point of Beginning)*, pp. 10-12,14,16,18,22,24. P.O.B. Publishing Company, Wayne, Michigan.

Chase, Arlen F. and Diane Z. Chase

1987 *Investigations at the Classic Maya City of Caracol, Belize, 1985-1987* 3. Pre-Columbian Art Research Institute, San Francisco, USA.

1994 *Details in the archaeology of Caracol, Belize: An Introduction. In Studies in the Archaeology of Caracol, Belize* Monograph 7. Pre-Columbian Art Research Institute, San Francisco, CA.

1996 The Organization and Composition of Classic Lowland Maya Society: The View from Caracol, Belize*.* In *Eighth Palenque Round Table, 1993*, edited by Merle Greene Robertson, Martha J. Macri and Jan McHargue, pp. 213-222. Pre-Columbian Art Research Institute, San Francisco.

1998a Late Classic Maya Political Structure, Polity Size, and Warfare Arenas*.* In *Anatomía de Una Civilización Aproximaciones Interdisciplinareias a la Cultura Maya*, edited by Andrés Ciudad Ruíz and y otros, pp. 11-29. Sociedad Espanola de Estudios Mayas, Madrid.

1998b Scale and Intensity in Classic Period Maya Agriculture: Terracing and Settlement at the "Garden City" of Caracol, Belize. *Culture & Agriculture* 20(2-3):60-77. doi:10.1525/cag.1998.20.2-3.60

2001 Ancient Maya Causeways and Site Organization at Caracol, Belize. *Ancient Mesoamerica* 12(2):273-281. doi:doi:null

2006 Before the Boom: Caracol’s Preclassic Era. *Research Reports in Belizean Archaeology* 3:41-57.

2007 “This is the End”: Archaeological Transitions and the Terminal Classic period at Caracol, Belize. *Research Reports in Belizean Archaeology* 4:13-27.

2014a Ancient Maya Houses, Households, and Residential Groups at Caracol, Belize. *Research Reports in Belizean Archaeology* 11:3-17.

2015 The Domestic Economy of Caracol, Belize: Articulating with the Institutional Economy in an Ancient Maya Urban Setting. *Research Reports in Belizean Archaeology* 12:15-23.

2016 Urbanism and Anthropogenic Landscapes. *Annual Review of Anthropology* 45:361-376. doi:10.1146/annurev-anthro-102215-095852

2020a The Materialization of Classic Period Maya Warfare: Caracol Stranger-Kings at Tikal*.* In *A Forest of History: The Maya after the Emergence of Divine Kingship*, edited by Travis W. Stanton and M. Kathryn Brown, pp. 20-48. University Press of Colorado, Boulder.

2021 The Transformation of Maya Rulership at Caracol, Belize. In *Maya Kingship: Rupture and Transformation from Classic to Postclassic Times*, edited by Tsubasa Okoshi, Arlen F. Chase, Philippe Nondedeo and Charlotte M. Arnauld, pp. 224-245. University Press of Florida, Gainesville.

Chase, Arlen F., Diane Z. Chase, Jaime J. Awe, John F. Weishampel, Gyles Iannone, Holley Moyes, Jason Yaeger, M. Kathryn Brown, Ramesh L. Shrestha and William E. Carter

2014 Ancient Maya Regional Settlement and Inter-Site Analysis: The 2013 West-Central Belize LiDAR Survey. *Remote Sensing* 6(9):8671-8695. doi:10.3390/rs6098671

Chase, Arlen F., Diane Z. Chase and Adrian S.Z. Chase

2020a The Maya City of Caracol, Belize: The integration of an anthropogenic landscape*.* In *The Maya World*, edited by Scott R. Hutson and Traci Ardren, pp. 344-363. Routledge, New York.

2021 A Decade Later: The Impact of Caracol LiDAR Surveys on Archaeological Interpretation in the Maya Area*.* In *Lowland Maya Settlement Patterns in the Age of Lidar*, edited by Marcello A. Canuto and Francisco Estrada-Belli. Middle American Research Institute, New Orleans.

Chase, Arlen F., Diane Z. Chase, John M. Morris, Jaime J. Awe and Adrian S.Z. Chase

2020b Archaeology and Heritage Management in the Maya Area: History and Practice at Caracol, Belize. *Heritage* 3(2):436-456.

Chase, Arlen F., Diane Z. Chase, Richard Terry, Jacob M. Horlacher and Adrian S.Z. Chase

2015 Markets Among the Ancient Maya: The Case of Caracol, Belize*.* In *The Ancient Maya Marketplace: The Archaeology of Transient Space*, edited by E. King, pp. 226-250. University of Arizona Press, Tucson, AZ.

Chase, Arlen F., Diane Z. Chase, John F. Weishampel, Jason B. Drake, Ramesh L. Shrestha, K. Clint Slatton, Jaime J. Awe and William E. Carter

2011 Airborne LiDAR, archaeology, and the ancient Maya landscape at Caracol, Belize. *Journal of Archaeological Science* 38(2):387-398. doi:10.1016/j.jas.2010.09.018

Chase, Diane Z. and Arlen F. Chase

2003 Texts and Contexts in Classic Maya Warfare: A Brief Consideration of Epigraphy and Archaeology at Caracol, Belize*.* In *Ancient Mesoamerican Warfare*, edited by M.K. Brown and T.W. Stanton, pp. 171-188. Alta Mira Press, Walnut Creek, CA.

2014b Ancient Maya Markets And The Economic Integration Of Caracol, Belize. *Ancient Mesoamerica* 25:239-250.

2017 Caracol, Belize, and Changing Perceptions of Ancient Maya Society. *Journal of Archaeological Research* 25(3):185-249. doi:10.1007/s10814-016-9101-z

2020b The Ancient Maya Economic Landscape of Caracol, Belize*.* In *The Real Business of Ancient Maya Economies: From Farmers’ Fields to Rulers’ Realms*, edited by Marilyn A. Masson, David A. Freidel and Arthur A. Demarest, pp. 132-148. University Press of Florida, Gainesville.

Chase, Diane Z., Arlen F. Chase and Adrian S.Z. Chase

2020c Caracol’s Impact on the Landscape of the Classic Period Maya: Urbanism and Complex Interaction in a Tropical Environment*.* In *Approaches to Monumental Landscapes of the Ancient Maya*, edited by Brett A. Houk, Barbara Arroyo and Terry G. Powis, pp. 109-130. University Press of Florida, Gainesville.

Con, M. J.
1981 *Laguna Francesca*. Vol. 100. Colección Cientifica. Instituto Nacional de Antropología e

Historia, Mexico City.

Conlon, James M., and Jennifer J. Ehret

2000 Ancient Maya Settlement at Baking Pot, Belize: Results of the Continually Expanding Survey Program in the Search for the End of the Final Frontier. In *The Belize Valley Archaeological Reconnaissance Project: A Report of the 1999 Field Season*, Vol Department of Anthropology Occasional Paper edited by Cameron S. Griffith, Reiko Ishihara, and Jaime J. Awe, pp. 43-54, Vol. 3. University of New Hampshire, Durham.

Dahlin, Bruce H., Anthony Andrews, Tim Beach, Clara Bezanilla, Patrice Farrell, Sheryl Luzzader-Beach, and Valerie McCormick

1998 Punta Canbalam in Context: A Peripatetic Coastal Site in Northwest Campeche, Mexico. *Ancient Mesoamerica* 9:1-15.

Dahlin, Bruce H., and Traci Ardren

2002 Modes of Exchange and Regional Patterns: Chunchucmil, Yucatan, Mexico*.* In *Ancient Maya Political Economies*, edited by Marilyn Masson, and David Freidel, pp. 249-284. Altamira, Walnut Creek, CA.

Davis-Salazar, K.

2003 Late Classic Maya Water Management and Community Organization at Copán,

Honduras. *Latin American Antiquity* 14:275-299.

De Montmollin, Olivier
1989 *Settlement survey in the Rosario Valley, Chiapas, Mexico*. Vol. 57. Papers of the New

World Archaeological Foundation. New World Archaeological Foundation, Brigham

Young University, Provo, Utah.

1995 *Settlement and politics in three classic Maya polities*. Monographs in world archaeology

no. 24. Prehistory Press, Madison, Wis.

2018 Upper Grijalva Basin Dataset. Comparative Archaeology Database, University of

Pittsburgh.

Deluca, Anthony and Jason Yaeger

2019 Pedestrian Survey in the Buenavista del Cayo Southeast Settlement Zone. In *Mopan*

*Valley Archaeology Project: Results of the 2019 Field Season*, edited by Jason Yaeger, M. Kathryn Brown, and Bernadette Cap. Report to be submitted to the Belize National Institute of Culture and History Institute of Archaeology, Belmopan.

Driver, W. David, and James F. Garber

2004 The Emergence of Minor Centers in the Zones Between Seats of Power. In *The Ancient Maya of the Belize Valley: Half a Century of Archaeological Research*, edited by James F. Garber, pp. 287-304. University Press of Florida, Gainesville.

Dunham, P.S.
1990 *Coming Apart at the Seams: the Classic Development and Demise of the Maya*

*Civilization (A Segmentary View from Xnaheb, Belize*). Unpublished Doctoral

Dissertation, State University of New York at Albany.

Ebert, Claire E., Julie A. Hoggarth, and Jaime J. Awe

2016 Integrating Quantitative Lidar Analysis Integrating Quantitative LiDAR Analysis and Settlement Survey in the Belize River Valley. *Advances in Archaeological Research* 4(3):284-300. <https://doi.org/10.7183/2326-3768.4.3.284>

Ebert, Claire E., Keith M. Prufer, Martha J. Macri, Bruce Winterhalder, and Douglas J. Kennett
2014 Terminal Long Count Dates and the Disintegration of Classic Period Maya Polities.

*Ancient Mesoamerica* 25(02):337–356. DOI:10.1017/S0956536114000248.

Ehret, Jennifer J.

1995 The Xunantunich Settlement Survey Test-Pitting Program*.* In *Xunantunich Archaeological*

*Project: The 1995 Field Season*, edited by Richard Leventhal and Wendy Ashmore, pp.

164-191. Report submitted to the Institute of Archaeology, Belize.

Eppich, Keith, Damien B. Marken and Elsa Damaris Menéndez

2022 A City in Flux: The Dynamic Urban Form and Functions of El Peru-Waka', Guatemala. In *Building an Archaeology of Maya Urbanism: Flexibility and Planning in the American Tropics*, edited by Damien B. Marken and M. Charlotte Arnauld. University Press of Colorado, Boulder. *Forthcoming.*

Fash, W.

1983 *Maya State Formation: A Case Study and Its Implications*. Unpublished Ph.D.

Dissertation, Department of Anthropology, Harvard University, Cambridge.

Fash, W.

2001 *Scribes, Warriors, and Kings: The City of Copan and the Ancient Maya*. Thames and

Hudson, London.

Fash, W., and K. Long

1983 Mapa Arqueologico del Valle de Copán. *Introducción a la Arqueología de* *Copán,*

*Honduras*. Proyecto Arqueologico Copán, Secretaria de Estado en el Despacho de

Cultura y Turismo, Tegucigalpa, D.C.

Fedick, Scott L.

1995 Land evaluation and ancient Maya land use in the Upper Belize River area, Belize,

Central America. *Latin American Antiquity* 6(1):16–34. <https://doi.org/10.2307/971598>

Fedick, Scott L. and Anabel Ford

1990 The Prehistoric Agricultural Landscape of the Central Maya Lowlands: An

Examination of Local Variability in a Regional Context. *World Archaeology* 22:18-

33.

Fialko, Vilma

2011 Asentamiento y fachadas escultoricas del sitio arqueológico Holtun, Petén, Guatemala. In *XXIV Simposio de Investigaciones Arqueológicas en Guatemala* 2010, edited by B. Arroyo, L. Paiz, A. Linares and A. Arroyave. Pp. 466-490. Museo Nacional de Arqueología y Etnología, Guatemala.

Fletcher, Roland
2009 Low-density, agrarian-based urbanism: A comparative view. *Insights* 2(4):1–19.

Ford, Anabel

2014 Using Cutting-Edge Lidar Technology at El Pilar Belize-Guatemala in Discovering

Ancient Maya Sites ∼ There is Still a Need for Archaeologists! *Research Reports in*

*Belizean Archaeology* 11: 271–280.

Ford, Anabel and Scott L. Fedick

1992 Prehistoric Maya Settlement Patterns in the Upper Belize River Area: Initial Results

of the Belize River Archaeological Settlement Survey. *Journal of Field Archaeology*

19:35-49.

Ford, Anabel, and Sherman Horn III

2017 El Pilar Monuments Retrospective and Prospective: Re-Discovering El Pilar.

*Research Reports in Belizean Archaeology* 14: 87–95.

2018a El Pilar. In *Encyclopedia of Global Archaeology*, edited by C. Smith. Springer,

Cham. DOI: 10.1007/978-3-319-51726-1_3195-1

2018b Above and Below the Maya Forest: Advanced Remote Sensing Technology Raises

Questions About Settlement and Land use. *Science* 361 (6409): 1313–1314.

Friedel, Rebecca

2021 *From Earth and Sky: Human-Plant Relationships in the Ancient Mopan River Valley*

*Socioecological System*. PhD dissertation, University of Texas at San Antonio.

Gerstle, A.

1987 Ethnic Diversity and Interaction at Copan, Honduras. In *Interaction on the* *Southeast*

*Mesoamerican Frontier*, edited by E. J. Robinson, pp. 462. vol. ii. BAR International

Series 327.

Guerra, Rafael A., and Jaime J. Awe

2017 Recent Investigations at the Major Center of Lower Dover in the Belize River Valley. *Research Reports in Belizean Archaeology* 14:241-248.

Gussinyer, J.
1973 Tercera temporada de salvamento arqueológico en la Presa de La Angostura, Chiapas.

*Revista del Instituto de Ciencias y Artes de Chiapas* 7.

Guzman, Rodrigo

2020 *Informe Final*. Temporada de campo 2019. Proyecto de Reconocimiento y Mapeo. Sitio Arqueologico Holtun. Aldea La Maquina, Flores, Peten. Informe presentado a la Dirección General de patrimonio Cultural y Natural y al Departamento de Monumentos Prehispanicos y Coloniales del Ministerio de Cultura y Deportes de Guatemala.

2019 Archaeological Reconnaissance and Mapping at the Yaxhá basing. Final Report. Submitted to the Trevor Colbourn Anthropology Endowment Fund board in fulfilment with the requirements of the award. Department of Anthropology, University of Central Florida.

2017 Mapa arqueológico de Holtun. In *Proyecto Arqueológico Holtun*, Informe No. 6, temporada 2016. K. J. Cardona, M. G. Callaghan, and G. Kovacevich eds. Inform submitted to the Institute of Anthropology and History of Guatemala.

Hall, Jay, and René Viel

2004 The Early Copan Classic Landscape: A View from the Preclassic. In Understanding

*Early Classic Copan*, pp. 17-28. University of Pennsylvania Museum of Archaeology and

Anthropology, Philadelphia.

Hammond, Norman
1975 *Lubaantun: A Classic Maya Realm*. Peabody Museum Monographs 2. Harvard

University Press, Cambridge, MA.

Hare, Timothy, Marilyn Masson, and Bradley Russell

2014a High-Density LiDAR Mapping of the Ancient City of Mayapán. *Remote Sensing* 6(9):

9064–85. <https://doi.org/10.3390/rs6099064>.

Hare, T.S.; Masson, M.A.; Peraza Lope, C.

2014b The Urban Cityscape. In *Kukulkan’s Realm: Urban Life at Ancient Mayapan*; Masson,

M.A., Peraza Lope, C., Eds.; pp. 149–192. The University of Colorado Press: Boulder,

CO, USA. https://doi.org/10.5876/9781607323204.

Hartshorn, Gary, Lou Nicolait, and Lynne Hartshorn

1984 *Belize: Country Environmental Profile-A Field Study*. Robert Nicolait and Associates,

Ltd., Belize City, Belize.

Healy, Paul F.

2020 Ancient Pacbitun: An Introduction to the Archaeological Site and Early Research. In *An*

*Archaeological Reconstruction of Ancient Maya Life at Pacbitun, Belize*, edited by Terry

G. Powis, Sheldon Skaggs, and George J. Micheletti, pp. 9-24. BAR International Series

2970, Oxford.

Healy, Paul F., and Kitty F. Emery (editors)

2014 *Zooarchaeology of the Ancient Maya Centre of Pacbitun (Belize).* Occasional Papers in

Anthropology No. 16. Trent University, Peterborough, Ontario.

Healy, Paul F., Christophe G.B. Helmke, Jaime J. Awe, and Kay S. Sunahara

2007 Survey, Settlement, and Population History at the Ancient Maya Site of Pacbitun, Belize.

*Journal of Field Archaeology* 32(1):17-39.

Heckenberger, Michael J., James B. Petersen, and Eduardo Goés Neves

1999 Village Size and Permanence in Amazonia: Two Archaeological Examples from Brazil.

*Latin American Antiquity* 10 (04): 353–76. https://doi.org/10.2307/971962.

Helmke, Christophe, and Jaime J. Awe

2012 Ancient Maya Territorial Organisation of Central Belize: Confluence of Archaeological

and Epigraphic Data. *Contributions in New World Archaeology* 4:59-90.

Helmke, Christophe, Joseph W. Ball, Patricia T. Mitchell, and Jennifer T. Taschek

2008 Burial BVC88-1/2 at Buenavista del Cayo, Belize: Resting Place of the Last King of

Puluul? *Mexicon* 30(2):43-49.

Helmke, Christophe, Claire E. Ebert, Jaime J. Awe, and Julie A. Hoggarth

2020 The Lay of the Land: A Political Geography of an Ancient Maya Kingdom in West-Central Belize. *Contributions in New World Archaeology* 12:9-54.

Helmke, Christophe and Carmen Ting

2020 The Terminal Classic at Pacbitun: Evidence from Moulded-carved Ceramics. In *An*

*Archaeological Reconstruction of Ancient Maya Life at Pacbitun, Belize*, edited by Terry

G. Powis, Sheldon Skaggs, and George J. Micheletti, pp. 185-194. BAR International

Series 2970, Oxford.

Hoggarth, Julie A.

2012 *Social Reorganization and Household Adaptation in the Aftermath of Collapse at Baking Pot, Belize*. Doctoral Dissertation, Department of Anthropology, University of Pittsburgh, Pennsylvania.

Hoggarth, Julie A., Brendan J. Culleton, Jaime J. Awe, and Douglas J. Kennett

2014 Questioning Postclassic Continuity at Baking Pot, Belize, Using Direct AMS 14 C Dating of Human Burials. *Radiocarbon* 56(3):1057-1075. <https://doi.org/10.2458/56.18100>

Hoggarth, Julie A., Jaime J. Awe, Eva Jobbová, and Christopher Sims

2010 Beyond the Baking Pot Polity: Continuing Settlement Research in the Upper Belize River Valley. *Research Reports in Belizean Archaeology* 7:171-182.

Horn III, Sherman and Anabel Ford

2019 Beyond the Magic Wand: Methodological developments and results from integrated

Lidar survey at the ancient Maya center El Pilar. *STAR: Science & Technology of*

*Archaeological Research* 5(2): 164-178. DOI: [10.1080/20548923.2019.1700452](https://doi.org/10.1080/20548923.2019.1700452)

Horn III, Sherman, Anabel Ford, and Paulino Morales

2020 A Neighbourly Day in the Beautywood? Exploratory spatial analysis of settlement

patterns at El Pilar. *Research Reports in Belizean Archaeology* 17: 341-352.

Houston, Stephen

1986 Problematic Emblem Glyphs: Examples from Altar de Sacrificios, El Chorro, Río Azul,

and Xultun. *Research Reports on Ancient Maya Writing* 3: 1–11.

1993 *Hieroglyphs and History at Dos Pilas. Dynastic Politics of the Classic Maya*. Austin:

University of Texas Press.

Hudson, Paul F.

2004 Geomorphic Context of the Prehistoric Huastec Floodplain Environments: Lower Pánuco

Basin, Mexico.” *Journal of Archaeological Science* 31 (6): 653–68.

https://doi.org/10.1016/j.jas.2003.06.002.

Hutson, Scott R.

2016 *Ancient Urban Maya: Neighborhoods, Inequality and Built Form* Gainesville, University Press of Florida.

2017 *Ancient Maya Commerce: Multidisciplinary Research at Chunchucmil*. University Pres of Colorado, Boulder.

Inomata, Takeshi

1995 *Archaeological Investigations at the Fortified Center of Aguateca, El Petén, Guatemala:*

*Implications for the Study of the Classic Maya Collapse*. PhD dissertation, Vanderbilt

University.

2003 War, Destruction, and Abandonment: The Fall of the Classic Maya Center of Aguateca,

Guatemala. In *The Archaeology of Settlement Abandonment in Middle America*, edited by

Takeshi Inomata and Ronald W. Webb, 43–60. Salt Lake City: University of Utah Press.

Inomata, Takeshi, J. MacLellan, D. Triadan, J. Munson, M. Burham, K. Aoyama, H. Nasu, F. Pinzon, and H. Yonenobu

2015 Development of Sedentary Communities in the Maya Lowlands: Coexisting Mobile

Groups and Public Ceremonies at Ceibal, Guatemala. *Proc Natl Acad Sci* 112: 4268–73.

https://doi.org/10.1073/pnas.1501212112.

Jamison, T.R.
1993 *Symbolic Affiliation, Architecture, and Settlement Patterns in Southern Belize: Nim Li*

*Punit and Xnaheb during the Late Classic.* Unpublished Doctoral Dissertation, State

University of New York at Albany.

Kalosky, Ethan, and Keith M. Prufer
2012 Recent results of settlement survey and hinterland household excavations at the classic

period site of Uxbenká, Toledo district, Belize. *Research Reports in Belizean*

*Archaeology.* 9:255–276.

King, R.B., I.C. Baillie, R.J. Grimble, M.S. Johnson, and G.L. Silva
1986 *Land Resource Survey of Toledo District, Belize*. Land Resource Development Centre,

Tolworth, U.K.

Kovacevich, Brigitte, Patricia Rivera Castillo, Michael G. Callaghan y Melvin Rodrigo Guzman.

2011 Investigaciones Arqueológicas en “Cabeza de Piedra”: Resultados de dos temporadas de campo en el sitio de Holtun, Guatemala. In *XXV Simposio de investigaciones arqueológicas en Guatemala*. Ministerio de Cultura y Deportes. Dirección general del patrimonio cultural y natural. Instituto de Antropología e Historia. Museo Nacional de Arqueología y Etnología. Asociación Tikal. Guatemala.

Kurnick, Sarah

2013 *Negotiating the Contradictions of Political Authority: An Archaeological Case Study*

*from Callar Creek, Belize*. PhD dissertation, University of Pennsylvania, Philadelphia.

LeCount, Lisa J. and Jason Yaeger (eds)

2010 *Classic Maya Provincial Politics: Xunantunich and its Hinterlands*. The University of

Arizona Press, Tucson.

Lee, Thomas A.
1984 Investigaciones arqueologicas recientes del clasico, postclasico, y colonial maya en

Chiapas: resumen e implicaciones. *Mesa Redonda, Sociedad Mexicana de Antropologia*

1:113–130.

Leventhal, Richard, Wendy Ashmore, Lisa J. LeCount, and Jason Yaeger

2010 The Xunantunich Archaeological Project, 1991-1997. In *Classic Maya Provincial*

*Politics: Xunantunich and its Hinterlands*, edited by Lisa J. LeCount and Jason Yaeger,

pp. 1-19. The University of Arizona Press, Tucson.

Lowe, G.W.
1983 Los olmecas, mayas, y mixe-zoques. In *Antropología e historia de los mixe-zoques y*

*mayas: homenaje a Frans Blom*, edited by Frans Ferdinand Blom, Lorenzo Ochoa,

Thomas A. Lee, and Jay I. Kislak Reference Collection (Library of Congress), pp. 125–

130. 1a ed. Universidad Nacional Autónoma de México, Instituto de Investigaciones

Filológicas, Centro de Estudios Mayas ; Brigham Young University, México : [Provo,

Utah].

Lowe, G.W., and J.A. Mason
1965 Archaeological Survey of the Chiapas Coast, Highlands and Upper Grijalva Basin.

*Handbook of Mesoamerican Indians* 2:195–235.

Marken, Damien B.

2015 Conceptualizing the Spatial Dimensions of Classic Maya States: Polity and Urbanism at El Perú-*Waka’*, Petén. In *Classic Maya Polities of the Southern Lowlands*, edited by Damien B. Marken & James Fitzsimmons, pp. 123-166. University Press of Colorado, Boulder.

Marken, Damien B., Zachary J. Cooper, Bailey Gemberling, Dúglas Pérez, and Robert Austin

2020 Reconocimiento Regional de El Perú-Waka’, 2019: Verificación y Análisis del Suelo del DEM de LiDAR de PAW, Meseta Este y Área de Influencia al norte del Núcleo Urbano. In *Proyecto Arqueológico El Perú-Waka’: Informe No. 17, Temporada 2019*, edited by Juan Carlos Pérez, Griselda Pérez Robles & Damien B. Marken, pp. 161-270. Fundación de Investigación Arqueológica Waka’, Guatemala City.

Marken, Damien B., Juan Carlos Pérez, Olivia Navarro-Farr, and Keith Eppich

2019 Ciudad del Ciempiés: Urbanismo, Límites y Comunidad en El Perú-Waka‘, Petén Guatemala. In *XXXII Simposio de Investigaciones Arqueológicas en Guatemala*, 2018, edited by Bárbara Arroyo, Luis Méndez Salinas, Gloria Ajú Álvarez, pp. 531-546. IDEAH, Guatemala City.

Marken, Damien B. and Matthew C. Ricker

2023 Fire, Earth, and Water: Settlement, Soils and Hydrology at El Perú-Waka’. In *Kingdom of the Centipede: New Archaeological Perspectives on the Classic Maya Center of El Perú- Waka’*, edited by Keith Eppich, Damien B. Marken, and David Freidel. University Press of Florida, Gainesville. *Forthcoming.*

Martínez, M., and C. Navarrete
1978 El salvamento arqueologico en el estado de Chiapas. *Revista Mexicana de Estudios*

*Antropológicos* 24:229–255.

Masson, M.A. and C. Peraza Lope

2014 Archaeological Investigations of an Ancient Maya Urban Place. In *Kukulkan’s Realm:*

*Urban Life at Ancient Mayapán*; Masson, M.A., Peraza Lope, C., Eds. pp. 1-38. The

University of Colorado Press: Boulder, CO, USA. <https://doi.org/10.5876/9781607323204>.

Masson, M.A. and C. Peraza Lope

2014a Archaeological Investigations of an Ancient Maya Urban Place. In *Kukulkan’s Realm:*

*Urban Life at Ancient Mayapán*; Masson, M.A., Peraza Lope, C., Eds. pp. 1-38. The University of Colorado Press: Boulder, CO, USA. https://doi.org/10.5876/9781607323204.

2014b Militarism, Misery, and Collapse. In *Kukulcan’s Realm: Urban Life at Ancient Mayapan*,

by Marilyn A. Masson and Carlos Peraza Lope, pp. 521-540. University Press of Colorado, Boulder.

2014c Reconstructing Complexity in Urban Life. In *Kukulcan's Realm: Urban Life at Ancient*

*Mayapan*, by Marilyn A. Masson and Carlos Peraza Lope, pp. 541-557. University Press

of Colorado, Boulder.

Masson, M.A.; Hare, T.S.; Peraza Lope, C.

2014 The Social Mosaic. In *Kukulkan’s Realm: Urban Life at Ancient Mayapán*; Masson,

M.A., Peraza Lope, C., Eds.; pp 193-267. The University of Colorado Press: Boulder,

CO, USA. <https://doi.org/10.5876/9781607323204>.

Masson, Marilyn A., Carlos Peraza Lope, and Timothy S. Hare

2008 *Proyecto los Fundamentos del Poder Econdmico de Mayapan. Temporadas 2001-2004.* Informe Final para el Consejo Nacional de Arqueologia de Mexico. University at Albany-SUNY/Centro INAH Yucatan. Albany, New York/Merida, Yucatan.

Micheletti, George J., and Terry G. Powis

2020 Exploring the Minor Centers of Pacbitun’s Hinterland. *Research Reports in Belizean*

*Archaeology* 17:361-372.

Miller Wolf, K. and C. Freiwald

2018 Re-interpreting Ancient Maya Mobility: A Strontium Isotope Baseline for Western

Honduras. *Journal of Archaeological Sciences Reports*. 20:799- 807.

Montgomery, Shane

2018 Settlement and Karstic Survey. In *Dreams at Cuevas: Report of the 5^th^ Year*

*Investigations of the Las Cuevas Archaeological Reconnaissance (LCAR),* edited by

Holley Moyes and John P. Walden, pp. 59-84. Institute of Archaeology, National

Institute of Culture and History, Belmopan, Belize.

2019 LCAR 2018 Summary of Settlement and Karstic Survey. In *Report of the 6^th^ Year*

*Investigations of the Las Cuevas Archaeological Reconnaissance (LCAR)*, edited by

Holley Moyes and Erin E. Ray. Institute of Archaeology, National Institute of Culture and History, Belmopan, Belize.

Moyes, Holley, and Shane Montgomery

2016 Mapping Ritual Landscapes Using Lidar: Cave Detection through Local Relief Modeling.

*Advances in Archaeological Practice* 4.3:249-267.

2019 Locating Cave Entrances Using Lidar-Derived Local Relief Modeling. *Geosciences*

9(98):1-22.

Moyes, Holley, Mark Robinson, Laura Kosakowsky, Barbara Voorhies, Rafael Guerra, Fabrizio Galeazzi, and Josué Ramos.

2012 *Sleeping Next to the Giant: Preliminary Investigations of the Las Cuevas Site, Chiquibul*

*Reserve, Belize: A Site Report of the 2011 Field Season*. Institute of Archaeology,

National Institute of Culture and History, Belmopan, Belize.

Munson, Jessica, Andrés G. Mejía Ramón, and Lorena Paiz Aragón

2019 Mapeo de Asentamientos En Alta Resolución Con Sistemas Aéreos No Tripulados En

Altar de Sacrificios, Guatemala. In *XXXII Simposio de Investigaciones Arqueológicas*

*En Guatemala, Tomo II*, edited by Barbara Arroyo, Luis Méndez Salinas, and Gloria Ajú Álvarez, 637–48. Guatemala City: Museo Nacional de Arqueología y Etnología.

Munson, Jessica, and Lorena Paiz Aragón

2020 *Informe Del Proyecto Geomorfológico de Asentamientos Antiguos Del Usumacinta*

*Superior, La Temporada de 2019*: Investigando La Historia Fluvial y Los Patrones de

Asentamiento de Altar de Sacrificios y Sus Alrededores. Mexico: Instituto Nacional de

Antropología e Historia.

Murtha, Timothy

2009 *Maya Terraced Agriculture: An Investigation of the Settlement Economy and Intensive Agricultural Landscape of Caracol, Belize*. Muller, Saarbruckan, Germany.

NASA/METI/AIST/Japan Spacesystems And U.S./Japan ASTER Science Team.

2019 ASTER Global Digital Elevation Model V003 [Internet. NASA EOSDIS Land Processes DAAC [cited 2021 Jul 14. Available from: <https://lpdaac.usgs.gov/products/astgtmv003/>]

Paiz Aragón, Lorena, and Jessica Munson

2017 Proyecto Altar de Sacrificios, Temporada 2017. *Informe Entregado a La Dirección*

*General Del Patrimonio Cultural y Natural. Guatemala City*: Instituto de Arqueológia e

Historia.

Penn, Malcolm G., David A. Sutton, and Alex Monroe

2004 Vegetation of the Greater Maya Mountains, Belize. *Systematics and Biodiversity* 2(1):21-

44.

Peraza Lope, Carlos A., Pedro C. Delgado Ku, and Barbara Escamilla Ojeda

1999 Trabajos de Mantenimiento y Conservation Arquitectonica en Mayapan, Yucatan: Informe de la Temporada 1998. Informe de Actividades al Consejo de Arqueologia del INAH. Centro INAH - Yucatan, Merida.

2001 Descubrimientos recientes en Mayapan, Yucatan. *Investigadores de la Cultura Maya* 9, tomo 11:285-293.

2002 Trabajos de Mantenimiento y Conservation Arquitectonica en Mayapan, Yucatan: Informe de la Temporada 1998. Informe de Actividades al Consejo de Arqueologia del INAH. Centro INAH - Yucatan, Merida.

Peraza Lope, Carlos A., Marilyn A. Masson

2014 Politics and Monumental Legacies. In *Kukulcan's Realm: Urban Life at Ancient*

*Mayapan*, by Marilyn Masson and Carlos Peraza Lope, pp. 39-104. University Press of

Colorado, Boulder. <https://doi.org/10.5876/9781607323204>.

Peraza Lope, Carlos A., Marilyn A. Masson, Timothy H. Hare, and Pedro Delgado Ku

2006 *The Chronology of Mayapan: New Radiocarbon Evidence*. Ancient Mesoamerica 17(2):

153-175. 10.1017/S0956536106060135.

Petrozza, Michael Louis

2015 *Archaeological Investigations of the Lower Dover Periphery, Cayo District, Belize, Central America*, Department of Anthropology, Unpublished Masters Thesis, Texas State University San Marcos.

Powis, Terry G.

2020 Middle Preclassic Maya Community Organisation at Pacbitun, Belize. In *An*

*Archaeological Reconstruction of Ancient Maya Life at Pacbitun, Belize*, edited by Terry

G. Powis, Sheldon Skaggs, and George J. Micheletti, pp. 25-39. BAR International Series 2970, Oxford.

Peuramaki-Brown, Meaghan

2012 *The Integration and Disintegration of Ancient Maya Urban Centres: Charting*

*Households and Community at Buenavista del Cayo, Belize.* PhD dissertation, University

of Calgary.

Pingel, Thomas J., Keith Clarke, and Anabel Ford

2015 Bonemapping: A Lidar Processing and Visualization Technique in Support of

Archaeology Under the Canopy. *Cartography and Geographic Information Science* 42

(S1): S18–S26.

Pollock, Harry E. D., Ralph L. Roys, Tatiana Proskouriakoff, and A. Ledyard Smith (editors)

1962 *Mayapan, Yucatan, Mexico*. Carnegie Institution of Washington, Washington.

Ponciano, Erick M.

1995 Recientes Descubrimientos en el Departamento de Peten: Sitio Arqueológico Holtun, Aldea La Maquina, Flores. In *VIII Simposio de Investigaciónes Arqueológicas en Guatemala*, 1994, edited by J.P. Laporte y H. Escobedo, pp.484-492. Museo Nacional de Arqueología y Etnología: Guatemala

Prufer, Keith M
2005 The Early Classic In Southern Belize: A Regional View From Uxbenka And Ek Xux.

*Research Reports in Belizean Archaeology* 2:169–178.

Prufer, Keith M., and Douglas J. Kennett
2020 The Holocene Occupations of Southern Belize. In *Approaches to Monumental*

*Landscapes of the Ancient Maya*, edited by Brett A. Houk, Barbara Arroyo, and Terry G.

Powis, pp. 16–38. University Press of Florida, Gainesville, FL.

Prufer, K.M., H. Moyes, B.J. Culleton, Andrew Kindon, and D.J. Kennett
2011 Formation of a complex polity on the eastern periphery of the Maya lowlands. *Latin*

*American Antiquity* 22(2):199–223.

Prufer, Keith M., and Amy E. Thompson
2014 Settlements as neighborhoods and districts at Uxbenká: the social landscape of Maya

community. *Research Reports in Belizean Archaeology* 11:281–289.

2016 LiDAR based analyses of anthropogenic landscape alterations as a component of the built

environment. *Advances in Archaeological Practice* 4(3):393–409.

Prufer, Keith M., Amy E. Thompson, and Douglas J. Kennett
2015 Evaluating Airborne LiDAR for Detecting Settlements and Modified Landscapes in

Disturbed Tropical Environments at Uxbenká, Belize. *Journal of Archaeological Science*

57:1–13.

Prufer, K.M., Amy E. Thompson, C.R. Meredith, J.M. Jordan, Claire E. Ebert, Brendan J. Culleton, Ethan Kalosky, Bruce Winterhalder, and Douglas J. Kennett
2017 The classic period Maya transitions from an ideal free to ideal despotic settlement system

at the middle-level polity of Uxbenká. *Journal of Anthropological Archaeology* 45:53–

68.

Ramos, Josue

2013 The Tale of Monkey Tail. In *2nd Report of the Las Cuevas Archaeological*

*Reconnaissance Project: The 2012 Field Season*, edited by Holley Moyes and Mark

Robinson, pp. 112-116. Institute of Archaeology, National Institute of Culture and

History, Belmopan, Belize.

Rice, Don S. and Prudence M. Rice

1980 The Northeast Peten Revisited. In *American Antiquity* 45(3): 432-454.

Richards-Rissetto, H.M.

2010 *Exploring Social Interaction at the Ancient Maya City of Copán, Honduras: A Multi-*

*scalar Geographic Information Systems (GIS) Analysis of Access and Visibility* Ph.D.

dissertation. The University of New Mexico

Richie, Clarence J.

1990 *Ancient Maya Settlement and Environment of the Eastern Zone of Pacbitun, Belize*,

Unpublished Master's thesis, Department of Anthropology, Trent University,

Peterborough, Ontario.

Rivero T., S. E.
1990 *Patrón de asentamiento rural en la región de San Gregorio, Chiapas, para el clásico*

*tardío*. Colección Científica 192. Instituto Nacional de Antropología e Historia, Mexico.

Rosenswig, Robert M., Deborah M. Pearsall, Marilyn A. Masson, Brendan J. Culleton, and Douglas J. Kennett

2014 Archaic Period Settlement and Subsistence in the Maya Lowlands: New Starch Grain and

Lithic Data from Freshwater Creek, Belize.” *Journal of Archaeological Science* 41: 308–

21. <https://doi.org/10.1016/j.jas.2013.07.034>.

Russell, Bradley W.

2008 *Postclassic Maya Settlement on the Rural -Urban Fringe of Mayapán, Yucatán, Mexico.*

PhD Dissertation, State University of New York at Albany.

Skaggs, Sheldon, George J. Micheletti, Michael Lawrence, Nicaela Cartegena, and Terry G. Powis

2020 Identification of an Ancient Maya Groundstone Production Site in the Periphery of

Pacbitun, Belize. In *An Archaeological Reconstruction of Ancient Maya Life at Pacbitun,*

*Belize*, edited by Terry G. Powis, Sheldon Skaggs, and George J. Micheletti, pp. 159-175.

BAR International Series 2970, Oxford.

Smith, A. Ledyard

1962 Residential and Associated Structures at Mayapan. In *Mayapan, Yucatan, Mexico*, edited by Harry E. D. Pol- lock, Ralph L. Roys, Tatiana Proskouriakoff, and A. Le- dyard Smith, pp. 165-320. Occasional Publication 619. Carnegie Institution of Washington, Washington.

1972 *Excavations at Altar de Sacrificios: Architecture, Settlement, Burials, and Caches*. Vol.

62, No. 2. Papers of the Peabody Museum of Archaeology and Ethnology. Cambridge:

Harvard University.

Smith, Robert E.

1971 *The Pottery of Mayapan*. Volume 66, Papers of the Peabody Museum of Archaeology and Ethnology, Harvard University, Cambridge, MA.

Spenard, Jon

2014 *Underground Identity, Memory, and Political Spaces: A Study of the Classic Period*

*Maya Ceremonial Karstscape in the Pacbitun Region, Cayo District, Belize*, Unpublished

Ph.D. dissertation. Department of Anthropology, University of California, Riverside.

Sunahara, Kay S.

1995 *Ancient Maya Settlement: The Western Zone of Pacbitun, Belize,* Unpublished Master's

thesis, Department of Anthropology, Trent University, Peterborough, Ontario.

Thompson, Amy E.
2019 *Comparative Processes of Sociopolitical Development in the Foothills of the Southern*

*Maya Mountains*. Unpublished Doctoral Dissertation, University of New Mexico.

2020 Detecting Classic Maya Settlements with Lidar-Derived Relief Visualizations. *Remote*

*Sensing* 12(17):2838. DOI:10.3390/rs12172838.

Thompson, Amy E., Gary M. Feinman, and Keith M. Prufer
2021 Assessing Classic Maya multi-scalar household inequality in southern Belize. *PLOS ONE*

16(3):e0248169. DOI:10.1371/journal.pone.0248169.

Thompson, Amy E., and Keith M. Prufer
2015 Airborne LiDAR for detecting ancient settlements, and landscape modifications at

Uxbenká, Belize. *Research Reports in Belizean Archaeology* 12:251–259.

2016 Preliminary Findings from Ix Kuku’il, Toledo District, Belize. *Research Reports in*

*Belizean Archaeology* 13.

2019 Archaeological Research in Southern Belize at Uxbenká and Ix Kuku’il. *Research*

*Reports in Belizean Archaeology*:311–322.

2021 Household Inequality, Community Formation, and Land Tenure in Classic Period

Lowland Maya Society. *Journal of Archaeological Method and Theory*:1–38.

Tibbits, Tawny

2020 Geochemical Sourcing of Granite Artefacts. In *An Archaeological Reconstruction of*

*Ancient Maya Life at Pacbitun, Belize*, edited by Terry G. Powis, Sheldon Skaggs, and

George J. Micheletti, pp. 175-184 BAR International Series 2970, Oxford.

von Schwerin, J., H. Richards-Rissetto, F. Remondino, M. Grazia Spera, M. Auer, N. Billen, L. Loos, and M. Reindel

2016 Airborne LiDAR Acquisition, Post-Processing and Accuracy-Checking for a 3D WebGIS

of Copan, Honduras. *Journal of Archaeological Science Reports* 5: 85-104.

doi:10.1016/j.jasrep.2015.11.005.

Walden, John P.

2021 *The Dynamics of Political Eclipse: The Shifting Strategies of Ancient Maya Intermediate*

*Elites at Lower Dover, Belize*. PhD Dissertation. Department of Anthropology.

University of Pittsburgh, PA.

Walden, John P., Claire E. Ebert, Julie A. Hoggarth, Shane M. Montgomery, and Jaime J. Awe

2019 Modeling Variability in Classic Maya Intermediate Elite Political Strategies Through Multivariate Analysis of Settlement Patterns. *Journal of Anthropological Archaeology* 55:101074. <https://doi.org/10.1016/j.jaa.2019.101074>

Walden, John P., Tia B. Watkins, Kyle Shaw-Müller, Claire E. Ebert, Emma Messinger, Rafael A. Guerra, and Awe Jaime J.

2020 Multiscalar Approaches to Reconstructing Classic Maya Strategies of Ceremonial

Inclusion and Exclusion through the Accessibility of Architecture at Lower Dover,

Belize. In *El paisaje urbano maya: del preclásico al virreinato*, edited by Juan Garcia

Targa, and Geiser Gerardo Martín Medina, pp. 195-218. British Archaeological Reports,

International Series, Oxford.

Weber, Jennifer

2011 *Investigating the Ancient Maya Landscape: A Settlement Survey in the Periphery of*

*Pacbitun, Belize*. Unpublished Master’s thesis, Department of Anthropology, Georgia

State University, Atlanta.

Weeks, John (editor)
2009 *The Carnegie Maya II: Carnegie Institution of Washington Current Reports, 1952-1957.* University of Colorado Press, Boulder.

Wernecke, D. Clark

2005 *A stone canvas: Interpreting Maya building materials and construction technology*.

Ph.D. dissertation, Department of Anthropology, University of Texas, Austin, TX.

Willey, Gordon R., William R. Bullard Jr., John B. Glass, and James C. Gifford

1965 *Prehistoric Maya Settlements in the Belize Valley*. Papers of the Peabody Museum of Archaeology and Ethnology 54. Harvard University, Cambridge.

Willey, G. and R. Leventhal

1978 Maya Settlement in the Copan Valley. *Archaeology* 31(4):32-43.

Wright, A.C.S., D.H. Romney, R.H. Arbuckle, and V.E. Vial
1959 *Land in British Honduras Land Use Survey Team*. Colon. Res. Publ. No 24. Her

Majesty’s Stationary Office, London, London, UK.

Yaeger, Jason, M. Kathryn Brown, and Bernadette Cap.

2016 Locating and Dating Sites Using LiDAR Survey in a Mosaic Vegetative Environment in

Western Belize. *Advances in Archaeological Practice* 4:339-356.

Zetina Gutiérrez, María de Guadalupe, and Betty Bernice Faust

2011 De la Agroecología a la Arqueología Demográfica: ¿Cuántas Casas por Familia?

*Estudios de Cultura Maya* 38:97-120.
